# A tetraphenylborate-based anionic metal–organic framework as a versatile solid electrolyte for fast Li^+^, Na^+^, K^+^, Mg^2+^, Ca^2+^, and Zn^2+^ transportation†

**DOI:** 10.1039/d4sc02861a

**Published:** 2024-10-07

**Authors:** Qingchun Xia, Kaixin Han, Xuxiao Ma, Pengtao Qiu, Zhiyong Li, Xuenian Chen

**Affiliations:** a Henan Key Laboratory of Boron Chemistry and Advanced Energy Materials, Collaborative Innovation Center of Henan Province for Green Manufacturing of Fine Chemicals, Key Laboratory of Green Chemical Media and Reactions, Ministry of Education, School of Chemistry and Chemical Engineering, Henan Normal University Xinxiang Henan 453007 China xiaqingchun@htu.edu.cn qiupengtao@htu.edu.cn xuenian_chen@zzu.edu.cn; b College of Chemistry, Zhengzhou University Zhengzhou 450001 China

## Abstract

Tetraphenylborate (BPh_4_^−^) has been widely employed in the field of electrolytes and displayed better ionic conductivities in polymer solid-state Li^+^ conductors. However, the fabrication of tetraphenylborate monomers into metal–organic frameworks (MOFs) and the exploration of their potential in solid-state electrolytes have never been reported. In this work, carboxylic acid functionalized lithium tetraphenylborate was purposefully synthesized and employed to construct an anionic MOF as a solid electrolyte. The counter cation Li^+^ was encapsulated into the anionic channel to become the free mobile charge carrier that produced a lithium-ion solid electrolyte with outstanding ion conductivity (2.75 × 10^−3^ S cm^−1^ at 25 °C), an impressively high lithium-ion transference number (*t*_Li^+^_ = 0.89), and low activation energy (0.15 eV). Acting as a solid electrolyte, the anionic MOF-based lithium iron phosphate battery delivered an initial specific capacity of 135 mA h g^−1^ and retained 95% capacity after 220 charge–discharge cycles with a coulombic efficiency close to 100%. Moreover, by exchanging the free Li^+^ with Na^+^, K^+^, Mg^2+^, Ca^2+^, and Zn^2+^, our anionic MOF is also available for other types of solid electrolytes with the corresponding conductivities all above that of the functional battery electrolyte. Our work provided a convenient and tunable route to prepare conducting MOFs for alkali metal ions, alkaline earth metal ions, and other possible metal cations of interest, which could be used in solid-state electrolytic devices in the future.

## Abstract 2

Tetraphenylborate (BPh_4_^−^) has been widely employed in the field of electrolytes and displayed better ionic conductivities in polymer solid-state Li^+^ conductors.

## Graphical abstract

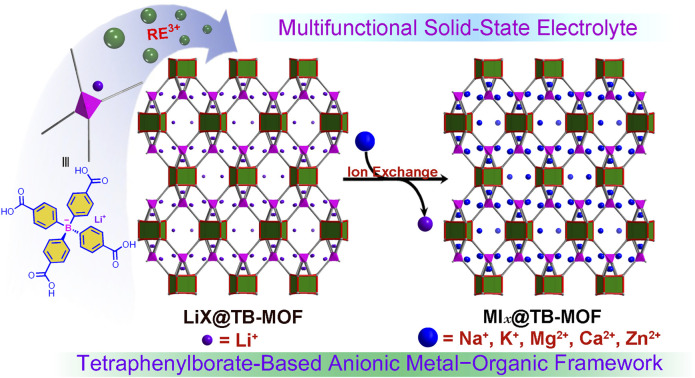

## Introduction

Solid-state batteries (SSBs) are regarded as the future of batteries due to the use of the solid-state electrolyte (SSE) in SSBs without the potential risks of volatilization, leakage, and fire.^1–3^ Among the various SSEs, metal–organic frameworks (MOFs) are considered one of the promising candidates,^4,5^ because they are electrical insulators, compatible with a wide range of charge carriers, and have regular pores/channels for swift charge ion transport.^6–9^ Although much progress has been made recently, nearly all these MOFs need additional metal salts, such as LiClO_4_, Mg(TFSI)_2_*etc.*^4,10–13^ This implies that both cations and anions are mobile in the channel and the mobility of the anion will reduce the cation transference number and lead to the polarization effect.^14–18^ If things go on like this, poor cell performances, including voltage loss and high internal impedance, may ultimately lead to the inevitable cell failure.^19–22^ To avoid this, a handful of MOF-based single-ion SSEs have been developed.^5,7,17,19,20^ However, most of them just involve the immobilization of anions *via* post-synthetic modification, where the anions are either coordinately bonded to metal clusters or covalently linked to ligands.^4,14,17,19^ This necessarily means that not all ligands and metal clusters within the framework can be modified to provide continuous hopping sites for charge carriers and, therefore, still display inferior ionic transference numbers and high activation energy.

Anionic MOFs, as a subclass of MOFs with the negative charge centers being uniformly located in the frameworks,^5,7,20,23–28^ are regarded as an ideal platform for the single-ion SSE, distinguishing themselves from other materials. In anionic MOFs, the uniform negative charge centers can provide continuous hopping sites for charge carriers, thus effectively reducing the activation energy and significantly increasing the cation transference number.^7,20,29^ Beyond that, the counter cation can be exchanged with other exogenous ions, such as H^+^, Li^+^, Na^+^, K^+^, Mg^2+^, Ca^2+^, and Zn^2+^, giving the anionic MOF new ion conduction functionalities.^5,7,20,30^ Although some recent progress has been made in anionic MOFs, the routes to build up an anionic framework are often accidental.^7,20,29–31^ The direct use of anionic ligands allows for the purposeful synthesis of anionic MOFs. However, they are still rare because anionic ligands are so scarce that only one such reported anionic MOF was investigated, which was employed as an anionic platform to take up [Ru(bpy)_3_]^2+^ for photocatalysis.^32^

Lithium tetraphenylborate (LiBPh_4_) has been utilized in single-ion electrolyte fields.^33,34^ In tetraphenylborate, the negative charge is spread out over the whole molecule rather than localized on the central B atom, and the four benzene rings disperse about 80% of the negative charge.^35,36^ As a result, there is a significant decrease in the coulombic attraction between the BPh_4_^−^ anion and metal cation, and such a decrease thus effectively increases the propensity of charge separation and reduces the activation energy of charge transfer.^37^ Furthermore, the extreme size difference makes the aggregation of the simplest ion difficult.^38^ Moreover, the relatively low lattice energy and the great polarizability make tetraphenylborate salt have little tendency to form contact pairs and low ion-dipole stabilization energy.^39,40^ Therefore, the tetraphenylborate monomer is considered one of the best candidates for the fabrication of anionic MOFs for solid-state electrolytes.

In this work, we report the synthesis of a microporous anionic MOF based on carboxylic acid functionalized lithium tetraphenylborate. Counter Li^+^ cations are encapsulated into the anionic MOF and serve as the free mobile charge carrier that produces a lithium-ion solid electrolyte. After introducing additional LiI into the pore of the anionic MOF and grafting the I^−^ onto metal clusters, the resulting modified MOF demonstrates excellent ionic conductivity (2.75 × 10^−3^ S cm^−1^ at 25 °C), an impressive lithium-ion transference number (*t*_Li^+^_ = 0.89), and low activation energy (0.15 eV). This anionic MOF-based lithium iron phosphate SSB delivers an initial specific capacity of 135 mA h g^−1^ and 95% capacity retained at 0.5 C after 220 cycles with a coulombic efficiency close to 100%. Exchange of the free Li^+^ with Na^+^, K^+^, Mg^2+^, Ca^2+^, or Zn^2+^ leads to the high mobility of these charge carriers. The ionic conductivities of these solid-state electrolytes (3.09, 1.64, 3.28, 1.30, and 6.10 × 10^−4^ S cm^−1^ at room temperature) are all above that of the functional battery electrolyte. Our work not only sheds new light on the preparation of MOF-based superionic conductors but also helps us to understand the structure–conductivity relationship for high-performance lithium and other metal SSEs, starting from fundamental molecular design and construction. Most significantly, we believe that our strategy is also popularized with other anionic MOFs, which would open a new way for exploring novel SSEs for safe and long-life solid-state energy-storing fields.

## Results and discussion

### Synthesis and characterization

The newly designed ligand Li·[H_4_L] was prepared in 73% overall yield through three-step reactions by using 1,4-diiodobenzene and BF_3_·Et_2_O as starting materials. The colorless rodlike single crystals of Li_2_·[Er_3_L_2_(HCOO)(DMF)_2_(H_2_O)]·nG (TB-MOF: Tetrahedral Boron Metal–Organic Frameworks) were obtained in about 84% yield by heating a mixture of Li·[H_4_L], ErCl_3_·6H_2_O and formic acid (FA) with a molar ratio of 1 : 1 : 0.05 in a mixture of DMF and MeOH at 80 °C for 24 h. Other salts LnCl_3_·6H_2_O (Ln = Sm, Eu, Ga, Tb, Dy, Ho, Tm, Yb, and Lu) could also afford the same topologies. Their formulations were determined based on the microstructure analysis such as single crystal X-ray diffraction (SC-XRD), FT-IR spectroscopy, elemental analysis (EA), inductively coupled plasma mass spectrometry (ICP-MS), and thermogravimetric analysis (TGA).

**Fig. 1:**
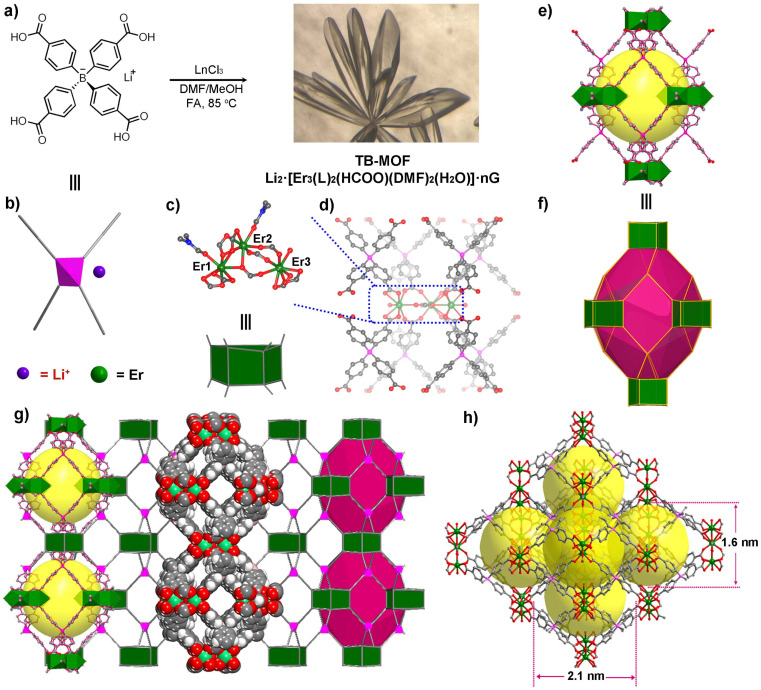
(a) Construction of TB-MOF with the anionic tetraphenylborate ligand Li·[H_4_L], and a microscopic image of the crystal. (b) Molecular structure of Li·[H_4_L]. (c and d) Coordination environments of erbium ions and the 8-connected Er_3_ cluster in TB-MOF. (e and f) The distorted and simplified dodecahedral cage in TB-MOF. (g and h) 3D porous structure of TB-MOF (green Er, red O, blue N, and gray C).

SC-XRD analysis performed on TB-MOF unambiguously confirmed the formation of an anionic 3D open metal–organic network. TB-MOF crystallizes in the orthogonal space group *Pnma*, with one tetraphenylborate ligand and three crystallographically independent Er^3+^ (labeled as Er1, Er2, and Er3, respectively, Fig. 1c) in the asymmetric unit. As shown in Fig. 1c and d, the three Er atoms are clustered by four bidentate-bridging and four chelated carboxylate groups from eight BPh_4_^−^ ligands to form a trierbium cluster, in which two DMF and one H_2_O molecule are coordinated to Er1, Er2, and Er3, respectively. Notably, these three Er atoms are additionally bonded by a tridentate formate group. For the BPh_4_^−^ ligand, the central B adopts sp^3^ hybridization but is significantly distorted from the general *T*_d_ symmetry with a small (102.9(5)°) C–B–C angle between the two face-to-face phenyl groups. Each BPh_4_^−^ ligand is linked to four trierbium clusters, and each trierbium cluster is linked by eight BPh_4_^−^ ligands, forming an anionic (4,8)-connected flu network with the shortest distance among the B centers of ∼9.60(0) Å (Fig. S3†). In the structure, a distorted dodecahedral cage with cavity dimensions of ∼14.0 × 18.2 × 20.4 Å^3^ is formed from six trierbium clusters occupying the vertices and eight BPh_4_^−^ ligands riding on the faces (Fig. 1e and f). Each cage is surrounded by 12 adjacent cages *via* sharing faces, thus generating a 3D porous structure, filled with the charge-balancing Li^+^ cations and guest molecules. Along the *a*- and *c*-axes, interconnected 1D open channels of ∼12.1 × 9.1 and ∼12.1 × 10.9 Å^2^ are demonstrated in the porous structure, respectively.

As Li^+^ exists in the anionic network, the precise position of Li^+^ cannot be resolved by SC-XRD, which is probably ascribed to the Li^+^ ion solvated by the guest molecule and disordered in the cavity. The presence of Li^+^ was confirmed by the ^7^Li magic-angle spinning nuclear magnetic resonance (^7^Li MAS NMR) spectrum (Fig. 3a, and S9†). Only a sharp single peak was absolved at −1.01 ppm, implying the liquid-like fast motion of the lithium species. At the same time, ICP-MS analysis of the digested TB-MOF showed that the content of the Li^+^ in the anionic frameworks is 0.91 wt%, unambiguously confirming the existence of Li^+^ inside the MOF. Significantly, varying the ionic Li·[H_4_L] ligand to the neutral tetrakis(4-carboxyphenyl)methane ligand can also afford the same structure, denoted as TC-MOF, but it is a charge-neutral framework. SC-XRD revealed that TC-MOF is isostructural to TB-MOF and has an almost identical 3D structure.

### Porosity and stability

**Fig. 2:**
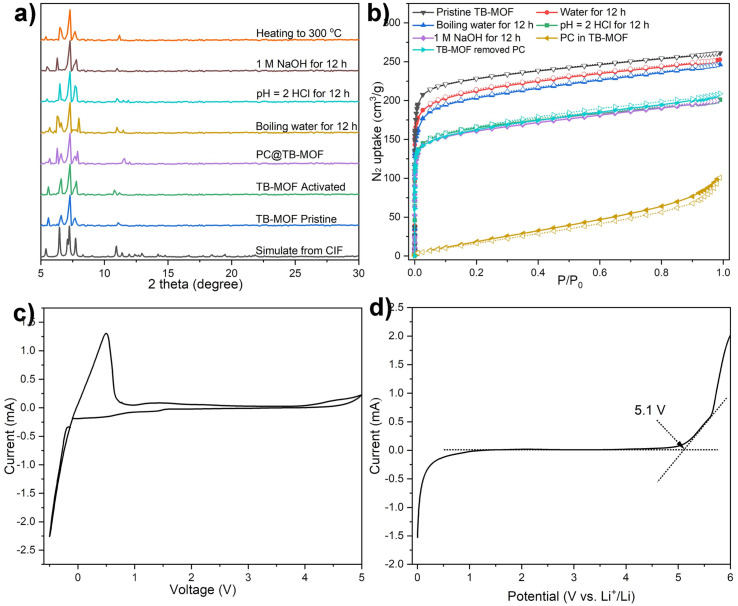
(a) PXRD patterns of TB-MOF under different conditions. (b) The corresponding N_2_ adsorption isotherms at 77 K. (c) CV and (d) LSV measurements for the SS/TB-MOF/Li asymmetric cells at room temperature at a scan speed of 5 mV s^−1^.

PLATON calculation indicates that the corresponding solvent accessible volume for TB-MOF is about 56% by summing voxels more than 1.2 Å away from the framework.^41^ The phase purity was confirmed by comparing their observed and simulated PXRD patterns (Fig. 2a). TGA analysis showed that the included guest molecules in the framework could be readily removed in a temperature range of 80 to 200 °C, and the framework was stable at temperatures up to 470 °C (Fig. S10†). PXRD indicated that the structure and crystallinity integrity were intact after the removal of the guest molecules. Furthermore, variable temperature PXRD (VT-XRD) demonstrated that the structure and crystallinity integrity could be preserved up to 300 °C, consistent with the TGA result (Fig. 2a). Nitrogen adsorption measurements revealed a Type-I sorption behavior with a BET surface area of 812 m^2^ g^−1^ (Fig. 2b). The chemical stability of TB-MOF in the presence of water, acid, and base was then investigated by PXRD, N_2_ adsorption, and ^11^B NMR. As shown in Fig. 2a, the PXRD patterns remained intact after being treated with water (100 °C), aqueous HCl (pH = 2), and NaOH solution (1.0 mol L^−1^) for 12 h, and the BET surface areas were then estimated to be 781, 620, and 628 m^2^ g^−1^, respectively. The ^11^B NMR spectrum of these digested samples displayed only one boron signal with the chemical shift almost the same as that of the tetraphenylborate ligand, thus confirming the robust B–C bond and no B–C-cleavage, phase transition, or framework collapse during these harsh treatments (Fig. S12†). Notably, compared with the neutral framework TC-MOF that can only preserve its structure and crystallinity integrity in 0.01 mol L^−1^ NaOH solution (Fig. S14 and S15†), the stability of TB-MOF in an alkaline solution is significantly improved at least 100 times compared to that of TC-MOF. This improvement probably ascribes to the electrostatic repulsion that the negatively charged skeleton in TB-MOF can effectively protect the trierbium clusters from the attack of OH^−^ in the alkaline solution.^42^

Electrochemical stability is another essential characteristic to determine whether TB-MOF can be safely applied as a solid electrolyte. The electrochemical working window was evaluated in a stainless steel/TB-MOF/Li cell by cyclic voltammetry (CV) and linear sweep voltammetry (LSV) measurements at room temperature. As shown in Fig. 2c and d, the CV curve displayed only a stable Li plating/stripping process at ∼0 V *vs.* Li/Li^+^, and a wide voltage window up to 5.0 V was observed. The LSV curve exhibited a stable electrochemical window with a decomposition voltage of ∼5.1 V. Both CV and LSV measurements highlight the excellent electrochemical stability of TB-MOF towards the Li metal and high-voltage cathode, which is sufficient for the practical application of high energy density batteries.

### Li^+^ conductivity

With the advantages of high thermal, chemical, and electrochemical stabilities and possessing a unique porous and anionic nature, the Li^+^-containing TB-MOF is envisioned as a competitive candidate for a single Li-ion solid-state conductor. Before the ionic conductivity measurement, TB-MOF was immersed in propylene carbonate (PC) solvent for 24 h, washed with THF, and dried under vacuum to afford free-flowing dry powder. The incorporation of the PC solvent into TB-MOF was confirmed by N_2_ adsorption measurements. As shown in Fig. 2b, the BET surface area was significantly decreased to 78 m^2^ g^−1^. The PC content was estimated by TGA analysis and calculated to be ∼53 wt%, which indicated and anionic framework with about 19 PC molecules in each nanocage. These results also revealed that the free Li^+^ ions in TB-MOF were highly solvated by PC molecules. PXRD further demonstrated that the crystallinity was well retained after the incorporation of the PC molecules (Fig. 2a). Interestingly, the BET surface area could be nearly recovered to 628 m^2^ g^−1^ when the encapsulated PC was removed by washing with hot MeOH several times.

**Fig. 3:**
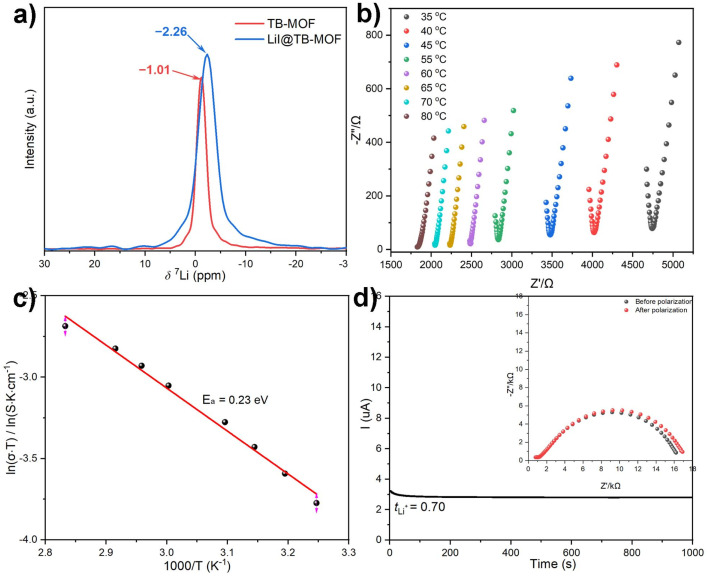
(a) ^7^Li MAS NMR spectra of TB-MOF and LiI@TB-MOF. (b) Nyquist plots for TB-MOF. (c) Ionic conductivity as a function of temperature. (d) Current–time curve for the Li/TB-MOF/Li cell at a DC polarization of 10 mV. Inset: Nyquist plots obtained before and after polarization.

Under an argon atmosphere, the dry powder sample was mechanically pressed into a pellet and sandwiched between two stainless steel electrodes in an airtight cell. The ionic conductivity was measured by electrochemical impedance spectroscopy (EIS) at temperatures from 35 to 80 °C with 5 °C intervals. As shown in Fig. 3b, the Nyquist plots for TB-MOF showed an incomplete semicircle at high frequency and a linear tail at low frequency, with the ionic resistance being determined to be 4.7 kΩ. The ionic conductivity was calculated to be 6.42 × 10^−5^ S cm^−1^ at 35 °C and 1.17 × 10^−4^ S cm^−1^ at 50 °C. The ionic conductivity was further found to scale with temperature and showed Arrhenius character, which indicates that a hopping mechanism is responsible for the lithium transportation whereby the Li^+^ ions diffuse to the nearest BPh_4_ centers.^8,43^ Fitting the corresponding temperature-dependent conductivity curves provided an activation energy of 0.23 eV (Fig. 3c). Direct current (DC) polarization measurements gave an electronic conductivity of about 7.3 × 10^−9^ S cm^−1^ (Fig. S16†), illustrating the negligible contribution of the electron conduction. Control experiments performed on the neutral TC-MOF and Li·[Me_4_L] ester afforded an ionic conductivity of 1.78 × 10^−7^ and 1.02 × 10^−8^ S cm^−1^, respectively, demonstrating the vital role of the highly free Li^+^ ions and the well-defined pore channels in the ion conduction (Fig. S17 and S18†). The Li^+^ transference number (*t*_Li^+^_) is another crucial parameter for evaluating the contribution of Li^+^ conduction to ionic conductivity. Chronoamperometry measurements revealed that the *t*_Li^+^_ was calculated to be as high as 0.70, indicating that Li^+^ ionic conductivity makes the biggest contribution (Fig. 3d).

**Fig. 4:**
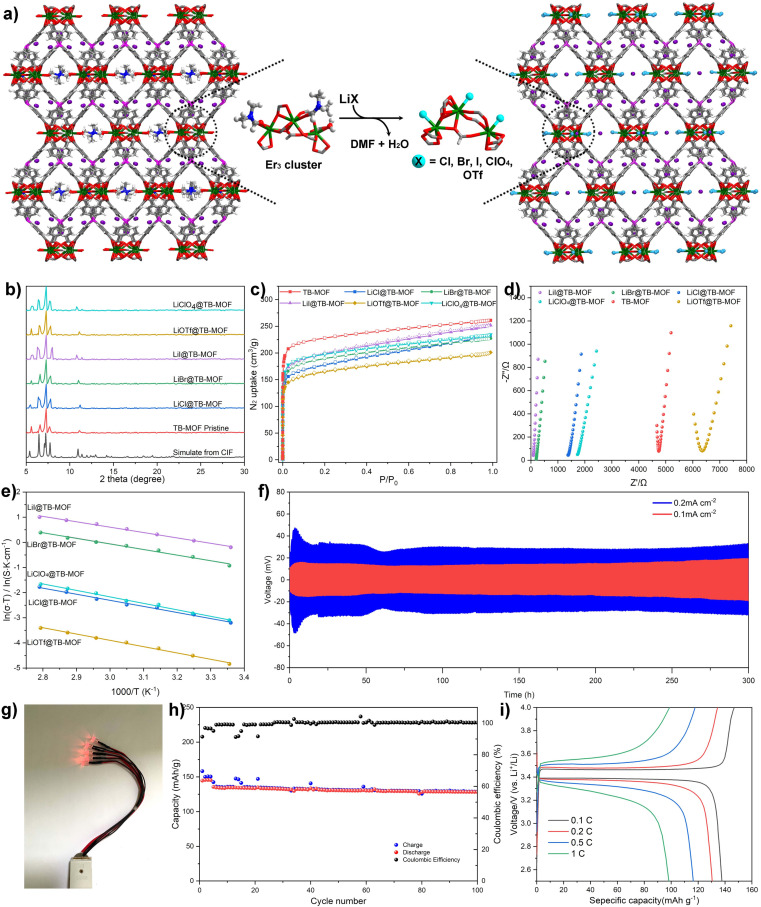
(a) Schematic illustration of grafting LiX (X = Cl^−^, Br^−^, I^−^, ClO_4_^−^, and OTf^−^) salts to replace the DMF and water molecules. (b) PXRD and (c) N_2_ adsorption isotherms of LiX@TB-MOF. (d) Nyquist plots for LiX@TB-MOF. (e) Ionic conductivities as a function of temperature in the range of 35 to 80 °C. (f) Voltage profiles of Li stripping/plating for a Li/LiI@TB-MOF/Li symmetrical cell during the galvanostatic cycling with a current density of 0.1 mA cm^−2^ and 0.2 mA cm^−2^ at room temperature. (g) The obtained Li/LiI@TB-MOF/LFP SSB for lighting LED lamps. (h) Cycling performance and coulombic efficiency of the Li/LiI@TB-MOF/LFP SSB with a 0.5 C charge/discharge rate at room temperature (the initial four cycles were conducted with 0.1 C to activate the SSB). (i) Charge–discharge voltage profiles of the Li/LiI@TB-MOF/LFP SSB cell at different C-rates at room temperature.

It is well known that ionic conductivity strongly relies on the concentration of Li^+^ ions and the conductive paths in anionic channels.^44,45^ In TB-MOF, the content of the Li^+^ ions is theoretically calculated to be 0.82 wt%. It has been reported that soaking MOF electrolyte into the PC solution of LiBF_4_ and/or mixing MOF electrolyte with LiTFSI can not only increase the Li^+^ content in MOF electrolyte but also conduct the Li^+^ along the interface,^19^ as well as help Li^+^ migration through the grain boundary,^4^ thereby significantly improving the ionic conductivity. However, the conductivities of our TB-MOF were still as limited to 5.48 × 10^−5^ and 6.03 × 10^−5^ S cm^−1^, respectively. Therefore, we reason that the bulk resistance mainly arises from the internal resistance that the directional migration of Li^+^ ions encounters inside the anionic channel. For the conductive paths, the shortest distance of the negatively charged centers BPh_4_ in TB-MOF is measured to be 9.60(0) Å, which is sixteen times larger than the radius of Li^+^ (0.6 Å). This distance is probably a little far away and may be unfavorable for efficient site-to-site Li^+^ hopping.^38^ Beyond that, as shown in Fig. 1c and 4a, TB-MOF contains three coordinated solvent molecules (two DMF molecules and one H_2_O molecule) in each trierbium cluster, and the two bulk DMF molecules block the anionic channels in the *a* and *c* axes separately (Fig. S7†). This structural feature may contribute to its inferior ion conductivity to a certain extent. As is well known, these coordinating molecules are labile and can be readily removed from the trierbium clusters without affecting the integrity of the lattice. However, the ionic conductivity was still limited to 1.02 × 10^−4^ S cm^−1^ at 35 °C (Fig. S19†).

Upon heating TB-MOF under dynamic vacuum and subsequently treating with a THF solution of LiI, TB-MOF with an I-decorated trierbium cluster was then provided (denoted as LiI@TB-MOF). During this process, the neutral [Er_3_(O_2_C)_8_(HCOO)(DMF)_2_(H_2_O)] cluster is modified to the anionic [Er_3_(O_2_C)_8_(HCOO)I_3_]^3−^ cluster (Fig. 4a), and additional Li^+^ ions are forced into the framework at the same time with the contents increasing from 0.82 wt% to 1.80 wt%, theoretically. Combined with the negative BPh_4_ center, there are two negatively charged centers in LiI@TB-MOF, and the distance between the B center and I is measured to be 6.71 Å. Such post-synthesis modification not only provides a high density of Li^+^ ions near each other in the anionic channels but also increases hopping sites and shortens the hopping distance. Most importantly, we surmise that the channel blocking DMF molecules grafted with the small I^−^ anions open more channels to facilitate Li^+^ transportation.

PXRD first indicated that the structure and crystallinity integrity of TB-MOF could be preserved in the resulting LiI@TB-MOF (Fig. 3b). N_2_ adsorption measurement of LiI@TB-MOF after the complete washing with hot MeOH confirmed that the porous structure was well maintained with the BET surface area being estimated to be 729 m^2^ g^−1^ (Fig. 3c). XPS measurements of the I 3d characteristic peaks in LiI@TB-MOF and LiI confirmed the changes of I^−^ ions in LiI@TB-MOF (Fig. S20†). X-ray absorption spectroscopy (XAS) at the Er L_3_ edge was also used to investigate the coordination environment of the Er atoms in LiI@TB-MOF. The extended X-ray absorption fine structure (EXAFS) fitting result showed that LiI@TB-MOF had an additional Er–I shell with an average coordination number of 0.8 ± 0.3 and an average bond length of ∼3.25 Å (Fig. S23 and Table S2†). SEM-EDS mapping showed the uniform distribution of the elements B, Er, and I with no detectable N element from DMF in LiI@TB-MOF (Fig. S24†). FT-IR analysis gave a faint signal associated with DMF at 1679 cm^−1^, confirming that the channel-blocking DMF was almost removed from the erbium cluster (Fig. S25†). The ^7^Li MAS NMR spectrum still showed only one signal at −2.26 ppm, suggesting the uniform chemical environment of Li ions in the highly mobile liquid phase in the frameworks (Fig. 3a). Significantly, the two anionic frameworks, TB-MOF and LiI@TB-MOF, elicited changes in the ^7^Li MAS NMR spectrum, whereas the latter exhibited a little high-field shift to −2.26 ppm relative to TB-MOF. We reasoned that such a shift probably resulted from the change of the anionic channels that became more negative, or there were weak interactions between the Li ions and the I^−^ ions in the resulting framework. These might affect the extranuclear electron cloud of the Li atom, increase the shield effect, and cause ^7^Li resonance in the resulting LiI@TB-MOF shift from −1.0 ppm to −2.26 ppm. ICP-MS analysis provided the content of the Li^+^ increasing to 2.11 wt%, and this content did not increase even when TB-MOF was treated with excess LiI. Significantly, the content of Li^+^ ions would not decrease when LiI@TB-MOF was extensively washed with THF at room temperature or even heating to 80 °C, thus attesting to the strong binding of I^−^ to Er^3+^ ions in the resulting LiI@TB-MOF and ruling out LiI as guest molecules dispersing in the framework. Other lithium salts such as LiCl, LiBr, LiClO_4_, and LiOTf could also be successfully incorporated into TB-MOF to afford LiCl@TB-MOF, LiBr@TB-MOF, LiClO_4_@TB-MOF and LiOTf@TB-MOF, respectively.

To evaluate the improvement of the conductivities, the Li^+^ transference numbers for these LiX (X = Cl^−^, Br^−^, I^−^, ClO_4_^−^, and OTf^−^)-grafted TB-MOFs were first measured. As shown in Table S3,† the Li^+^ transference numbers are 0.89, 0.91, 0.89, 0.80, and 0.74, comparable or even higher than that of pristine TB-MOF 0.70. EIS measurements indicated that these LiX@TB-MOF significantly improved Li-ion conductivities. As shown in Table S3† and Fig. 4d, the room-temperature conductivities were all in the range of (0.26–27.5) × 10^−4^ S cm^−1^, especially for LiI@TB-MOF, which exhibited the highest conductivity of 2.75 × 10^−3^ S cm^−1^, at least two orders of magnitude higher than that of the pristine TB-MOF. Careful inspection further found that the ionic conductivities increased from 1.34 × 10^−4^ to 1.33 × 10^−3^ to 2.75 × 10^−3^ S cm^−1^, with the halide ions changing from Cl to Br to I, and then decreased to 1.51 × 10^−4^ and 2.66 × 10^−5^ S cm^−1^ with the anions switching to ClO_4_ and OTf. This tendency is consistent with the changes in the activation energy, in which LiI@TB-MOF with the highest Li^+^ conductivity displayed the lowest activation energy of 0.15 eV, and LiOTf@TB-MOF with the lowest conductivity possessed the highest activation energy of 0.22 eV. These differences are probably ascribed to the strength of the coordination bond between the anion and the erbium atom that increases with anion softness, thereby decreasing the ion pairing strength between the Li^+^ and anion during the Li^+^ migration.^7,20^ Therefore, both the conductivity and activation energy of our TB-MOF can be systematically tuned by modulating the softness, electronegativity, and size of the grafted anions. For LiOTf@TB-MOF, the conductivity is one magnitude lower than that of lithium halide and perchlorate and even lower than that of the pristine TB-MOF. This result is probably because the size of TfO^−^ is so large that it blocks the anionic channels. Beyond that, we think that the strong electrostatic interactions between the Li^+^ ions and TfO^−^ ions also have an untoward effect on the conductivity. The reappearance of the incomplete semicircle at high frequency provides additional solid evidence to support the existence of bulk resistance in LiOTf@TB-MOF (Fig. 4d).

It is worth noting that our LiI@TB-MOF, which exhibits the highest conductivity at room temperature in our work, surpasses almost all of MOF based Li-ion conductors, including MOF-688 (3.4 × 10^−4^ S cm^−1^),^5^ Cu_4_(ttpm)_2_(CuCl_2_)_0.6_(LiI)_1.0_·20PC (1.1 × 10^−4^ S cm^−1^),^20^ Al-Td-MOF-1 (5.7 × 10^−5^ S cm^−1^),^6^ UiO-66-LiSS (6.0 × 10^−5^ S cm^−1^),^17^ MIT-20-LiBF_4_ (4.8 × 10^−4^ S cm^−1^),^7^ Li/UiOLiTFSI (2.07 × 10^−4^ S cm^−1^),^19^ EHU1(Sc,Li)·(LiBF_4_) (4.2 × 10^−4^ S cm^−1^),^9^ Mg_2_(dobdc)·0.35LiOiPr·0.25LiBF_4_ (2.4 × 10^−4^ S cm^−1^),^4^ LPC@HKUST-1 (3.8 × 10^−4^ S cm^−1^),^10^ LPC@MIL-100-Al (1.0 × 10^−3^ S cm^−1^),^10^ or Li-IL@MOF-525 (3.0 × 10^−4^ S cm^−1^).^12^ To the best of our knowledge, the ambient conductivity of our LiI@TB-MOF is comparable or in the same order of magnitude as the conductivity of some COF based Li-ion conductors such as CD-COF-Li (2.7 × 10^−3^ S cm^−1^),^46^ H-Li-ImCOF (5.3 × 10^−3^ S cm^−1^) and CF_3_-Li-ImCOF (7.2 × 10^−3^ S cm^−1^)^47^ and even exceeds that of some of the well-established ceramic electrolytes, such as glassy Li_2_S–P_2_S_5_ thio-LISICON (1.3 × 10^−3^ S cm^−1^),^48^ Li_6_PS_5_XArgyrodites (1 × 10^−3^ S cm^−1^),^49^ and the Li_10_MP_2_S_12_ family (LiGePS 2.1 × 10^−3^ S cm^−1^ and LiSiPS 2.3 × 10^−3^ S cm^−1^).^12^

The electrochemical performance and the long-term cycling stability among the Li metal anode and LiI@TB-MOF electrolyte were further investigated in a Li/LiI@TB-MOF/Li symmetric cell by the constant current charge–discharge cycle experiment. The measurements were performed at room temperature, and the data during the cycling of lithium plating for 0.5 h and lithium stripping for 0.5 h were collected to simulate realistic cycling conditions. Galvanostatic cycling measurement was conducted at a current density of 0.10 mA cm^−2^. As shown in Fig. 4f, the symmetric cell produced a lower overpotential of less than 20 mV and a relatively stable performance was maintained for more than 300 h without significant fluctuation in polarization potential. Moreover, a flat voltage plateau along the hole charge–discharge cycles was seen from the enlarged voltage profile, reflecting the uniform Li-ion flux directed by LiI@TB-MOF (Fig. S32†). Similar performance was also achieved at an increased current density of 0.20 mA cm^−2^, and the symmetric cell could still cycle stably for 300 h with a polarization voltage as low as 36 mV. Such small and stable potential illustrates that the LiI@TB-MOF electrolyte has good interfacial compatibility with the Li metal electrode. Although a slight fluctuation of overpotential was observed during the initial several cycles, it was gratifying that the cell voltage became relatively stable after the initial 15 cycles.

To gain insight into the symmetric cell during cycling, EIS Nyquist plots of this symmetric cell before, during, and after the constant current charge–discharge cycle experiment were also collected (Fig. S33†). The initial total resistance of the cell was about 930 Ω, and it slightly increased to 1000 Ω after 24 cycles. The resistance was then held around 1200 Ω and remained almost unchanged in the following cycles. Additionally, the symmetric cell was disassembled to investigate the crystallinity integrity of the LiI@TB-MOF electrolyte and the surface morphology evolution of the Li electrode. PXRD showed that the LiI@TB-MOF electrolyte remained highly crystalline after the 300 h Li plating/stripping cycling test (Fig. S34†). The BET surface area of the recycled sample was 718 m^2^ g^−1^ after removing PC (Fig. S35†). SEM analysis of the Li electrode displayed a smooth and integrated surface morphology after the cycling test (Fig. S36†), indicating that the single Li^+^ ion migration in the anionic TB-MOF can avoid the polarization effects and restrain the formation of Li dendrites, usually observed in liquid electrolytes. Finally, a full cell with LiFePO_4_ as a model cathode was fabricated to evaluate the feasibility of TB-MOF in lithium-ion batteries. The full cell delivered an initial specific capacity of 135 mA h g^−1^, and 95% capacity was retained at 0.5 C after 220 cycles with the coulombic efficiency close to 100% (Fig. 4h and S37†). In addition, the full cell also displayed a small discharge charge potential gap of 0.08 V, indicating that the polarization is almost negligible during cycling (Fig. 4i and S38†).

For the role of the PC molecule, we are of the opinion that once the PC solvent is incorporated into TB-MOF, the Li^+^ ions are solvated by PC molecules immediately (Fig. S40†), thereby reducing the electrostatic force between the Li^+^ ions and the anionic framework and increasing the tendency of Li^+^ ions to dissociate from the anionic framework. Moreover, when the anionic channels are filled with PC solvents, the anionic channels can act as highways for the Li^+^ ion rapid transportation. Beyond that, we believe that the PC solvent can wet the surfaces of the MOF particles, facilitating interparticle contacts and making the noumenal Li^+^ ions cross the grain boundaries with great facility. For the Li^+^ ion transportation mechanism, we believe that this is mainly generated from the well-defined anionic channels and abundant negatively charged centers anchored in TB-MOF. Specifically, under the electric field effect, the solvated Li^+^ ions tend to hop from one [BPh_4_]^−^ negatively charged center to the neighbor along the ionic channel, following a minimum hopping mechanism with the shortest distance of 9.60 Å. Once the I^−^ anions are installed into the trierbium clusters, a new negatively charged center is created among the neighbor [BPh_4_]^−^ centers. The shortest distance among the [BPh_4_]^−^ and the resulting trierbium center is 6.71 Å, probably changing the solvated Li^+^ ion hopping from the [BPh_4_]^−^ center to the neighbor trierbium center because the jumping distance decreased by ∼3 Å and ultimately leading to higher ionic conductivity. Additionally, when the crystals are pressed into dense pellets, the opening anionic channels can be interconnected among the crystal particles to form a complete Li^+^ ion conductive network.^43,50^ The highly solvated Li^+^ ions can readily flow from the anionic channels and transfer into the neighbor particles under the electric field to achieve ion migration.

### Conductivity of other metal ions

As a novel and convenient synthetic strategy, solid-state post-synthetic modification has been used to synthesize isomorphous MOFs with different metal ions. As a matter of fact, the anionic framework of TB-MOF provides a chance to provide a driving force for a cation-exchange experiment, in which the free Li^+^ perhaps can be exchanged with other exogenous ions, such as Na^+^, K^+^, Mg^2+^, Ca^2+^, and Zn^2+^ that give the anionic TB-MOF new ion conduction functionalities. In this sense, the vacuum high-temperature activated TB-MOF was first treated with the THF or TEDM solution of various anhydrous metal salts, NaI, KI, MgI_2_, CaI_2_, and ZnI_2_. After that, crystals had to be washed with hot THF and re-immersed into the corresponding new-prepared metal–salt solution to exchange the inherent free Li^+^ ions. This exchange process required at least ten cycles in which the supernatant was poured and replenished with the new solution until almost no free Li^+^ was detected in the supernatant by ICP-MS. These final exchanged samples were collected by filtration, washed with hot THF several times, and then dried under vacuum to afford the corresponding NaI@TB-MOF, KI@TB-MOF, MgI_2_@TB-MOF, CaI_2_@TB-MOF, and ZnI_2_@TB-MOF, respectively. Alternatively, these MI_*x*_@TB-MOFs can also be afforded *via* the solvent-assisted ion exchange of LiI@TB-MOF.

**Fig. 5:**
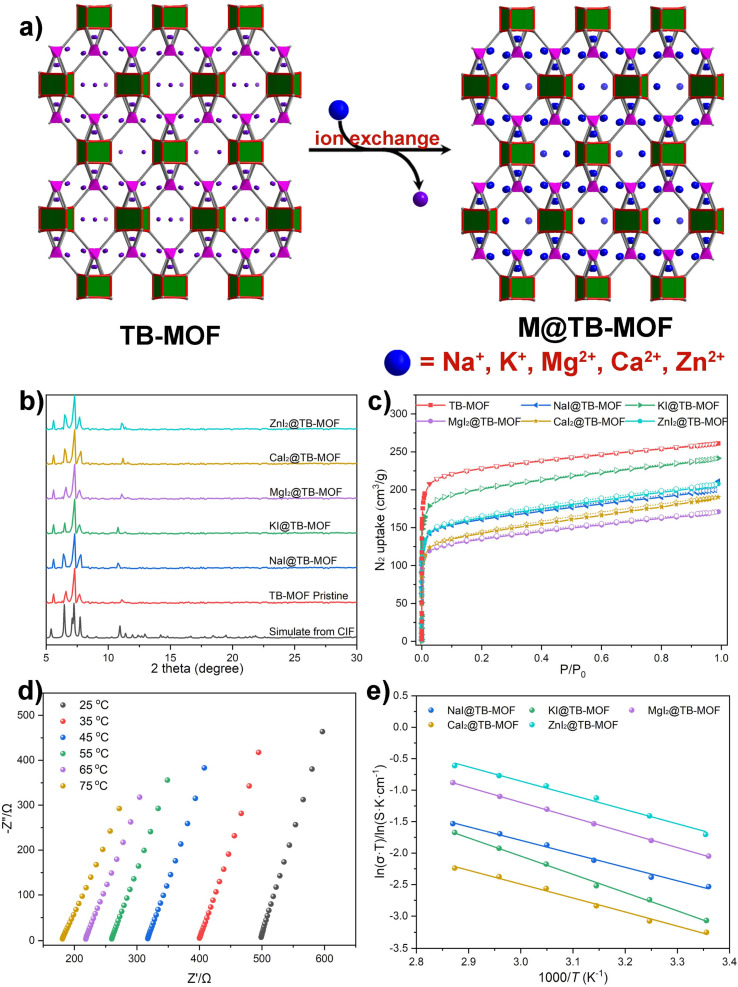
(a) Schematic illustration of the grafting of other metal iodides into TB-MOF. (b) PXRD and (c) N_2_ adsorption isotherms of the resulting MI*x*@TB-MOF. (d) Nyquist plots of MgI_2_@TB-MOF. (e) Arrhenius plots for the ionic conductivity of NaI@TB-MOF, KI@TB-MOF, MgI_2_@TB-MOF, CaI_2_@TB-MOF, and ZnI_2_@TB-MOF.

ICP-MS measurements indicated that the corresponding Na^+^, K^+^, Mg^2+^, Ca^2+^, and Zn^2+^ in the resulting frameworks were about 5.50 wt%, 8.80 wt%, 2.90 wt%, 4.42 wt%, and 7.33 wt%, respectively. PXRD analyses showed that these exchanged samples displayed similar patterns to the pristine TB-MOF (Fig. 5b). N_2_ adsorption experiments provided BET surface areas ranging from 515 to 781 m^2^ g^−1^ (Fig. 5c). SEM-EDS analyses demonstrated the uniform distribution of metal ions throughout the hole crystal (Fig. S53–S57†). In a word, beyond the additional Li^+^ ions that can be incorporated into the anionic framework, other monovalent metal ions such as Na^+^ and K^+^ and even high-value metal ions Mg^2+^, Ca^2+^, and Zn^2+^ can also be successfully inserted into TB-MOF without influence on the crystal structure.

Nyquist plots revealed that the corresponding ion conductivities were evaluated to be 3.09 × 10^−4^ S cm^−1^ for NaI@TB-MOF, 1.64 × 10^−4^ S cm^−1^ for KI@TB-MOF, 3.28 × 10^−4^ S cm^−1^ for MgI_2_@TB-MOF, 1.30 × 10^−4^ S cm^−1^ for CaI_2_@TB-MOF, and 6.10 × 10^−4^ S cm^−1^ for ZnI_2_@TB-MOF, respectively. As shown in Fig. 5d, e, and S40–S44,† the conductivities at varied temperatures followed the Arrhenius law with the activation energies as low as 0.19, 0.25, 0.21, 0.19, and 0.20 eV, for NaI@TB-MOF, KI@TB-MOF, MgI_2_@TB-MOF, CaI_2_@TB-MOF, and ZnI_2_@TB-MOF, respectively. The average ion transference numbers were then calculated to be 0.69, 0.78, 0.76, 0.77, and 0.84, respectively. To the best of our knowledge, the ambient conductivity of our MI_*x*_@TB-MOF is comparable to or in the same order of magnitude as that of some porous materials such as solid-state Na^+^ electrolytes MIL-121/Na + SE (1.2 × 10^−4^ S cm^−1^),^12^ NaOOC-COF (2.8 × 10^−4^ S cm^−1^),^51^ and MIT-20-Na (1.8 × 10^−5^ S cm^−1^),^7^ solid-state K^+^ electrolytes InOF-K (3.76 × 10^−4^ S cm^−1^)^52^ and MOF-808-SO_3_K (3.76 × 10^−4^ S cm^−1^),^53^ solid-state Mg^2+^ electrolytes Mg-MOF-74 ⊃ {Mg(TFSI)_2_}_0.15_ (2.6 × 10^−4^ S cm^−1^),^16^ MIL-101 ⊃ {Mg(TFSI)_2_}_1.6_ (19 × 10^−4^ S cm^−1^),^11^ and MOF-MgBr_2_ (1.3 × 10^−4^ S cm^−1^),^20^ and solid-state Zn^2+^ electrolytes Zn_3_[(Zn_4_Cl)_3_(BTT)_8_]_2_ (1.15 × 10^−4^ S cm^−1^),^31^ TpPa-SO_3_Zn_0.5_ (2.2 × 10^−4^ S cm^−1^),^54^ and Zn@MOF-808 (2.1 × 10^−4^ S cm^−1^).^55^ Fig. S47† displays the voltage profiles of the galvanostatic charge–discharge of the Mg/MgI_2_@TB-MOF/Mg symmetric cell. This cell achieved stable cyclability over 100 hours at room temperature, highlighting the potential of MgI_2_@TB-MOF as a solid electrolyte for solid-state magnesium batteries.

## Conclusions

In summary, we have constructed an anionic metal–organic framework TB-MOF by employing a carboxylic acid functionalized lithium tetraphenylborate ligand. The Li^+^ counterions were directly encapsulated into the anionic MOF to become the only free mobile charge carrier that produced a lithium-ion solid electrolyte with ionic conductivity up to 6.42 × 10^−5^ S cm^−1^ at 35 °C. After grafting LiI into the metal clusters to replace the channel-blocking DMF molecules, the ionic conductivity reached a record of 2.75 × 10^−3^ S cm^−1^ at 25 °C. Benefitting from the high mobility of the charge carrier, the free Li^+^ could exchange with other exogenous ions, such as Na^+^, K^+^, Mg^2+^, Ca^2+^, and Zn^2+^, endowing the anionic TB-MOF with new ion conduction functionalities. Our work realized the directional preparation of an anionic metal–organic framework by utilizing the anionic ligand as the source. The further exploration of the channel structure–conductivity relationship effectively regulated the ionic conductivities, extending the application of anionic MOFs into lithium and other metal solid-state electrolytes. This work has not only provided new approaches and technology for building SSEs with high ionic conductivity but also demonstrated fundamental significance and potential application prospects for developing new kinds of solid ionic conductors.

## Author contributions

Q. X. and K. H. conceived and designed the experiments. K. H. and X. M. performed the experiments and analyzed the data. Q. X. and Z. L. carried out single-crystal X-ray analyses. X. M. and P. Q. fabricated the full cell. Q. X. and X. C. wrote the paper. All authors discussed the results and commented on the manuscript.

## Conflicts of interest

The authors declare no competing interests.

## Supplementary Material

SC-015-D4SC02861A-s001

SC-015-D4SC02861A-s002

## Data Availability

All experimental procedures and ESI tables and figures are available in the ESI.†
