# Supplementary material for: A tetraphenylborate-based anionic metal–organic framework as a versatile solid electrolyte for fast Li+, Na+, K+, Mg2+, Ca2+, and Zn2+ transportation
Source: Chem Sci. 2024 Oct 7;15(42):17579–89. doi: 10.1039/d4sc02861a (PMC11457371; doi:10.1039/d4sc02861a)

Supplementary Information (SI) for Chemical Science.
This journal is © The Royal Society of Chemistry 2024

Supporting Information for

Tetraphenylborate-based Anionic Metal−Organic Framework as a Versatile Solid Electrolyte for Fast Li^+, Na^+, K^+, Mg^{2+}, Ca^{2+}, and Zn^{2+} Transportation

Qingchun Xia,*,† Kaixin Han,† Xuxiao Ma,† Pengtao Qiu,*,† Zhiyong Li,† and Xuenian Chen*,†,‡

†*Henan Key Laboratory of Boron Chemistry and Advanced Energy Materials, Collaborative Innovation Center of Henan Province for Green Manufacturing of Fine Chemicals, Key Laboratory of Green Chemical Media and Reactions, Ministry of Education, School of Chemistry and Chemical Engineering, Henan Normal University, Xinxiang, Henan 453007, China.*

‡ *College of Chemistry, Zhengzhou University, Zhengzhou, Henan 450001, China.*

Email: xiaqingchun@htu.edu.cn; qiupengtao@htu.edu.cn; xuenian_chen@zzu.edu.cn

Table of Content

1. Materials and general procedures S3
2. Experimental S3
3. Electrochemical measurements S6
4. Crystal data and structure refinement for TB-MOF and TC-MOF S9
5. Additional X-ray crystallographic structures S9
6. ^{7}Li MAS NMR spectra S12
7. TGA of TB-MOF S13
8. ^{11}B MAS NMR, ^{11}B NMR, and ^{13}C NMR S13
9. PXRD patterns of TC-MOF S15
10. BET surface area of TC-MOF S16
11. Electronic and ionic conductivities S16
12. XPS and XAFS spectroscopy S19
13. SEM and SEM-EDS mapping of LiI@TB-MOF S22
14. IR spectrum of TB-MOF S23
15. TGA cure of LiX@TB-MOF and M@TB-MOF S23
16. Ionic conductivity and Li^+ transference number of LiX@TB-MOF S23
17. The interfacial compatibility and the SSB performance test S26
18. ^{7}Li NMR of lithium tetrakis(4-iodophenyl)borate S29
19. Size comparison between nanocage TB-MOF and PC molecule S29
20. Ionic conductivity and transference number of M@TB-MOF S30
21. The galvanostatically cycle of Mg@TB-MOF S32
22. The micrographs of TB-MOF, and its scale-up synthesis. S33
23. IR spectrum S42

1. Materials and general procedures

All reagents and solvents used in these studies are commercially available and used without further purification.

Single-crystal XRD data for TB-MOF and TC-MOF were collected on a Bruker SMART Apex II CCD-based X-ray diffractometer with Cu-Kα radiation (λ = 1.54178 Å) at 150 K. The empirical absorption correction was applied by using the SADABS program (G. M. Sheldrick, SADABS, program for empirical absorption correction of area detector data; University of Göttingen, Göttingen, Germany, 1996). The structure was solved by direct methods with SHELXS-2018 and refined with SHELXL-2018 using OLEX 1.2. In the structure, all the non-hydrogen atoms were refined by full-matrix least-squares techniques with anisotropic displacement parameters, and the hydrogen atoms were geometrically fixed at the calculated positions attached to their parent atoms, treated as riding atoms. Contributions to scattering due to these highly disordered solvent molecules except two DMF molecules were removed using the *SQUEEZE* routine of *PLATON*; structures were then refined again using the data generated. Among these data sets for TC-MOF, we chose the best one for structure solution and refinement, but there were still two B level alert problems that the H_2O molecules do not form hydrogen bonds in checkcif, that maybe due to the absence of hydrogen bonding interactions between the H_2O molecule and the solvent molecules.

The structure was then refined again using the data generated. Crystal data and details of the data collection are given in **Table S1**, while the selected bond distances and angles are presented in **Table S2**. CCDC 2222633 and 2222634 contains the supplementary crystallographic data for this paper. These data can be obtained free of charge from the Cambridge Crystallographic Data Centre via www.ccdc.cam.ac.uk/data request/cif

2. Experimental

2.1 Synthesis of Li·[H_4L] ligand

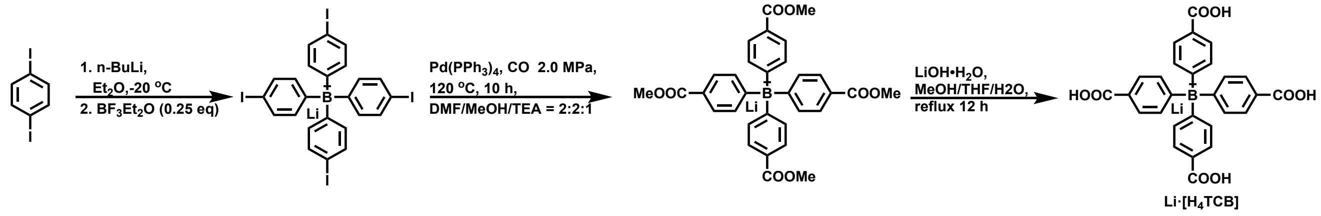

2.1.1 Synthesis of Lithium Tetrakis(4-iodophenyl)borate

1,4-Diiodobenzene (32.9 g, 100 mmol) was dissolved in Et_2O (150 mL). The solution was cooled to −20 °C and treated dropwise with a solution of n-BuLi (40 mL, 2.5 M in hexane, 100 mmol) while maintaining the temperature below −20 °C. The resulting mixture was kept at −10 °C for 30 min, and $BF_3 \cdot Et_2O$ (2.3 ml, 18.7 mmol) was then added. After that, the cooling bath was removed and the mixture was allowed to warm to room temperature and left overnight. The mixture was filtered to get a solid, and the solid was washed with ether several times, which was then redissolved into the acetone (100 mL). Filter and remove the insoluble LiF, the filtrate was concentrated under reduced pressure to remove the acetone, leaving the desired lithium tetrakis(4-iodophenyl)borate 14.9 g with 72% yield. ^{1}H NMR (600 MHz, DMSO-d_6): δ 6.93-6.90 (m, 8H), 7.31 (d, 8H, J = 7.8 Hz). ^{11}B NMR (193 MHz, DMSO-d_6): δ −7.31. ^{13}C NMR (151 MHz, DMSO-d_6): δ 88.3, 134.5, 137.9, 161.7 (m).

2.1.2 Synthesis of Lithium Tetrakis (4-(methoxycarbonyl) phenyl) borate

To a mixture of DMF (52 mL), MeOH (52 mL) and triethylamine (26 mL) in an autoclave, lithium tetrakis (4-iodophenyl) borate (13.0 g, 15.37 mmol) and $Pd(PPh_3)_4$ (3.3 g, 3.0 mmol, 20 mol%) were added. Seal the autoclave and press the CO to 2 MPa, the autoclave was stirred and heated at 120 ^oC for 10 hours. After cooling to room temperature, the solvents were removed under reduce pressure, and the residue was redissolved into ethyl acetate. The mixture was then filtered to get the filtrate, and the filtrate was concentrated under vacuum and then purified by column chromatography on silica gel (EtOAc/ Actione, 10/1, v/v) to afford the desired product lithium tetrakis (4-(methoxycarbonyl) phenyl) borate 6.43 g with 75% yield. ^1H NMR (600 MHz, DMSO-d_6): δ = 7.62 (d, J = 7.8 Hz, 8H), 7.27 (m, 8H), 3.78 (s, 12H). ^{11}B NMR (193 MHz, DMSO-d_6): δ = −6.37. ^{13}C NMR (151 MHz, DMSO-d_6): 169.83 (m), 167.39, 135.24, 126.84, 124.13, 51.49.

2.1.3 Synthesis of Lithium tetrakis(4-carboxyphenyl)borate (Li·[H$_4$L])

To a mixture of MeOH (40 mL), THF (40 mL) and water (40 mL) in a round-bottom flask, lithium tetrakis (4-(methoxycarbonyl) phenyl) borate (4.1 g, 7.14 mmol), $LiOH\cdot H_2O$ (1.43g, 34.08 mmol) were added. The mixture was refluxed for 16 h. MeOH and THF were removed by evaporation, and the residue was diluted with H_2O and washed with EA for 3 times. The aqueous phase was acidified with concentrated HCl, and the precipitate was collected by filtration and then dried under vacuum to afford 2.32 g of brown solid Li·[H$_4$**L**] with 65% yield. ^1H NMR (600 MHz, DMSO-d_6): δ = 7.59 (d, J = 8.1 Hz, 8H), 7.25 (m, 8H). ^{11}B NMR (193 MHz, DMSO-d_6): δ = −6.43. ^{13}C NMR (151 MHz, DMSO-d_6):169.37 (m), 168.68, 135.19, 127.09, 125.21.

2.2 Synthesis of TB-MOF

To a mixture of DMF (5mL) and MeOH (10mL) in a vial, $ErCl_3\cdot 6H_2O$ (0.2 mmol), lithium tetrakis(4-carboxyphenyl)borate (Li·[H$_4$L]) (0.25 mmol) and HCOOH (1 mL) were added. The reaction solution was sealed with a screw cap and heated at 80 ^oC for 48 h. After that, the colorless crystals were collected, washed with MeOH, and dried under vacuum at room temperature to afford the TB-MOF (32 mg, 63%). For the ICP-MS measurements, 5.000 mg TB-MOF was digested with 10 mL aqua regia in a small beaker, then diluted by 10 wt% HNO_3 and transferred to a volumetric flask, washed the beaker three times, and then diluted with dilute nitric acid to 100 mL for ICP measurement. Other MX@TB-MOF was digested in the same manner.

The TB-MOF can be best formulated as $Li_2\cdot[Er_3(\mathbf{L})_2(HCOO)(DMF)_2(H_2O)]\cdot nG$ on the basis of EA, TGA, IR, ICP-MS and single-crystal diffraction. Elemental analysis for TB-MOF: Anal (%). Calcd for $C_{63}H_{49}B_2Er_3O_{21}N_2Li_2$; C, 44.32; H, 2.89; N, 1.64. Found: C, 45.48; H, 2.34; N, 1.55. The calculated molar ratio of Li:B:Er is 1:1:1.5, and ICP measurement was found to be 1.12:0.93:1.5. IR (KBr, cm^{-1}): 2939(w), 1655 (s), 1587 (m), 1571 (m), 1493 (m), 1390 (s), 1244 (w), 1180 (w), 1098 (m), 1058 (w), 1018 (m), 859 (w), 831 (m), 771 (s), 720 (w), 680 (m), 665 (m), 565 (w).

2.3 Synthesis of TC-MOF

The synthesis of TC-MOF was the same as TB-MOF except that the ligand was exchanged with tetrakis(4-carboxyphenyl)methane (H_4TCM). The product can be best formulated as $[Er_3(TCM)_2(HCOO)(DMF)_2(H_2O)]\cdot nG$ on the basis of EA, TGA, IR and single-crystal diffraction. Elemental analysis for TC-MOF: Anal (%). Calcd for $C_{65}H_{49}Er_3N_2O_{21}$; C, 46.04; H, 2.91; N, 1.65. Found: C, 45.33; H, 2.44; N, 1.27. IR (KBr, cm^{-1}): 1663 (m), 1633 (m), 1600 (m), 1536 (m), 1412 (s),

1256 (w), 1190(w), 1111 (w), 1016 (m), 857 (m), 778 (s), 720 (w), 675 (m).

2.4 Synthesis of LiX@TB-MOF *via* post-synthesis modification

To a dried Schlenk tube, 60 mg TB-MOF was added. The Schlenk tube was then immersed into the oil bath and heated at 120 °C under dynamic vacuum at least for 12 h. After cooling to room temperature, the Schlenk tube was refilled with argon and then added 10 mL THF saturated solution of LiCl. The crystals were immersed in the solution for 3 days to make sure that all the coordinatively unsaturated Er^{3+} sites were decorated with Cl^{-}. The crystals were then collected by vacuum filtration, washed with THF several times, and dried at 80 °C under vacuum to afford almost equal yield LiCl@TB-MOF as a white free-flowing powder. Other LiX@TB-MOF, including LiBr@TB-MOF, LiI@TB-MOF, $LiClO_4$@TB-MOF, and LiOTf@TB-MOF, were synthesized the same as LiCl@TB-MOF, except that the lithium salt was changed to LiBr, LiI, $LiClO_4$, and LiOTf, respectively.

Elemental analysis for LiCl@TB-MOF: Anal (%). Calcd for $C_{57}H_{17}B_2Cl_3O_{18}Er_3Li_5$; C, 41.39; H, 1.04. Found: C, 42.28; H, 1.79. The calculated molar ratio of Li:B:Er is 2.5:1:1.5, and the ICP measurement was found to be 2.71:1.14:1.5. IR (KBr, cm^{-1}): 2997 (w), 2935 (w), 1790 (s), 1657 (w), 1595 (w), 1512 (m), 1491 (m), 1390 (s), 1351 (m), 1175 (s), 1118 (m), 1050 (m), 1018 (w), 859 (m), 834 (m), 769 (m), 712 (s), 681 (m), 672 (m), 556 (w), 549 (w), 425 (w).

Elemental analysis for LiBr@TB-MOF: Anal (%). Calcd for $C_{57}H_{17}B_2Br_3O_{18}Er_3Li_5$; C, 38.30; H, 0.96. Found: C, 39.38; H, 1.06. The calculated molar ratio of Li:B:Er is 2.5:1:1.5, and the ICP measurement was found to be 2.63:0.83:1.5. IR (KBr, cm^{-1}): 2989 (w), 2932 (w), 1785 (s), 1657 (w), 1592 (w), 1518 (m), 1390 (s), 1180 (m), 1115 (w), 1047 (m), 857 (w), 774 (m), 717 (s), 681 (m), 672 (m), 553 (w), 431 (w).

Elemental analysis for LiI@TB-MOF: Anal (%). Calcd for $C_{57}H_{17}B_2I_3O_{18}Er_3Li_5$; C, 35.50; H, 0.89. Found: C, 36.03; H, 1.10. The calculated molar ratio of Li:B:Er is 2.5:1:1.5, and the ICP measurement was found to be 2.37:0.91:1.5. IR (KBr, cm^{-1}): 2986 (w), 2932 (w), 1791 (s), 1666 (w), 1592 (w), 1518(m), 1385 (s), 1180(m), 1118 (w), 1053 (s), 857(m), 774 (m), 683 (m), 666 (m), 550 (w), 419 (w).

Elemental analysis for $LiClO_4$@TB-MOF: Anal (%). Calcd for $C_{57}H_{17}B_2Cl_3O_{30}Er_3Li_5$; C, 37.08; H, 0.93. Found: C, 36.12; H, 0.89. The calculated molar ratio of Li:B:Er is 2.5:1:1.5, and the ICP measurement was found to be 2.74:1.21:1.5. IR (KBr, cm^{-1}): 2992 (w), 2935 (w), 1788 (s), 1660 (w), 1596 (w), 1512 (m), 1388 (s), 1348 (m), 1320 (w), 1180 (s), 1118 (w), 1075 (w), 1044 (m), 1018 (w), 857 (m), 837 (m), 769 (m), 717 (s), 683 (m), 66 (m), 553 (m), 479 (w), 424 (w).

Elemental analysis for LiOTf@TB-MOF: Anal (%). Calcd for $C_{60}H_{17}B_2F_9S_3O_{27}Er_3Li_5$; C, 36.12; H, 0.86. Found: C, 35.43; H, 1.33. The calculated molar ratio of Li:B:Er is 2.5:1:1.5, and the ICP measurement was found to be 2.85:1.12:1.5. ICP measurement indicated the molar ratio of Li:B:Er is about 2.5:1:1.5. IR (KBr, cm^{-1}): 2992 (w), 2932 (w), 1788(s), 1663 (w), 1598 (w), 1518 (m), 1390 (s), 1308 (w), 1254 (w), 1180(m), 1118 (w), 1047 (m), 854 (m), 774 (m), 717 (s), 681 (m), 666(m), 556 (w), 442 (w).

2.5 Synthesis of NaI@TB-MOF, KI@TB-MOF, MgI_2@TB-MOF, CaI_2@TB-MOF, and

ZnI_2@TB-MOF *via* post-synthesis exchange

To a dried Schlenk tube, 60 mg TB-MOF was added. The Schlenk tube was then immersed into the oil bath and heated at 120 °C under a dynamic vacuum for at least 12 h. After cooling to room temperature, the Schlenk tube was refilled with argon and then added 10 mL THF saturated solution of NaI. The crystals were immersed in this solution for 24 h to make sure that all the coordinatively unsaturated Er^{3+} sites were decorated with I^-. After that, the crystals were collected by vacuum filtration, washed with hot THF (10 mL × 5) solvent and then re-immersed into 10 mL fresh-prepared NaI solution at room temperature for another 24 h. The synthesis of NaI@TB-MOF required at least ten exchange cycles during which the NaI solution was decanted and freshly replenished at least ten times until no free Li^+ was detected by ICP-MS. The final product was collected by vacuum filtration, washed with THF, and dried at 80 °C under vacuum to afford NaI@TB-MOF as a white free-flowing powder. Other MI*x*@TB-MOF including KI@TB-MOF, MgI_2@TB-MOF, CaI_2@TB-MOF, and ZnI_2@TB-MOF were synthesized the same as NaI@TB-MOF except that the THF saturated solution of NaI was exchanged with triglyme saturated solution of KI, triglyme saturated solution of MgI_2, THF saturated solution of CaI_2, and THF saturated solution of ZnI_2.

Elemental analysis for NaI@TB-MOF: Anal (%). Calcd for $C_{57}H_{17}B_2Er_3O_{18}Na_5I_3$; C, 34.08; H, 0.85. Found: C, 34.33; H, 0.76. The calculated molar ratio of Na:B:Er is 2.5:1:1.5, and the ICP measurement was found to be 2.77:1.19:1.5. IR (KBr, cm^{-1}): 2989 (w), 2941 (w), 1796 (s), 1656 (w), 1598 (w), 1524 (m), 1486 (m), 1385 (s), 1304 (w), 1252 (w), 1183 (m), 1145 (w), 1123 (w), 1115 (w), 1073 (w), 1047 (w), 1018 (w), 853 (m), 769 (m), 712 (s), 683(m), 672 (m), 554(w), 423 (w).

Elemental analysis for KI@TB-MOF: Anal (%). Calcd for $C_{57}H_{17}B_2Er_3O_{18}K_5I_3$; C, 32.77; H, 0.82. Found: C, 32.91; H, 0.63. The calculated molar ratio of K:B:Er is 2.5:1:1.5, and the ICP measurement was found to be 2.24:0.86:1.5. IR (KBr, cm^{-1}): 2989(w), 2941 (w), 1791 (s), 1661 (w), 1598 (w), 1521 (m), 1489 (m), 1396 (s), 1349 (m), 1306 (w), 1229 (w), 1189 (m), 1144 (w), 1123 (w), 1055 (m), 1021 (w), 859 (m), 771 (m), 715 (s), 686 (m), 669 (m), 553 (w), 482 (w), 425 (w).

Elemental analysis for MgI_2@TB-MOF: Anal (%). Calcd for $C_{57}H_{17}B_2Er_3O_{18}Mg_{2.5}I_3$; C, 35.03; H, 0.88. Found: C, 34.599; H, 1.05. The calculated molar ratio of Mg:B:Er is 1.25:1:1.5, and the ICP measurement was found to be 1.43:1.07:1.5. IR (KBr, cm^{-1}): 2994 (w), 2941 (w), 1788 (s), 1596 (w), 1524 (w), 1388 (s), 1353 (w), 1306 (w), 1180 (m), 1115 (w), 1053 (s), 1021 (w), 933 (w), 859 (m), 777 (m), 717 (s), 683 (w), 666 (w), 550 (w), 476 (w), 417(w).

Elemental analysis for CaI_2@TB-MOF: Anal (%). Calcd for $C_{57}H_{17}B_2Er_3O_{18}Ca_{2.5}I_3$; C, 34.33; H, 0.86. Found: C, 35.12; H, 0.67. The calculated molar ratio of Ca:B:Er is 1.25:1:1.5, and the ICP measurement was found to be 1.12:0.81:1.5. IR (KBr, cm^{-1}): 2992 (w), 2932 (w), 1791 (s), 1654 (w), 1592 (m), 1573 (m), 1490 (m), 1393 (s), 1349 (m), 1186 (m), 1115 (m), 1047 (m), 1014 (m), 949 (w), 862 (w), 831 (w), 769 (s), 717 (m), 678 (w), 638 (w), 513 (w), 488 (w), 422 (w).

Elemental analysis for ZnI_2@TB-MOF: Anal (%). Calcd for $C_{57}H_{17}B_2Er_3O_{18}Zn_{2.5}I_3$; C, 33.28; H, 0.83. Found: C, 32.99; H, 0.63. The calculated molar ratio of Zn:B:Er is 1.25:1:1.5, and the ICP measurement was found to be 1.53:1.24:1.5. IR (KBr, cm^{-1}): 2989 (w), 2935 (w), 1785 (s), 1660 (w), 1586 (m), 1495 (m), 1390 (s), 1353 (w), 1177 (s), 1123 (m), 1047 (s), 1014 (m), 859(w), 834 (w), 766

(s), 709 (m), 678 (w), 629 (w), 519 (w), 488 (w), 419 (w).

3. Electrochemical measurements

3.1 Ionic conductivity

After the pristine TB-MOF provided by solvothermal reactions, they were then washed with MeOH, dried under vacuum at room temperature, immersed in propylene carbonate (PC) solvent for 24 h. After that, the white powder TB-MOF was collected by filtration or centrifugation, simply washed with THF, and then dried under vacuum to afford the free-flowing dry powder.

In an Ar glovebox, 50 mg TB-MOF was pressed into a pellet under at 5 Mpa force, ranged from 1.0 to 1.4 mm and assembled into an airtight Swagelok-type cell between stainless steel blocking electrodes for the ionic conductivity (SS|TB-MOF|SS).

The alternating current (AC) impedance analysis was performed using a two-probe method with a Autolab AUT88031 potentiostat/galvanostat over the frequency range 10^{-2} to 10^{6} Hz, with an input voltage amplitude of 100 mV. The test cell was maintained at the target temperature by an BXH-655 precise programmable oven. Data was recorded every 5 °C between 25 to 85 °C with heating-cooling-heating. Each temperature was held for 1.5 h to reach thermal equilibrium. After each cycle, the temperature was maintained for 3 h before moving on to the subsequent cycle. At each temperature point, the ionic conductivities (σ) were determined according to following equation:

$$\sigma = \frac{L}{AR} \qquad (1)$$

where L and S represent the thickness (cm) and the area (cm^2) of the pellet, respectively. And R, which was extracted directly from the impedance plots, is the bulk resistance of the sample (Ω). The activation energy (E_a) was calculated with the Nernst-Einstein relation:

$$\sigma = \frac{\sigma_0}{T} exp(-\frac{E_a}{kT}) \qquad (2)$$

where σ_0 is a pre-exponential factor, T is the temperature, E_a is the activation energy and k is the Boltzmann constant.

3.2 Ionic transference number

Ionic transference number was evaluated using a potentiostatic polarization method at room temperature. The powder pellet was prepared in an airtight Swagelok-type cell by pressing pellet between Li foil of 6.35 mm diameter under dry Ar atmosphere. Pressure was applied by tightening the spring tension adjustment nut on the upper split. The direct current (DC) polarization measurement was performed on the symmetric Li|sample|Li cells using a Autolab AUT88031 potentiostat/galvanostat with an applied polarized voltage, ranged from 10 mV to 50 mV. The AC impedance of the symmetric cell was conducted over the frequency range 1 MHz -1 Hz, with an input voltage amplitude of 100 mV. The ionic transference number t was determined according to following equation:

$$t = \frac{\mathrm{IS(\Delta V - I0R0)}}{I0(\Delta V - ISRS)} \qquad (3)$$

where I^S is the steady-state current, I^0 is the initial current, ΔV is the applied potential, R^0 and R^S

are the interfacial resistances before and after the polarization, respectively.

The Na, K, and Ca foil was prepared by the following method: Typically, in the argon glove box, the kerosene on the surface of the Na-metal was wiped off by filter paper, washed the Na with n-hexane, and dried. About one gram of Na-metal was cut, pressed into the foil with a jack, trimmed with scissors, and carefully polished with sandpaper until the sodium foil exhibited a silver-white metallic luster. The Ca foil was prepared using the same method. For the potassium foil, about one gram of metal potassium was cut and then rolled to the potassium foil with a stainless steel SUS304 round rod. Their relevant ionic transference numbers were measured to be the same as that of Li^+.

3.3 Linear sweep voltammograms (LSV) experiment

Linear sweep voltammetry (LSV) was conducted with a SS|TB-MOF|Li asymmetric cell operated under a sweep rate of 5 mV/s in a voltage range from 0 to 6.0 V (vs. Li/Li^+) at room temperature.

3.4 Cyclic voltammetry (CV) measurement

Cyclic voltammetry (CV) studies were performed with the SS|TB-MOF|Li asymmetric cell at a scan rate of 5 mV/s with the voltage range from -0.5 to 5.0 V (vs. Li/Li^+) at room temperature.

3.5 Galvanostatic cycling measurements

Galvanostatic cycling measurements were performed on the same symmetrical cell setup used for transference number measurements. Galvanostatic cycling for symmetrical cells Li|TB-MOF|Li was performed using a multichannel battery testing system (LAND CT3001A) by sequentially charging/discharging for 30 min at a current density of 0.1mA cm^{-2} or 0.2mA cm^{-2}.

3.6 Galvanostatic charge/discharge measurements on Coin Cell Battery

Galvanostatic charge/discharge tests of the Li|LiI@TB-MOF|$LiFePO_4$ cell were recorded by cycling between 2.50 to 4.00 V vs Li^+/Li with a current density of 0.5 C (1 C = 170 mA g^{-1}, based on the theoretical capacity of $LiFePO_4$) after a 12 h rest at 30 °C. The coin cell was measured by a LAND CT3001A instrument.

The $LiFePO_4$ cathode was prepared from a slurry of 75 wt% $LiFePO_4$, 15 wt% acetylene black, and 10 wt% PVDF in NMP. The slurry was ground and then coated on an Al foil carefully. The coated Al foil was dried at 85 °C under vacuum for 12 h, and pressed for 2 min under a pressure of 30 MPa. The mass loading of $LiFePO_4$ is about 2~4 mg cm^{-2}. Before assembled into a CR2032 coin cell, the Li anode was soaked in the pure solvent of fluoroethylene carbonate (FEC) for 5 h to improve the electrochemical stability.

3.7 DC measurements of the electrical conductivity

The electrical conductivity, σ, is generally obtained by fitting the linear region of a current−voltage (I−V) curve to Ohm's law. From the Ohm's law:

$$R = \frac{V}{I}$$

For the TB-MOF,

$$R = \frac{L}{\sigma A}$$

$A = \pi r^2$

L and r are the thickness and radius of the pellet, respectively.

Therefore,

$$\sigma = \frac{L}{\pi r^2} \times \frac{I}{V}$$

The activated TB-MOF was ground into a fine powder in an Ar glovebox and then pressed into a thin pellet using a KBr die. The diameter and thickness of the pellet were measured with a vernier caliper, and then the pellet was sealed in an airtight Swagelok-type cell to assemble a symmetric Cu/TB-MOF/Cu cell. A direct current (DC) voltage ranging from −0.8 to 0.8 V was then applied to the symmetric Cu/TB-MOF/Cu cell (Figure S15), and their corresponding currents were measured to draw the I−V curve. The slope of the fitted lines was then calculated to determine the electrical conductivity σ using the above formula.

4. Table S1. Crystal data and structure refinement for TB-MOF and TC-MOF.

Identification code	TB-MOF	TC-MOF
Empirical formula	$C_{63}H_{49}B_2Er_3N_2O_{11}$	$C_{65}H_{49}Er_3N_2O_{11}$
Formula weight	1533.49	1695.84
Temperature (K)	170.00	150.00
Wavelength (Å)	CuKα (λ = 1.54184)	CuKα (λ = 1.54184)
Crystal system	orthorhombic	orthorhombic
Space group	*P*nma	*P*nma
Unit cell dimensions	a = 32.6786(5) Å<br>b = 24.8964(4) Å<br>c = 13.9814(3) Å<br>$\alpha = \beta = \gamma = 90$	a = 32.1662(7) Å<br>b = 24.9817(4) Å<br>c = 13.4105(5) Å<br>$\alpha = \beta = \gamma = 90$
Volume (Å^3), Z	11375.0(4), 8	10776.2(5), 4
Density (calculated) (mg/m^3)	0.988	1.045
Absorption coefficient (mm^{-1})	4.312	4.555
F(000)	3284	3300.0
θ range for data collection (°)	6.876 to 142.59	7.482 to 145.204
Limiting indices	$-19 \leq h \leq 39$<br>$-26 \leq k \leq 30$<br>$-17 \leq l \leq 14$	$-39 \leq h \leq 34$<br>$-21 \leq k \leq 30$<br>$-16 \leq l \leq 16$
Reflections collected	25680	34576
Independent reflections	11018<br>[R_{int} = 0.0348, R_{sigma} = 0.0501]	10616<br>[R_{int} = 0.0305, R_{sigma} = 0.0282]
Completeness to theta	99.4%	99.5%
Data / restraints / parameters	11018/115/470	10616/116/470
Goodness-of-fit on F^2	1.101	1.032
Final R indices [I>2sigma(I)]	R_1 = 0.0537, wR_2 = 0.1479	R_1 = 0.0502, wR_2 = 0.1287
R indices (all data)	R_1 = 0.0687, wR_2 = 0.1537	R_1 = 0.0660, wR_2 = 0.1449
Largest diff. peak and hole (e.Å^{-3})	1.21/-1.05	1.25/-1.24

5. Figure S1~S8. Additional X-ray crystallographic structures

5.1 Figure S1. The asymmetric unit in TB-MOF

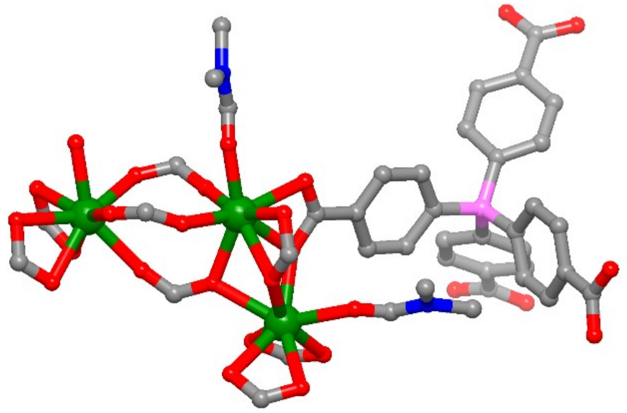

5.2 Figure S2. The coordination environments of the Er_3 cluster

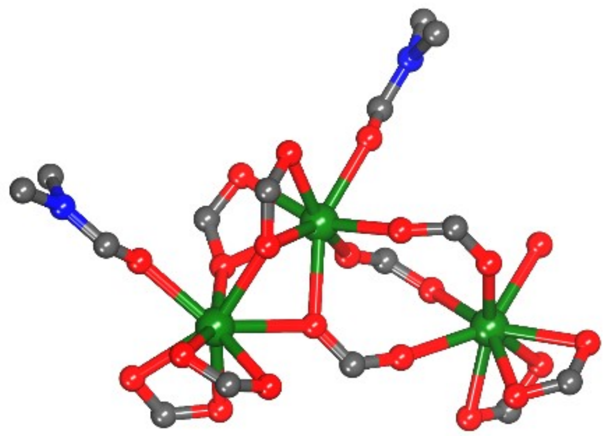

5.3 Figure S3. The distance of the negative charge centers BPh_4 in TB-MOF

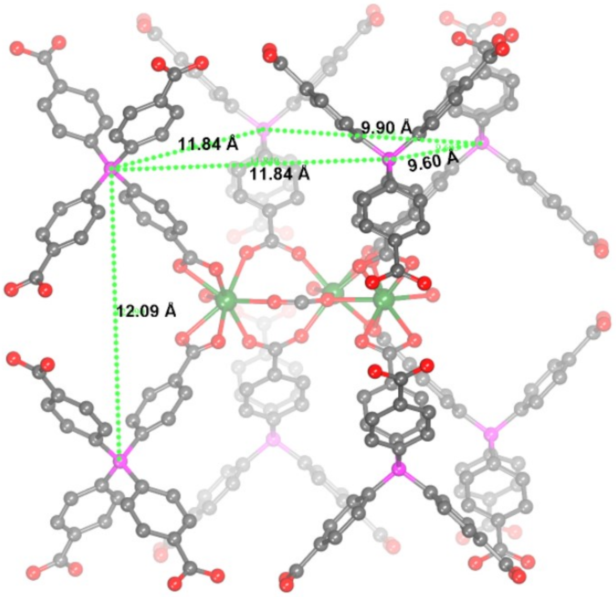

5.4 Figure S4. The packing mode and simplified dodecahedral cage in TB-MOF

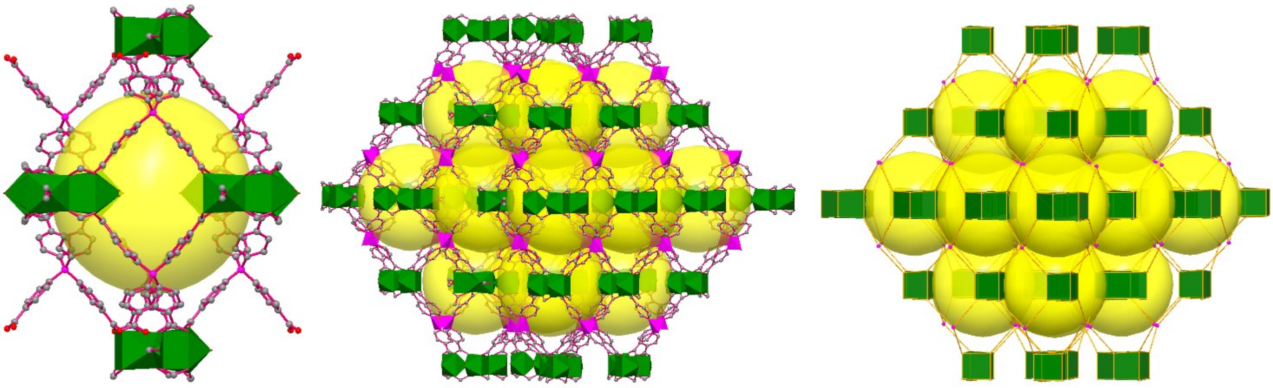

5.5 Figure S5. The simplification and the space filling of the 3D porous structure of TB-MOF

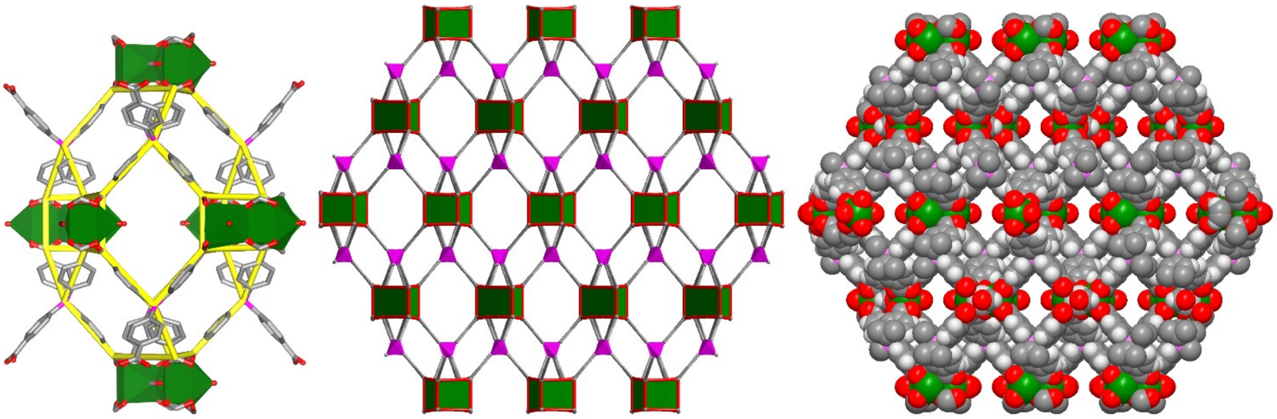

5.6 Figure S6. The simplification of the distorted dodecahedral cage

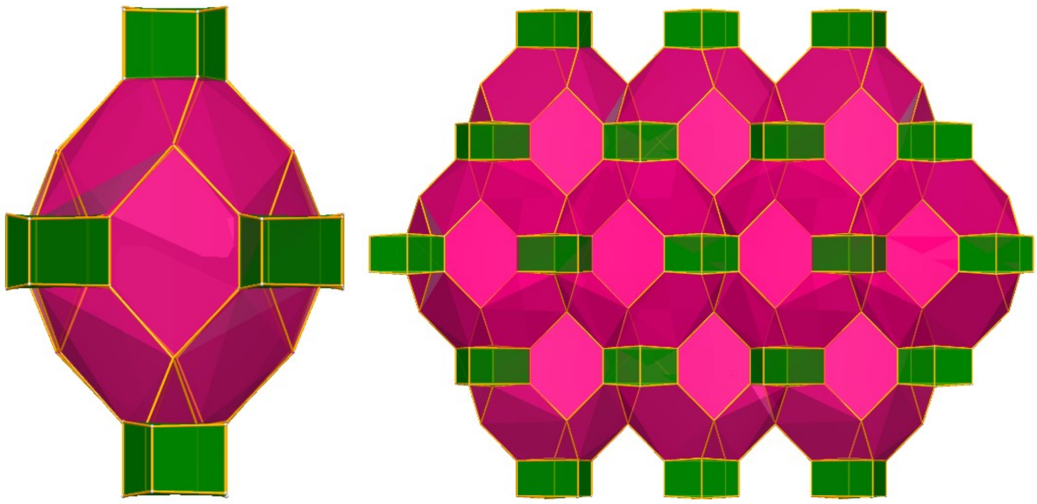

5.7 Figure S7. The ionic channels alone in *a* (left) and *c* (right) axes blocked by the coordinated DMF molecules

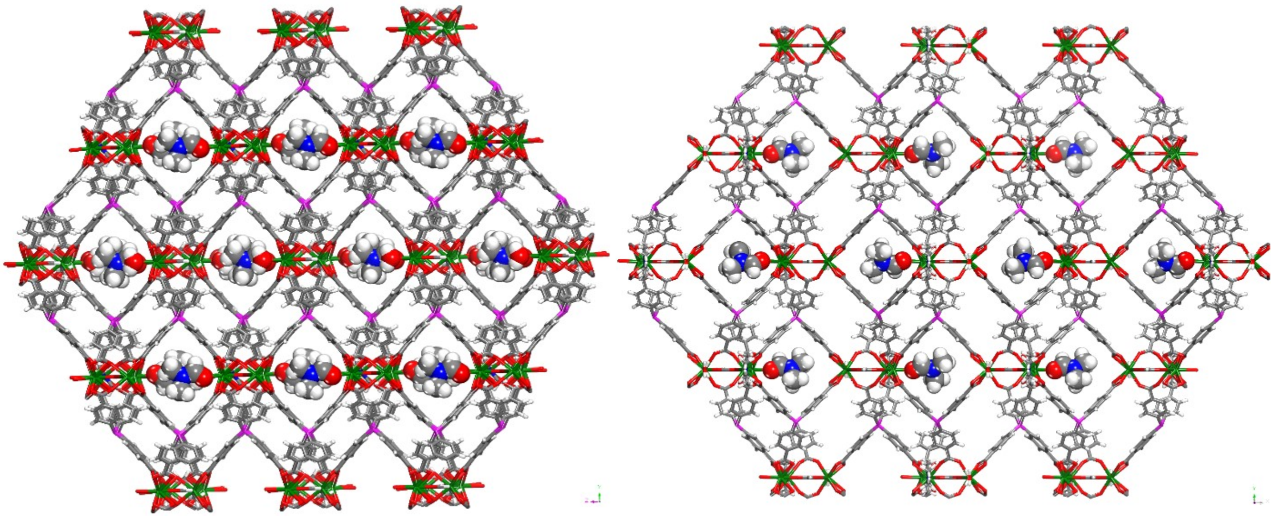

5.8 Figure S8. The possible ion transport channels in the pristine TB-MOF

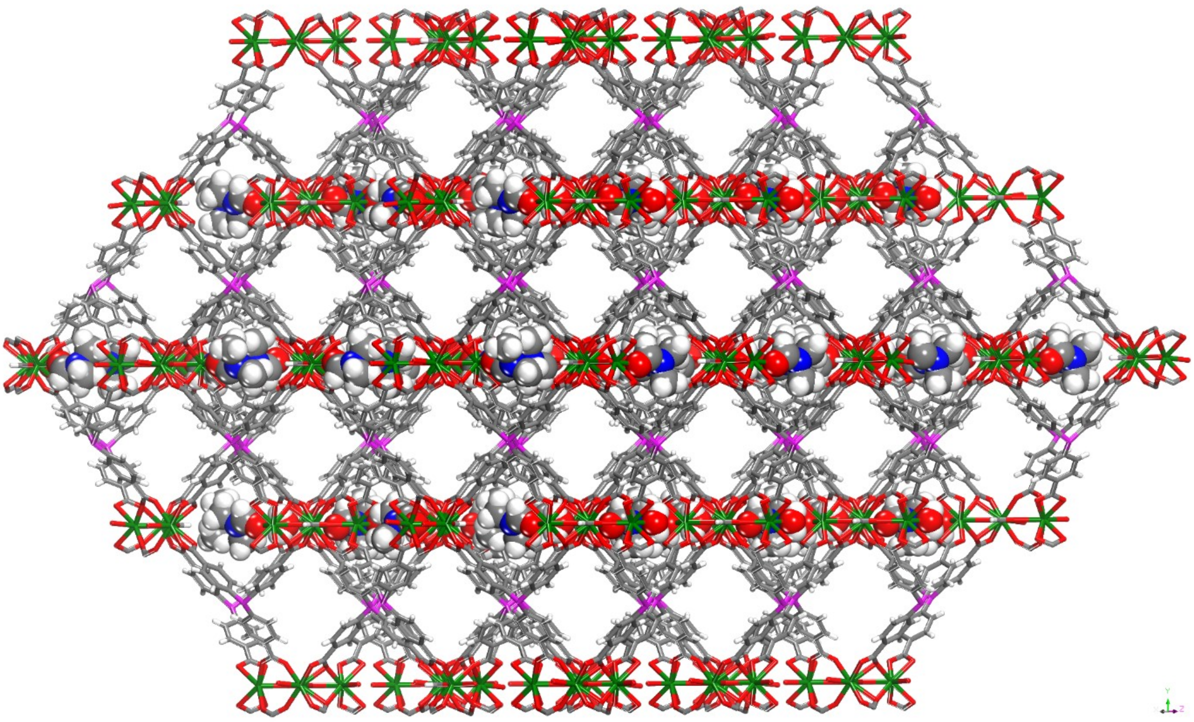

6. Figure S9. ^{7}Li MAS NMR spectra.

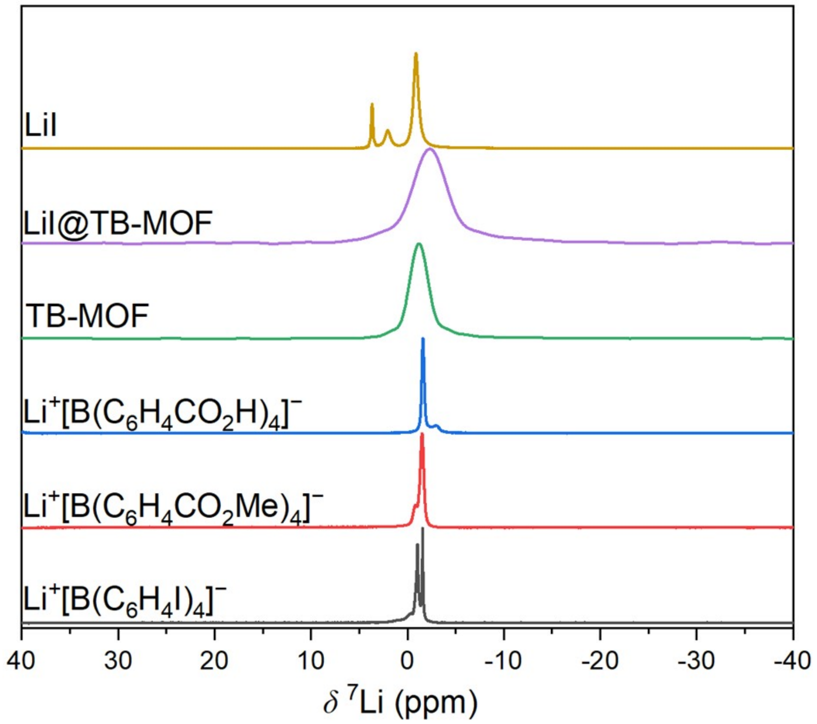

7. Figure S10. TGA of TB-MOF

(Including: Pristine, Activated, Immersing in PC)

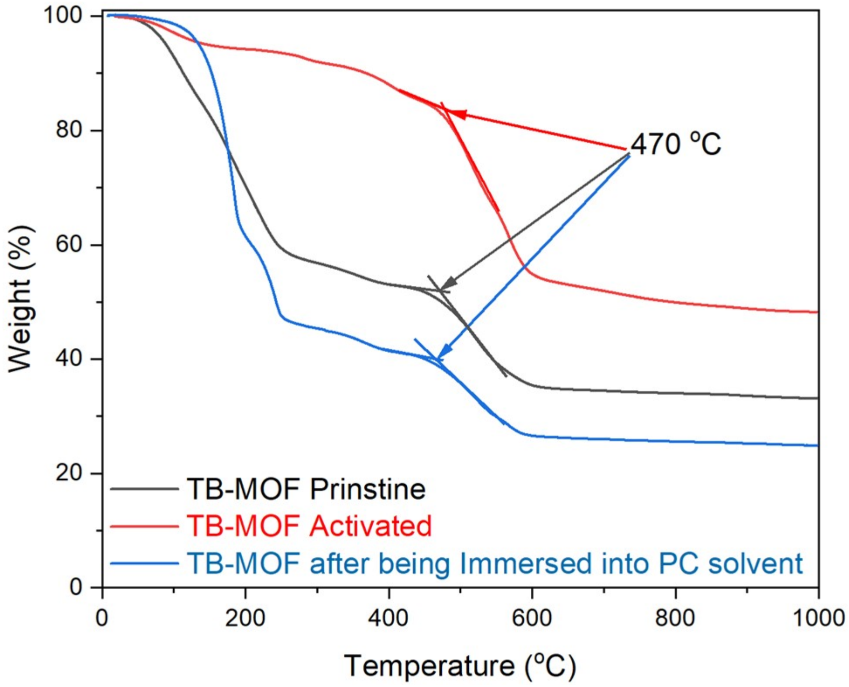

8. Figure S11-S13. ^{11}B MAS NMR, ^{11}B NMR, and ^{13}C NMR

8.1 Figure S11. ^{11}B MAS NMR of TB-MOF

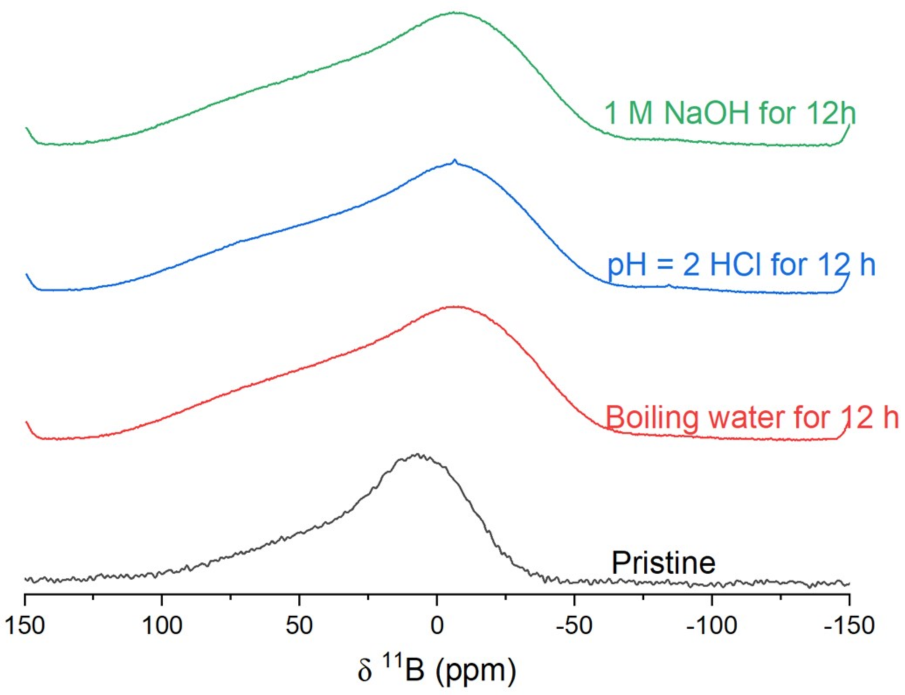

8.2. Figure S12. ^{11}B NMR of $Li^+[B(C_6H_4I)_4]^-$ and $Li^+[B(C_6H_4COOH)_4]^-$after treating in 1M HCl and 1M NaOH solution

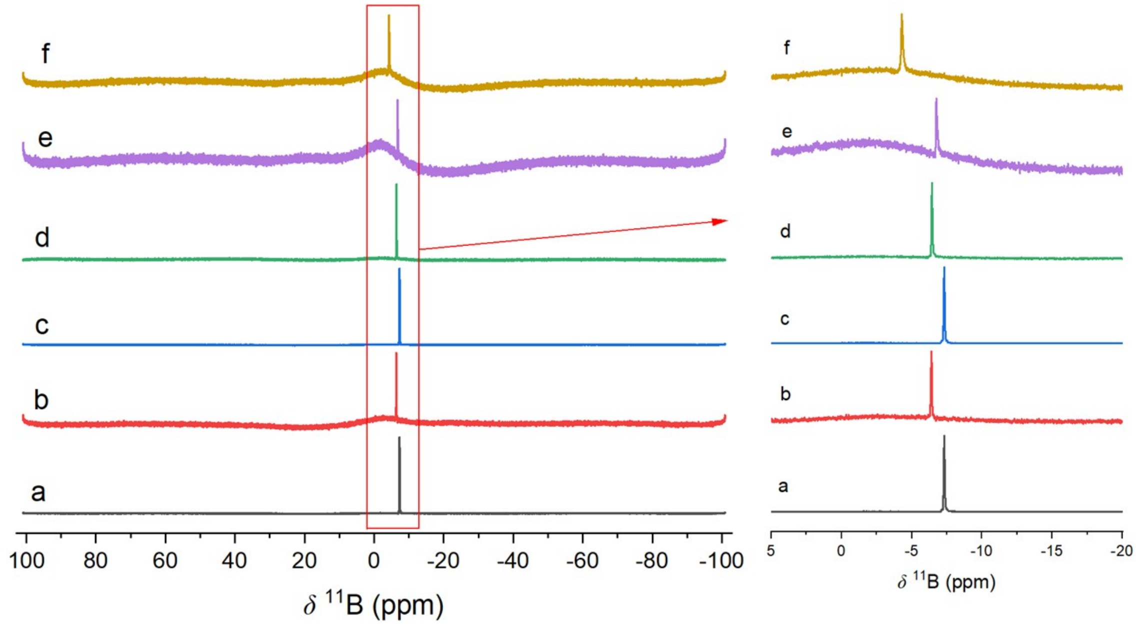

a, c) ^{11}B NMR of the ligand $Li^+[B(C_6H_4COOH)_4]^-$ after immersing in 1M HCl and 1M NaOH at 100 ^oC for more than 24h, respectively.

b, d) ^{11}B NMR of the compound $Li^+[B(C_6H_4I)_4]^-$ after immersing in 1M HCl and 1M NaOH at 100 ^oC for more than 24h, respectively.

e) ^{11}B NMR of TB-MOF after being decomposed by 3 M NaOH.

f) ^{11}B NMR of the reaction mixture after solvothermal reaction.

(The reaction mixture was the mixture of DMF/MeOH (10mL) that dissolved $ErCl_3·6H_2O$ (0.2 mmol), lithium tetrakis(4-carboxyphenyl)borate (H_4**L**) (0.25 mmol) and HCOOH (3 mL) then heated at 80 °C for 48 h. The HCOOH that used more than 3 mL was to restrain the formation of TB-MOF.)

8.3. Figure S13. ^{13}C NMR of $Li^+[B(C_6H_4COOH)_4]^-$ and TB-MOF after treating by 3M NaOH

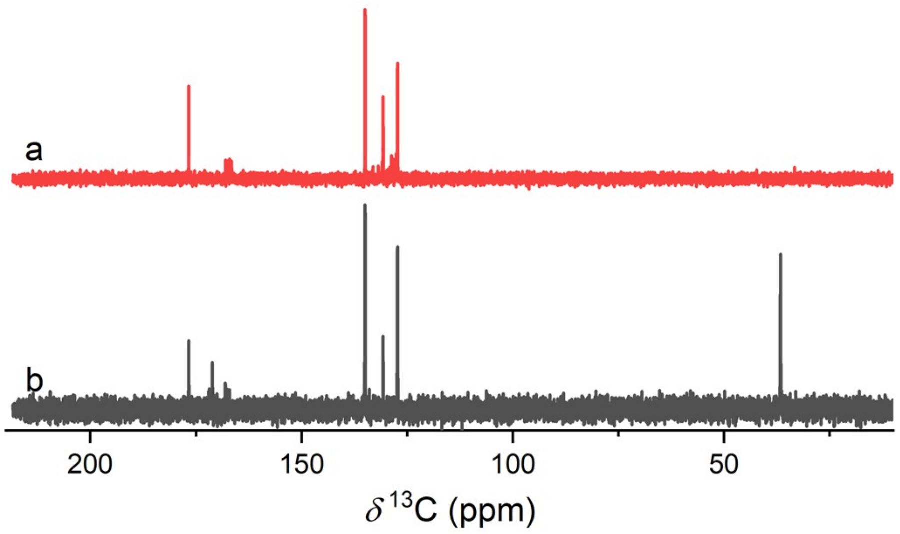

a) ^{13}C NMR of the ligand $Li^+[B(C_6H_4COOH)_4]^-$ after being dissolved into 3 M NaOH solution.
b) ^{13}C NMR of the TB-MOF after being decomposed by 3 M NaOH.

9. Figure S14.PXRD patterns of TC-MOF

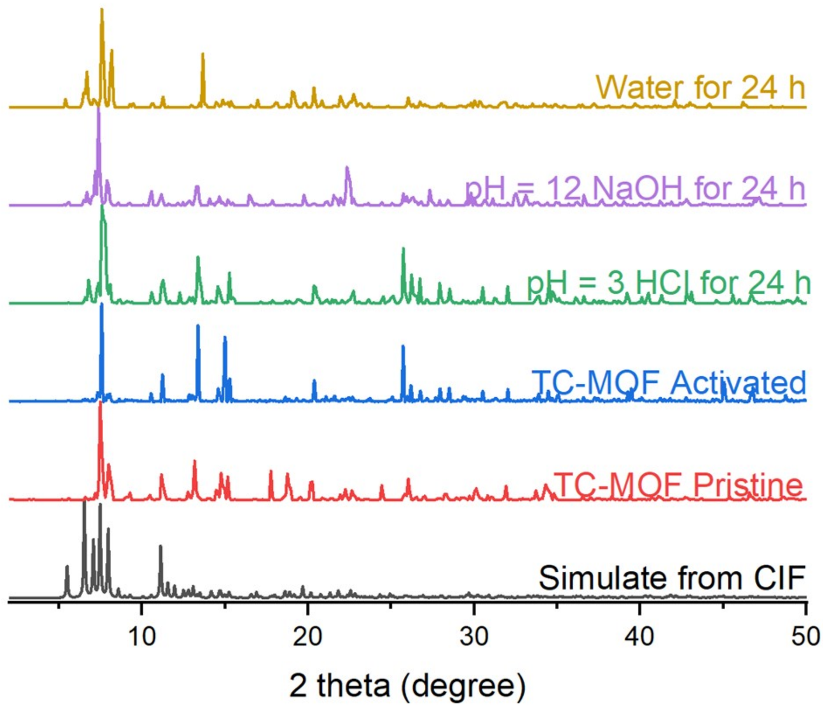

10. Figure S15. BET surface area of TC-MOF

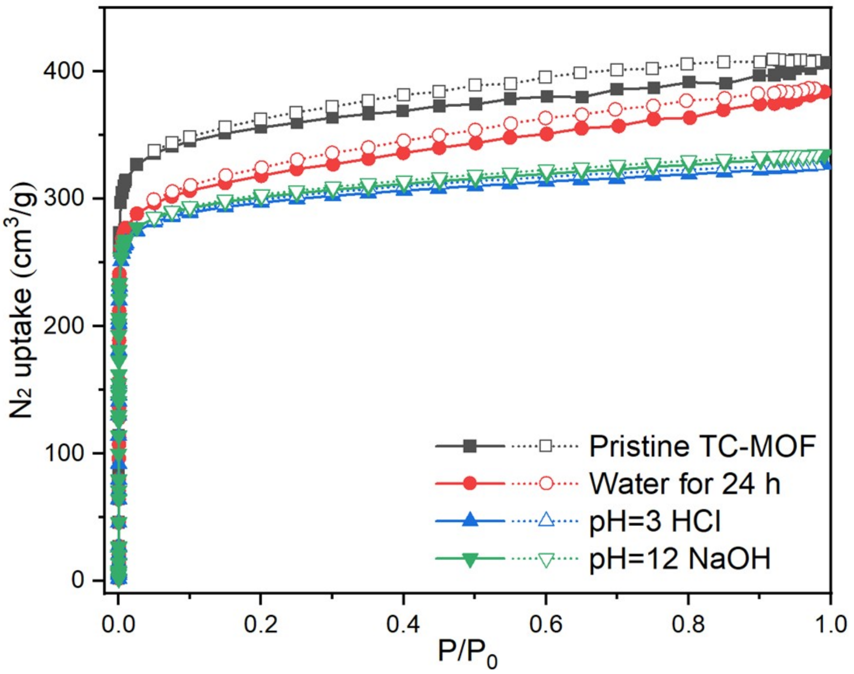

11. Figure S16-S21. Electronic and ionic conductivities

11.1 Figure S16. The electronic conductivity of TB-MOF

The electronic conductivity of TB-MOF determined by direct current (DC) polarization measurements

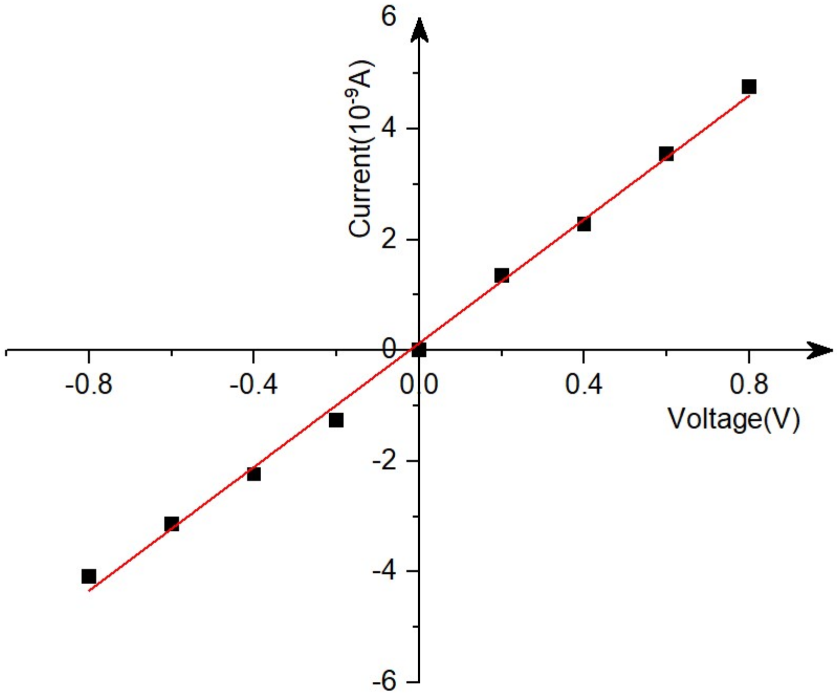

11.2 Figure S17. Ionic conductivity of TC-MOF

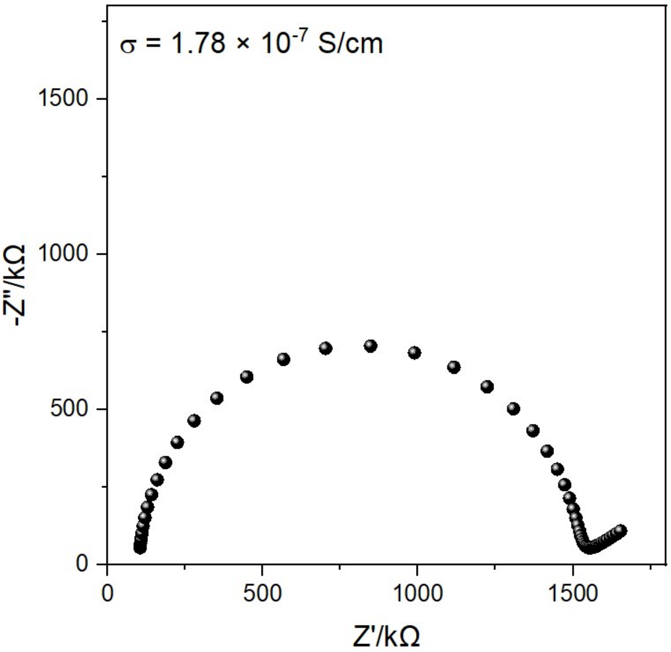

After TC-MOF provided by solvothermal reactions, they were then washed with MeOH, dried under vacuum at room temperature, immersed in propylene carbonate (PC) solvent for 24 h. After that, the white powder TC-MOF was collected by filtration or centrifugation, simply washed with THF, and then dried under vacuum to afford the free-flowing dry powder. In an Ar glovebox, 50 mg TC-MOF was pressed into a pellet under at 5 Mpa force, ranged from 1.0 to 1.4 mm and assembled into an airtight Swagelok-type cell between stainless steel blocking electrodes for the ionic conductivity (SS|TC-MOF|SS).

11.3 Figure S18. Ionic conductivity of Li[B(C_6H_4COOMe)$_4$]

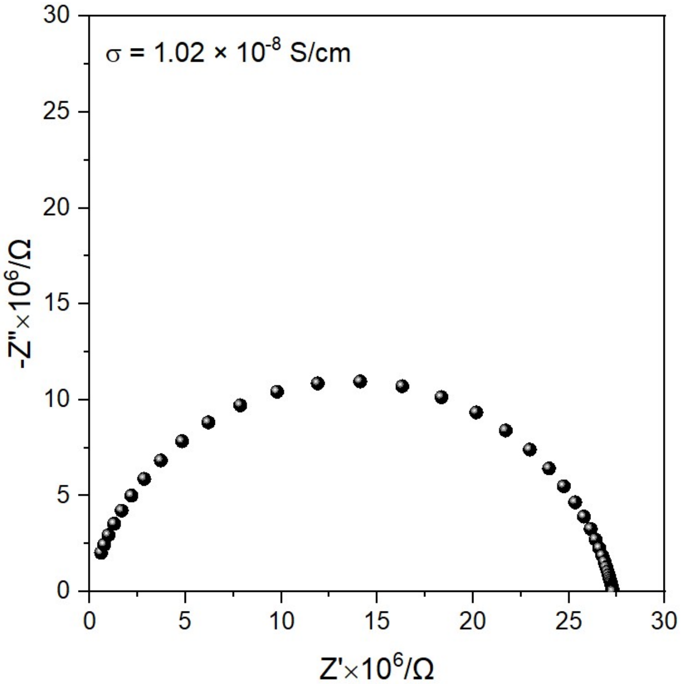

Typically, two drop of propylene carbonate (PC) solvent was added into 50 mg **Li[B(C_6H_4COOMe)$_4$]**, then the resulting powder was pressed into a pellet in the Ar glovebox, ranged

from 1.0 to 1.4 mm and assembled into an airtight Swagelok-type cell between stainless steel blocking electrodes for the ionic conductivity (SS|**Li[B(C_6H_4COOMe)$_4$]**|SS).

11.4 Figure S19. Ionic conductivity of the activated and pristine TB-MOF

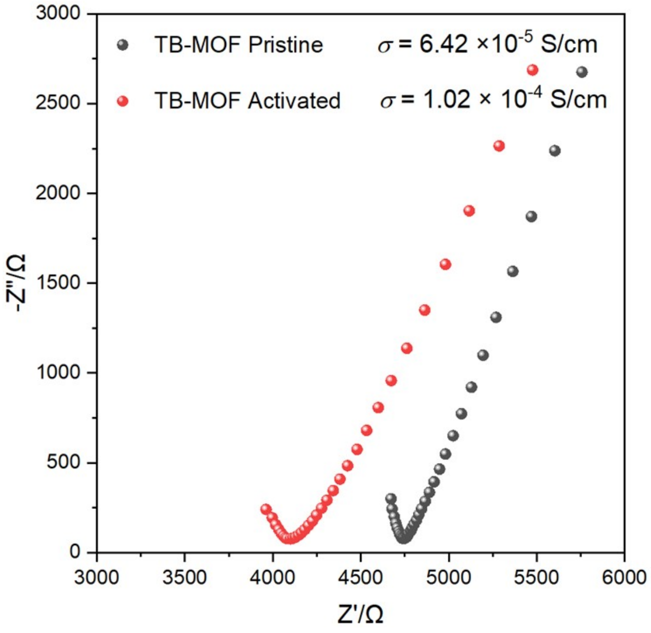

11.5 Figure S20. Ionic conductivity of TB-MOF after immersing in the PC solution of $LiBF_4$

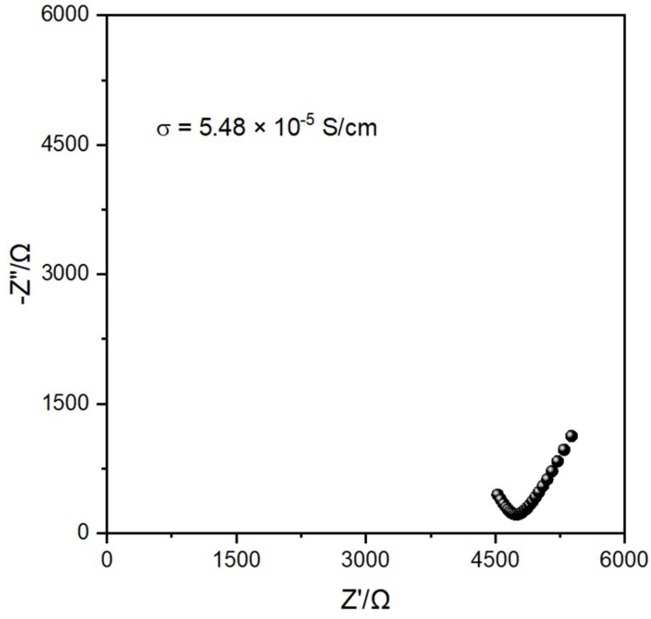

11.6 Figure S21. Ionic conductivity of TB-MOF by mixing with 5 wt% LiTFSI

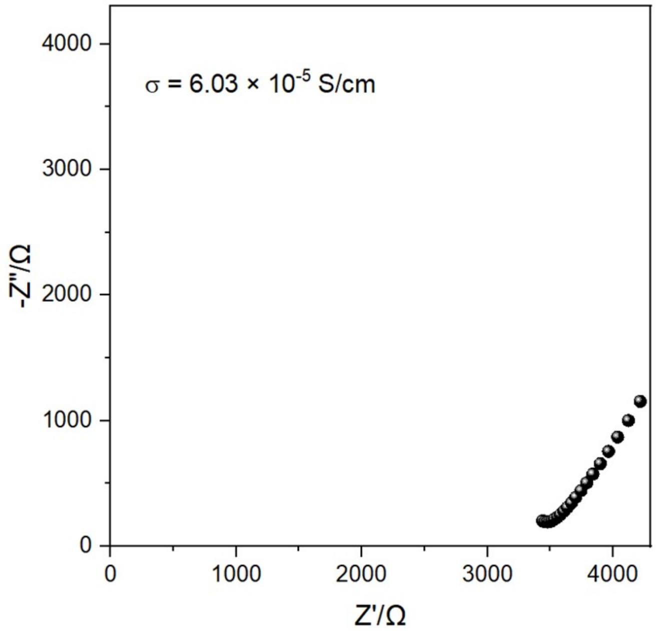

12. XPS and XAFS spectroscopy

12.1 Figure S22. XPS spectra of TB-MOF and LiI@TB-MOF

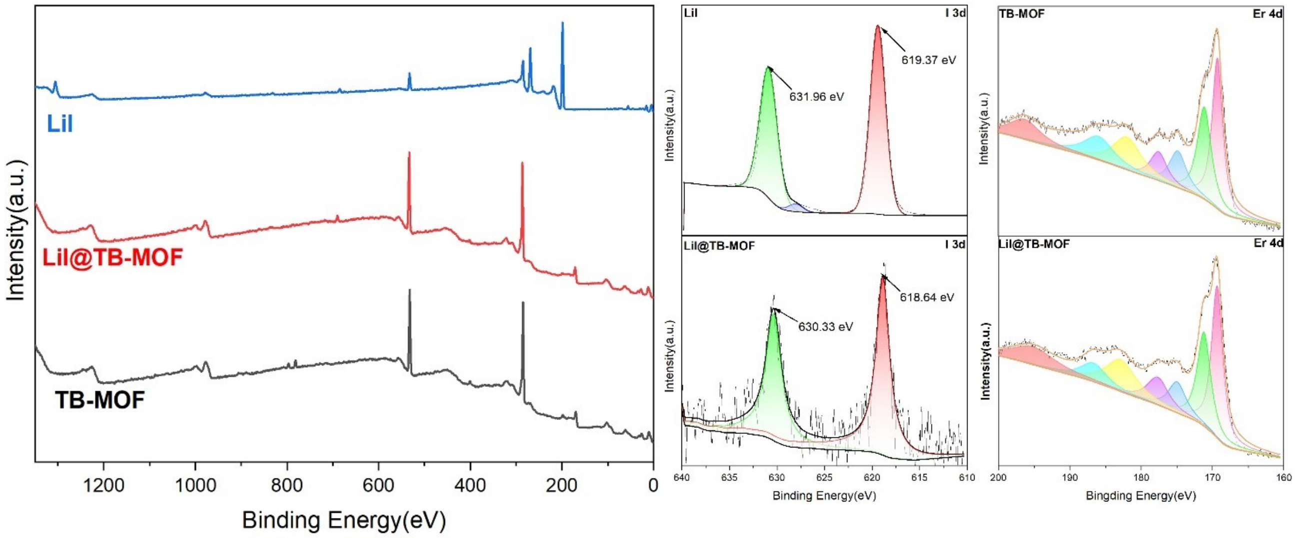

12.2 Figure S23. X-ray absorption fine structure (XAFS) spectroscopy of TB-MOF and LiI@TB-MOF

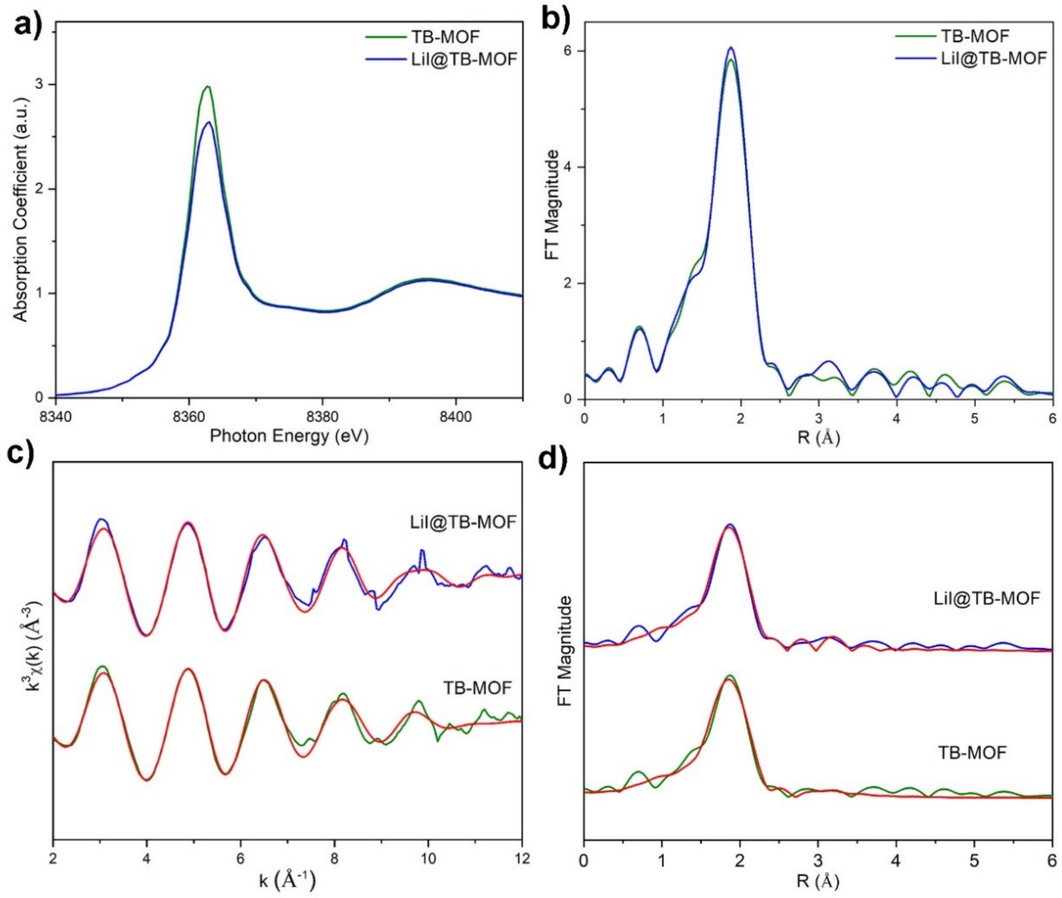

a) The Er L_3-edge XANES spectra of TB-MOF and LiI@TB-MOF. b) The Er L_3-edge EXAFS data of TB-MOF and LiI@TB-MOF. c) k^3-weighted Er L_3-edge experimental $\chi(k)$ data (blue and green) and fit (red) in k-space. b) The corresponding Fourier transform for TB-MOF and LiI@TB-MOF.

12.3 Table S2. Structure parameters of TB-MOF and LiI@TB-MOF derived from the Er L_3-edge EXAFS fitting results.

Sample	Paths	N	R(Å)	σ^2($\times10^{-3}$ Å^2)	ΔE_0(eV)
TB-MOF	Er-O	3.5 ± 0.5	2.26 ± 0.02	5.2 ± 1.6	3.4
	Er-O	3.5 ± 0.5	2.41 ± 0.02	5.0 ± 1.9	9.1
LiI@TB-MOF	Er-O	3.5 ± 0.5	2.27 ± 0.02	4.4 ± 1.5	4.2
	Er-O	3.5 ± 0.5	2.42 ± 0.02	4.7 ± 2.0	9.2
	Er-I	0.8 ± 0.3	3.25 ± 0.02	10.0 ± 2.8	-15.0

The X-ray absorption spectroscopy (XAS) data at the Er L_3-edge were recorded on beamline 14W1 at the Shanghai Synchrotron Radiation Facility (SSRF). The electron beam energy was 3.5 GeV with a stored current of approximately 200 mA in top-up operation. A fixed-exit double crystal Si <111> monochromator was used for the incident energy selection. XAS data of the samples were acquired in transmission mode by using two ionization chambers filled with nitrogen. Standard procedures were followed to analyze the XAS data using the software package Demeter (J. Synchrotron. Radiat. 2005, 12, 537-541). The backscattering amplitude and phase shift were calculated with the program FEFF9 (Phys. Chem. Chem. Phys. 2010, 12 (21), 5503-5513). The XAS data were calibrated, averaged, pre-edge background subtracted, and post-edge normalized using the Athena program. Theoretical fittings

for the EXAFS data were executed using the Artemis program. The Fourier transformation of the k^3-weighted EXAFS oscillations from k-space to r-space was performed over a range of 2.5−12.0 Å^{-1}. A window of 1.0−3.8 Å in r-space was applied for the fitting of EXAFS data. The value of amplitude reduction factor S_0^2 was fixed at 0.98 during the fitting for the samples. Other structural parameters, such as coordination numbers (N), bond distance (R), Debye-Waller factor (σ^2), and inner potential shift (ΔE_0), were obtained from the fitting.

X-ray absorption near edge structure (XANES) data shows LiI@TB-MOF sample has the same absorption edge position as TB-MOF (Fig. a), revealing the same oxidation state of Er. The white lines at approximately 8363 eV in the XANES spectra are ascribed to the electron transition from Er $2p_{3/2}$ to unoccupied 5d states. The white line intensity of LiI@TB-MOF is higher than that of TB-MOF, implying the increase of the unoccupied Er 5d states caused by the Er-I interaction. The extended X-ray absorption fine structure (EXAFS) data shows that LiI@TB-MOF displays similar spectral profile to TB-MOF (Fig. b). A close observation of the EXAFS spectra reveals that the LiI@TB-MOF has a stronger peak near 3 Å than the TB-MOF, which can be attributed to the Er-I scattering contribution. The EXAFS fitting result shows that the TB-MOF can be well fitted by using two Er-O shells with average bond lengths of ~ 2.26 and ~ 2.41 Å (Fig. c), respectively. Besides two similar Er-O shells, the LiI@TB-MOF has one Er-I shell with average coordination number of 0.8 ± 0.3 and average bond length of ~ 3.25 Å.

13. Figure S24. SEM and SEM-EDS mapping of LiI@TB-MOF

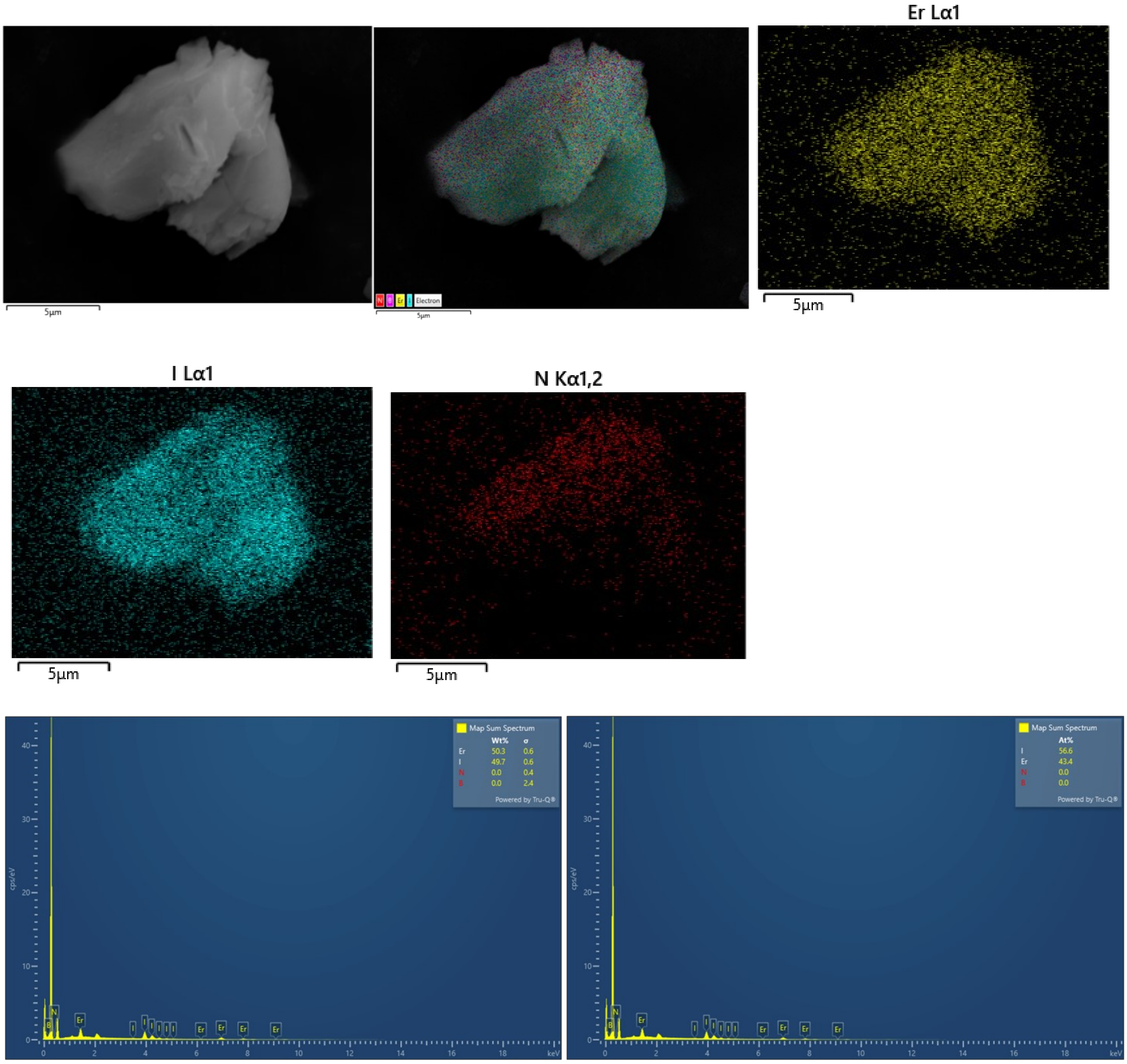

14. Figure S25. IR spectrum of TB-MOF

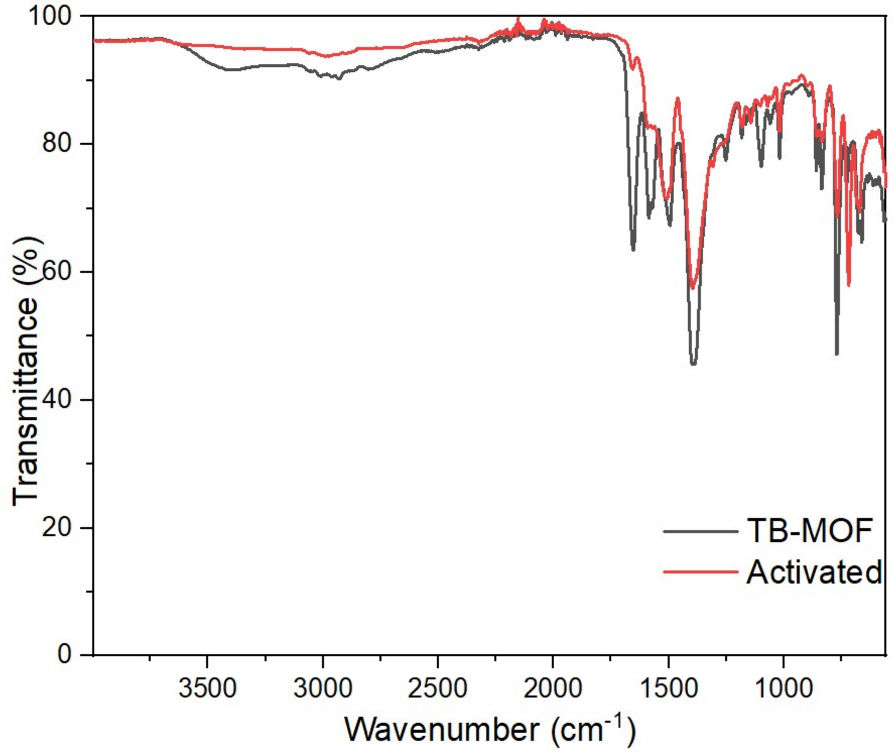

15 Figure S26. TGA cure of LiX@TB-MOF and M@TB-MOF
(After immersing in PC)

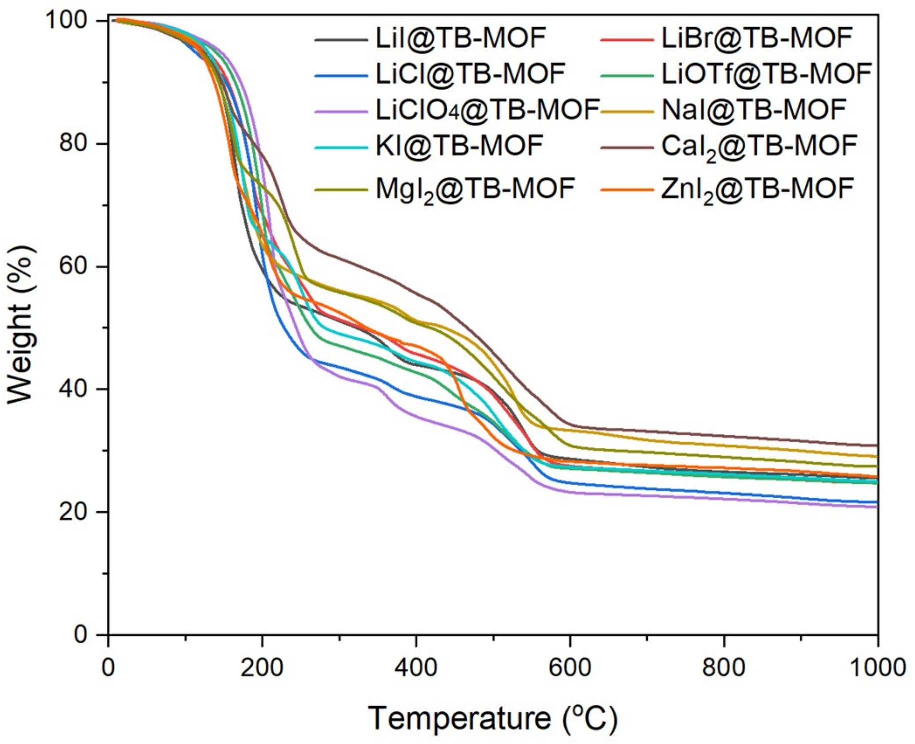

16. Figure S27~S31 and Table S3. Ionic conductivity and Li^+ transference number of LiX@TB-MOF

16.1 Figure S27. Ionic conductivity and Li^+ transference number of LiI@TB-MOF

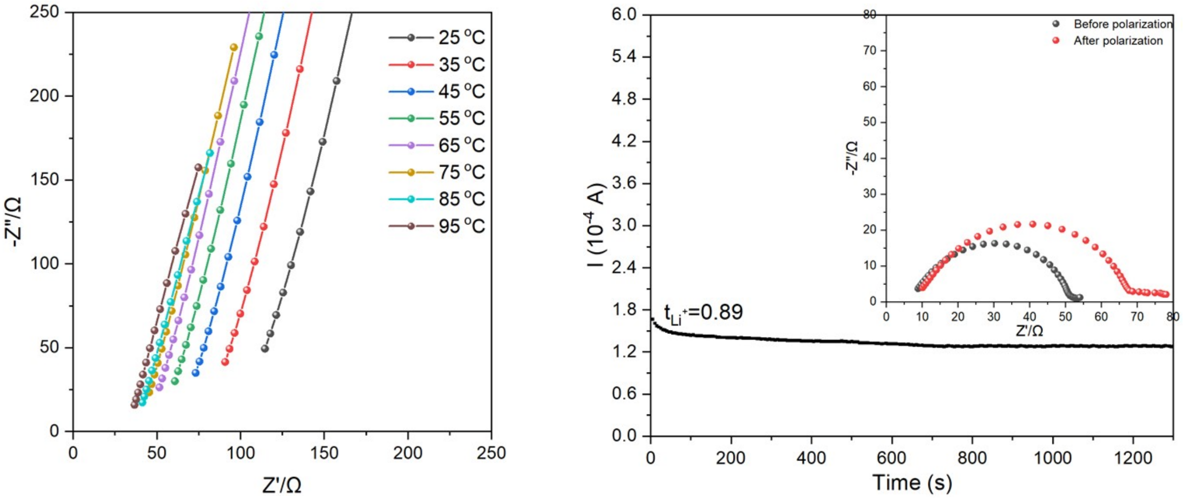

16.2 Figure S28. Ionic conductivity and Li^+ transference number of LiBr@TB-MOF

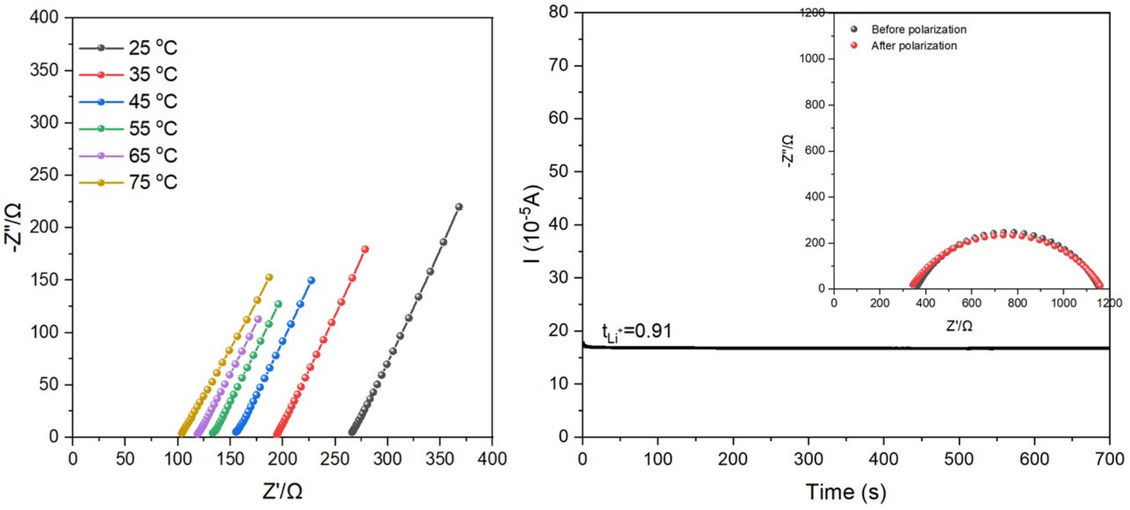

16.3 Figure S29. Ionic conductivity and Li^+ transference number of LiCl@TB-MOF

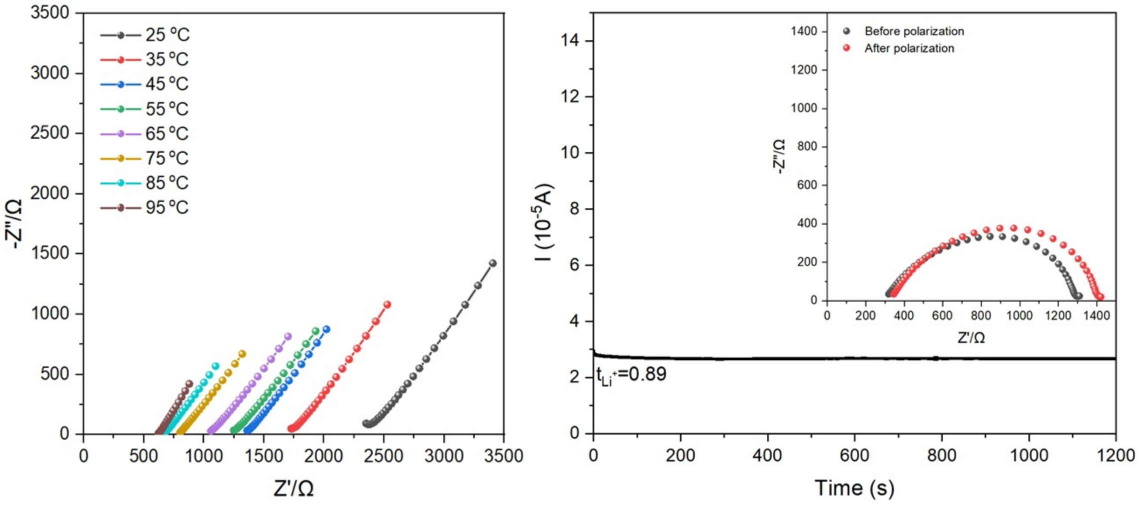

16.4 Figure S30. Ionic conductivity and Li^+ transference number of $LiClO_4$@TB-MOF

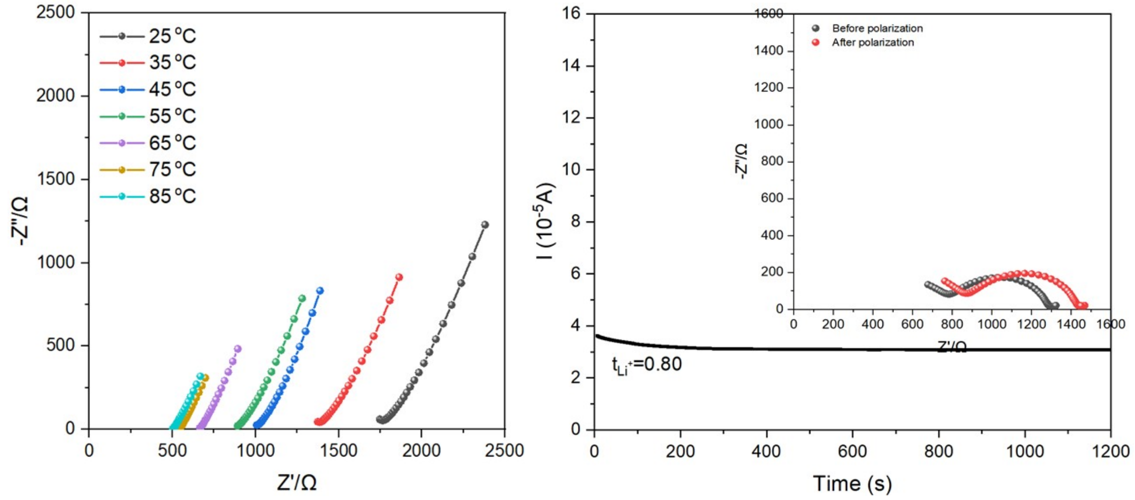

16.5 Figure S31. Ionic conductivity and Li^+ transference number of LiOTf@TB-MOF

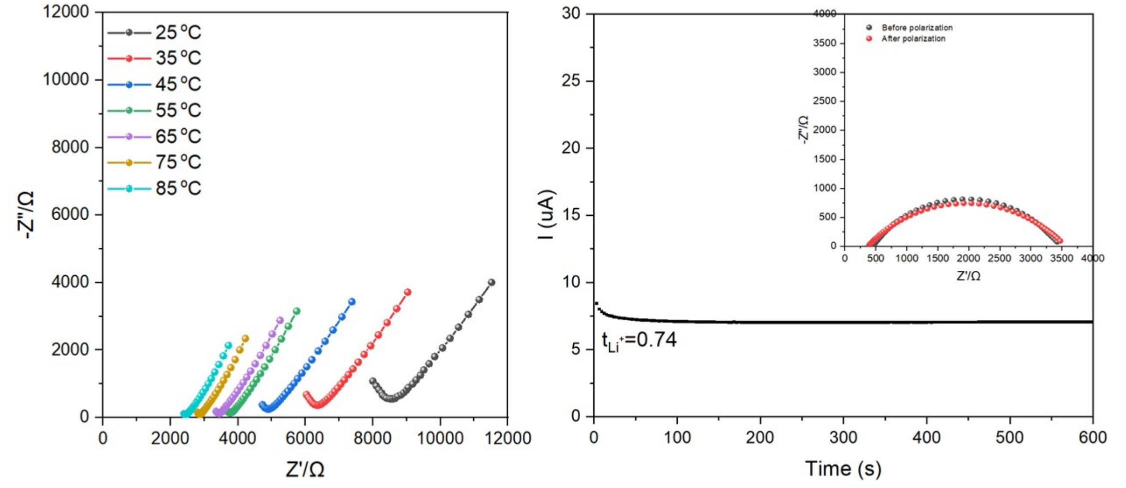

16.6 Table S3. Formulas of LiX@TB-MOF, Li^+ Content, Ionic Conductivity, Activation Energy, and Li^+ Transference Number Values

Electrolyte	Formula	Li^+ content (wt %)	σ (S/cm)	E_a (eV)	t_{Li+}
TB-MOF	$Li_2\cdot[Er_3(\mathbf{L})_2(HCOO)(DMF)_2(H_2O)]\cdot 19PC$	0.82	6.42×10^{-5}	0.23	0.70
LiI@TB-MOF	$Li_5\cdot[Er_3(\mathbf{L})_2(HCOO)I_3]\cdot 16PC$	1.80	2.75×10^{-3}	0.15	0.89
LiBr@TB-MOF	$Li_5\cdot[Er_3(\mathbf{L})_2(HCOO)Br_3]\cdot 15PC$	1.94	1.33×10^{-3}	0.19	0.91
LiCl@TB-MOF	$Li_5\cdot[Er_3(\mathbf{L})_2(HCOO)Cl_3]\cdot 16PC$	2.09	1.34×10^{-4}	0.21	0.89
$LiClO_4$@TB-MOF	$Li_5\cdot[Er_3(\mathbf{L})_2(HCOO)(ClO_4)_3]\cdot 14PC$	1.88	1.51×10^{-4}	0.22	0.80
LiOTf@TB-MOF	$Li_5\cdot[Er_3(\mathbf{L})_2(HCOO)(OTf)_3]\cdot 14PC$	1.74	2.66×10^{-5}	0.22	0.74

17. Figure S32- S39. The interfacial compatibility and the SSB performance test

17.1 Figure S32. Voltage profiles of the galvanostatically cycled Li/LiI@TB-MOF/Li symmetrical cell

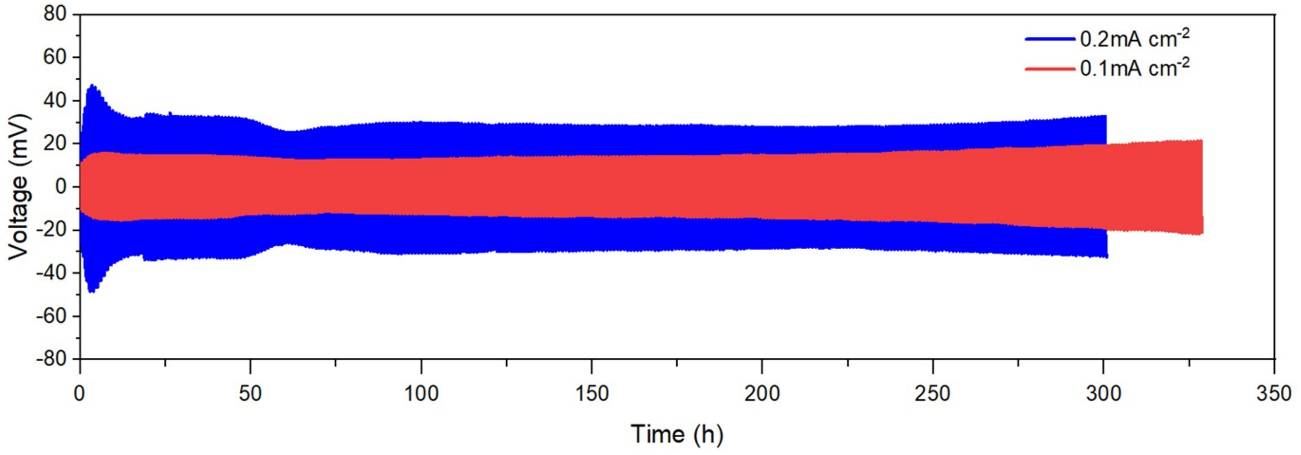

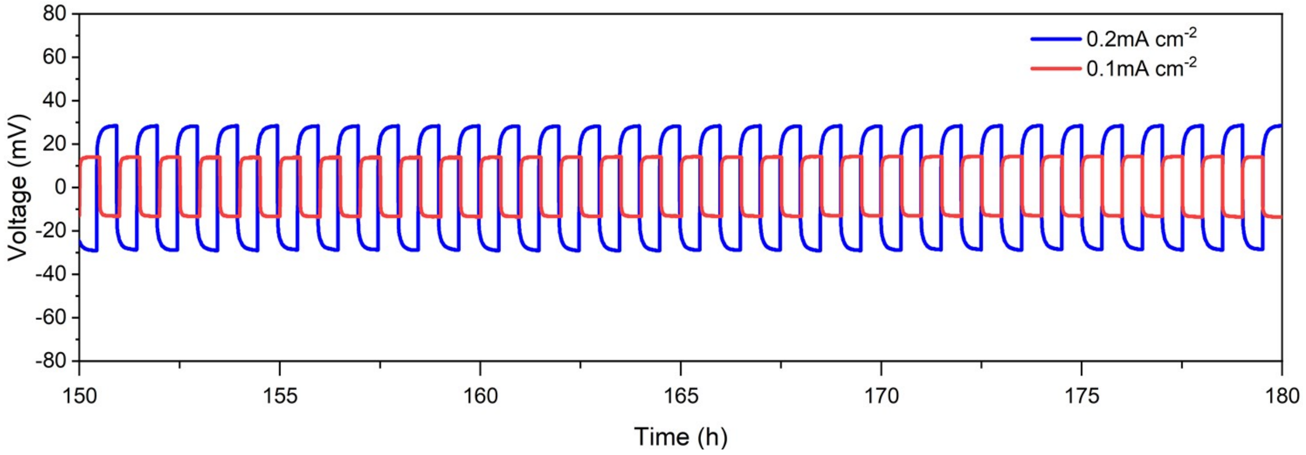

17.2 Figure S33. The interfacial compatibility between LiI@TB-MOF and Li metal anodes

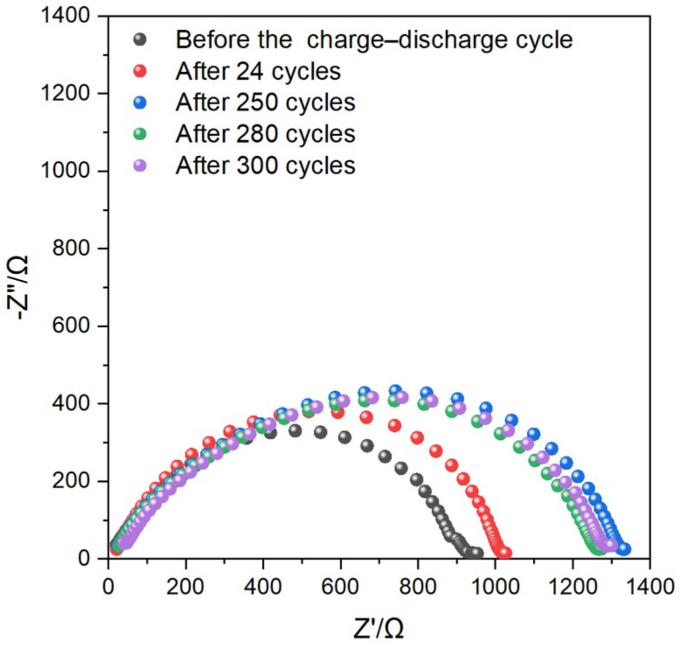

17.3 Figure S34. PXRD patterns after the AC impedance measurements

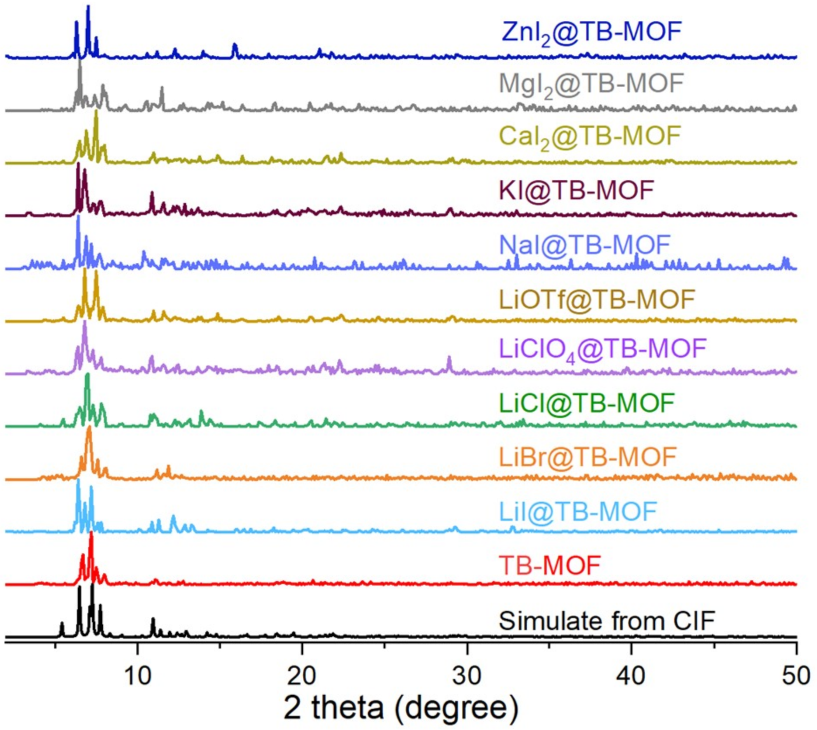

(Including: TB-MOF, M@TB-MOF (M=Li, Na, K, Mg, Ca, and Zn), LiI@TB-MOF after cycling 300 h, and Mg@TB-MOF after cycling 100 h)

17.4 Figure S35. BET surface area of LiI@TB-MOF before and after the galvanostatic cycling

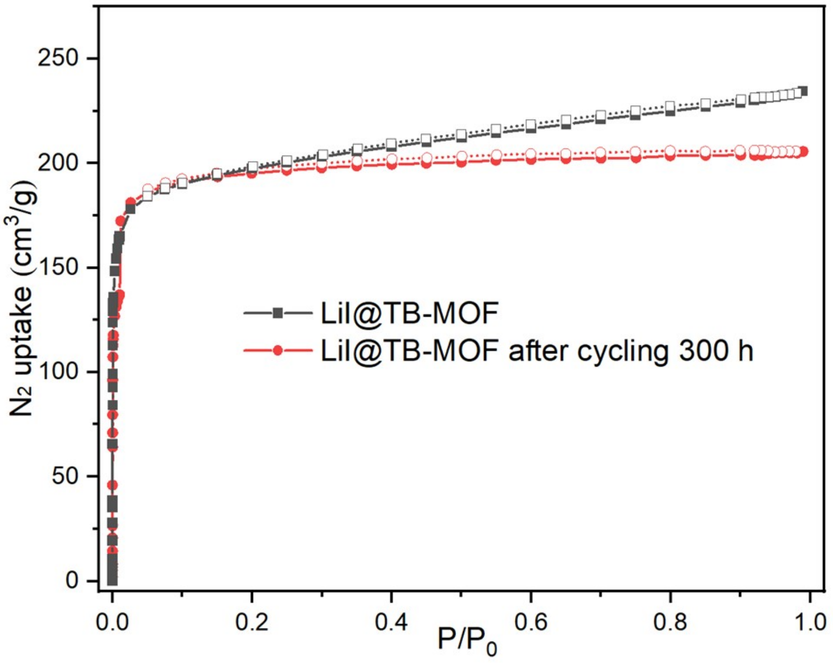

17.5 Figure S36. SEM morphology of the Li metal surface after Li plating/stripping cycles

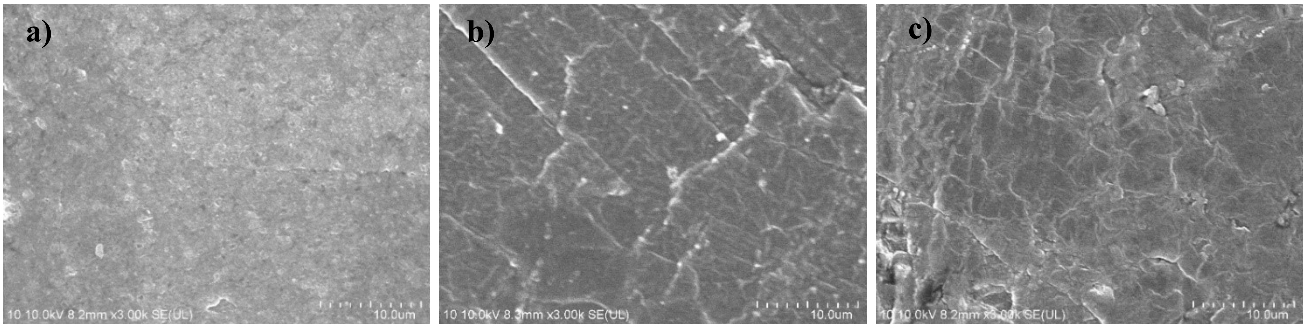

a) before cycling; b) after 300 cycles at 0.1mA/cm; c) after 300 cycles at 0.2 mA/cm^2.

17.6 Figure S37. Cycling performance and Coulombic efficiency of the Li/LiI@TB-MOF/LFP SSB with 0.5 C charge/discharge rate at room temperature.

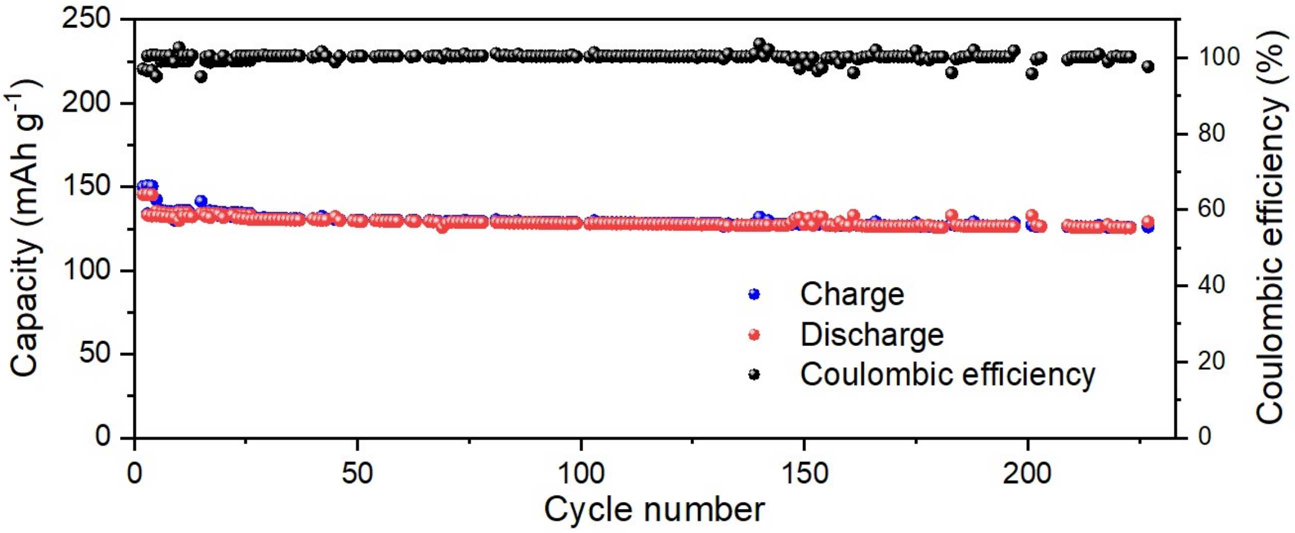

17.7 Figure S38. Charge-discharge voltage profiles of the Li/LiI@TB-MOF/LFP SSB at 0.5 C at room temperature.

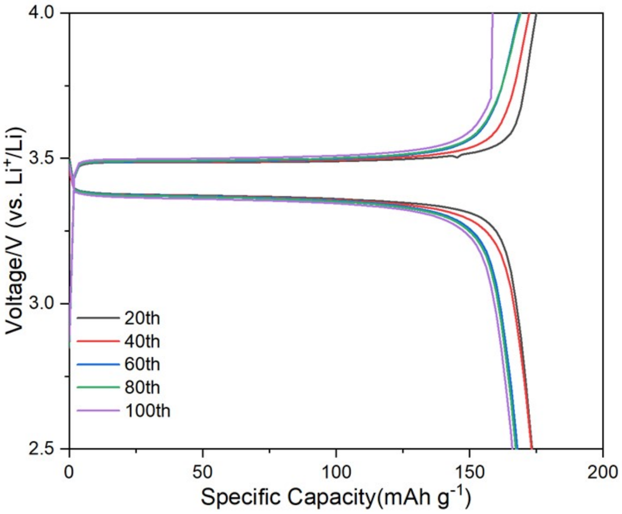

17.8 Figure S39. Specific capacity of Li/LiI@TB-MOF/LFP SSB at different C-rates

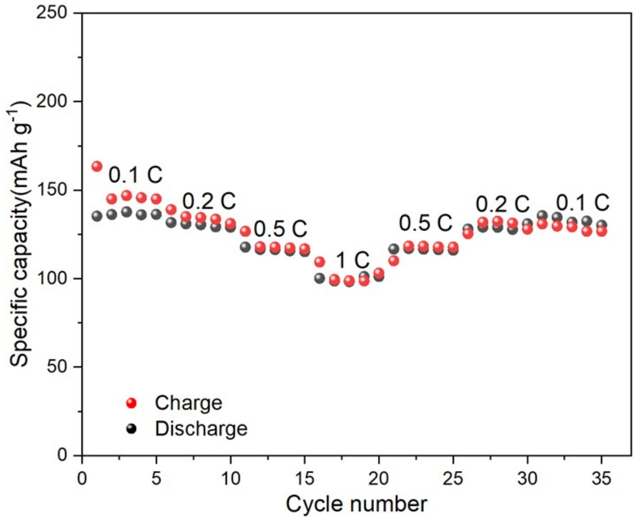

18. Figure S40. ^{7}Li NMR of lithium tetrakis(4-iodophenyl)borate

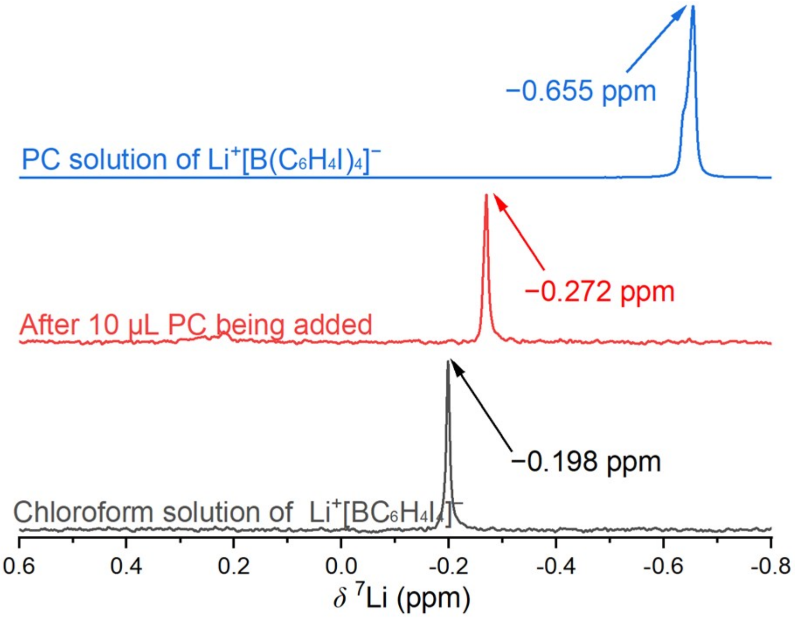

※ ^{7}Li NMR measurement of the intermediate $Li^{+}[B(C_6H_4I)_4]^{-}$ was performed to illustrate the solvation and interaction between Li^{+} and PC. Chloroform was selected as the solvent in order to avoid the influence of oxygen atom from the solvent. When the PC (10 μL) was added, the carbonyl oxygen atom of PC would coordinate to the Li^{+} ion with the lone-pair electron being filled into the 2s orbital, thereby increasing the extranuclear electron cloud of the Li atom and causing the ^{7}Li resonance shift from −0.198 ppm to −0.272 ppm. As more PC was added, especially when chloroform was completely replaced by PC, the coordination number of Li atoms reached its maximum, thereby leading to the highest electron cloud density outside and the highest shielding effect that caused a further shift to −0.655 ppm.

19. Figure S41. Size comparison between nanocage TB-MOF and PC molecule

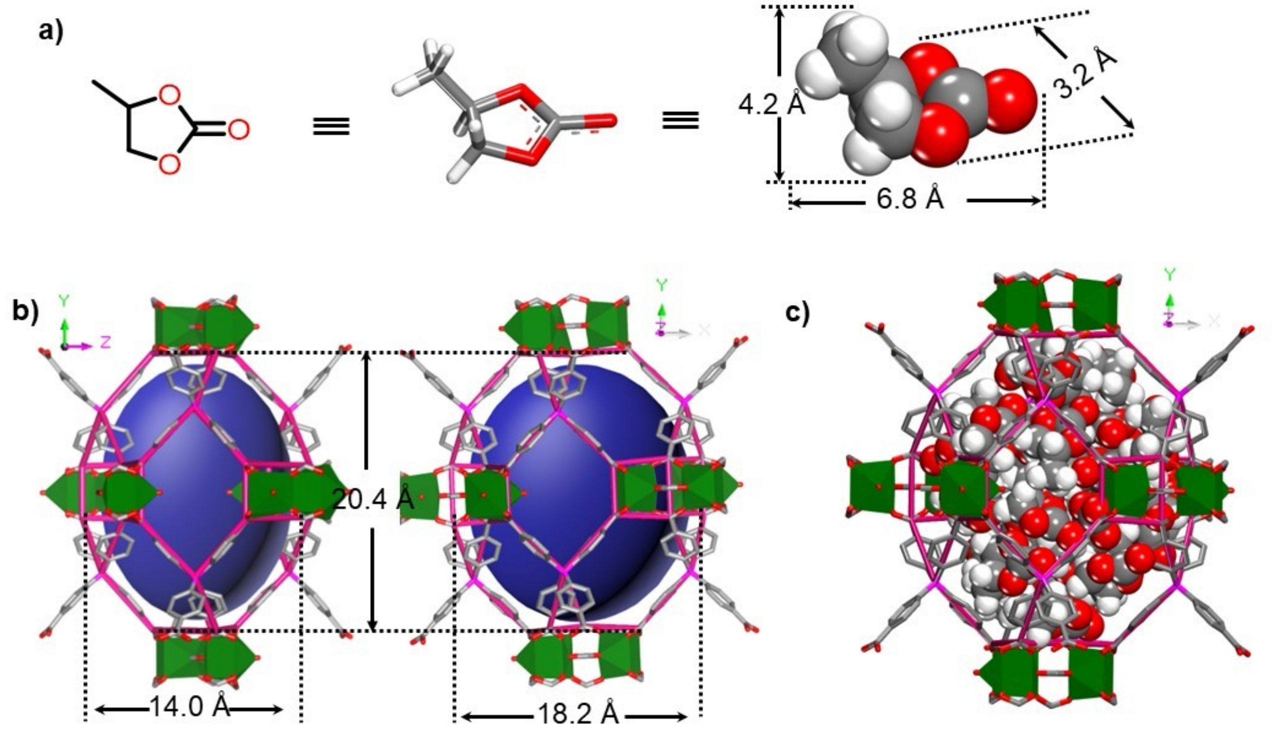

※ a) The size of the PC molecule. Simulated from Materials Studio (2019), the size is determined to be 4.2 × 6.8 × 3.2 $Å^3$, and the volume is then calculated to be ~91.4 $Å^3$. b) The cavity dimension of the distorted dodecahedral cage in TB-MOF is about 14.0 × 18.2 × 20.4 $Å^3$, and therefore, the volume of the distorted dodecahedral cage is approximately calculated to be ~2637.6 $Å^3$ (the volume of the ellipsoid), and each nanocage could hold about 29 PC molecules. c) Moreover, a distorted dodecahedral cage was taken out from the network, and it was employed to simulate the incorporation of PC solvent. Simulation from Materials Studio (2019) revealed that each nanocage could hold ~30 PC molecules.

20. Figure S41~S45. Ionic conductivity and transference number of MI*x*@TB-MOF

20.1 Figure S42. Ionic conductivity and transference number of NaI@TB-MOF

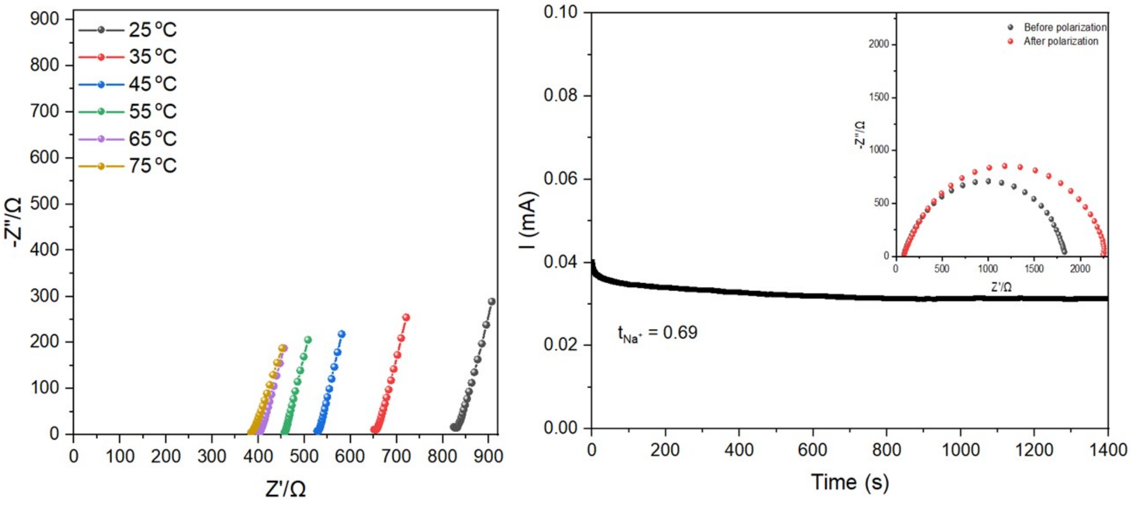

20.2 Figure S43. Ionic conductivity and transference number of KI@TB-MOF

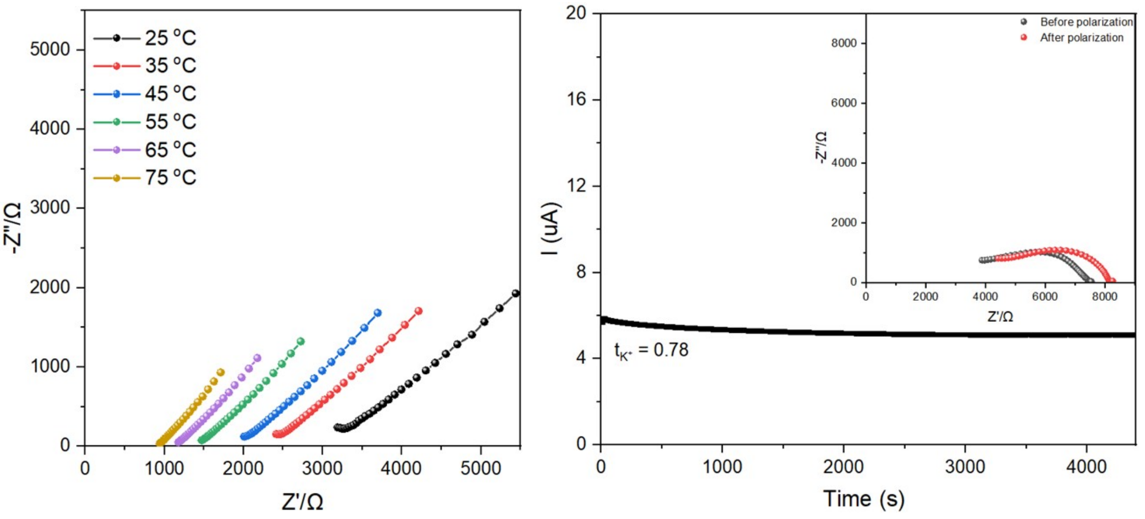

20.3 Figure S44. Ionic conductivity and transference number of MgI_2@TB-MOF

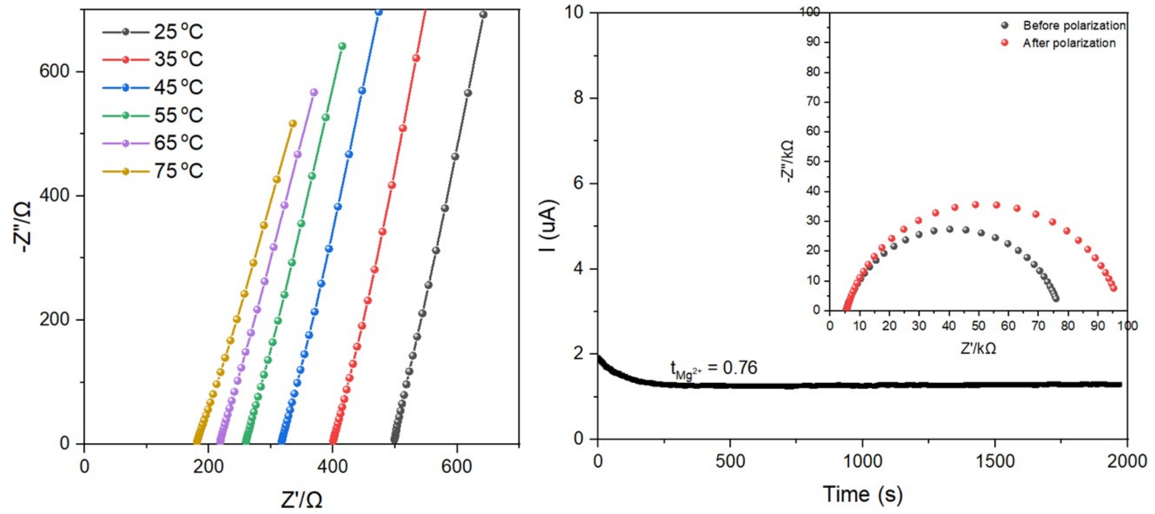

20.4 Figure S45. Ionic conductivity and transference number of CaI_2@TB-MOF

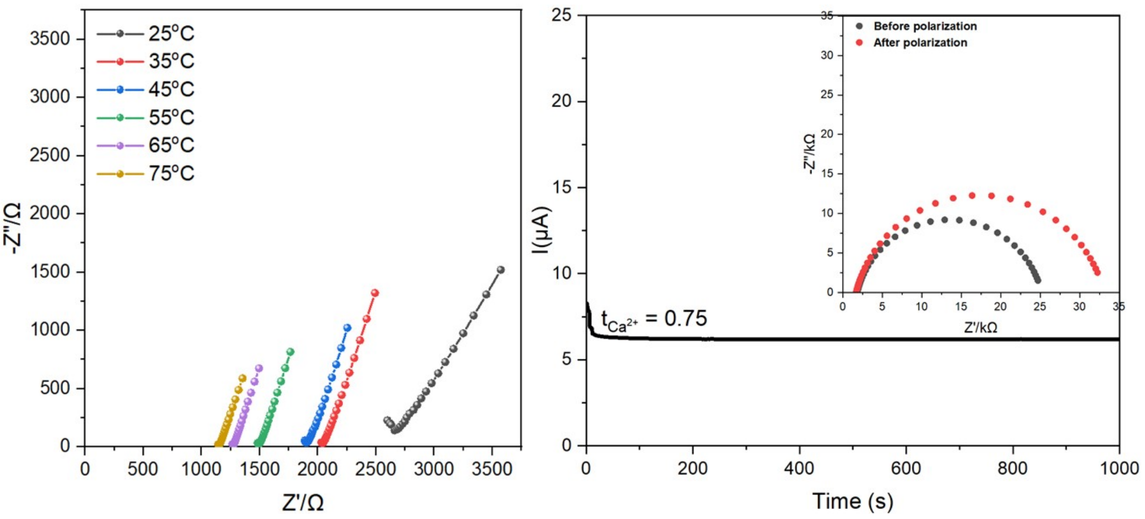

20.5 Figure S46. Ionic conductivity and transference number of ZnI_2@TB-MOF

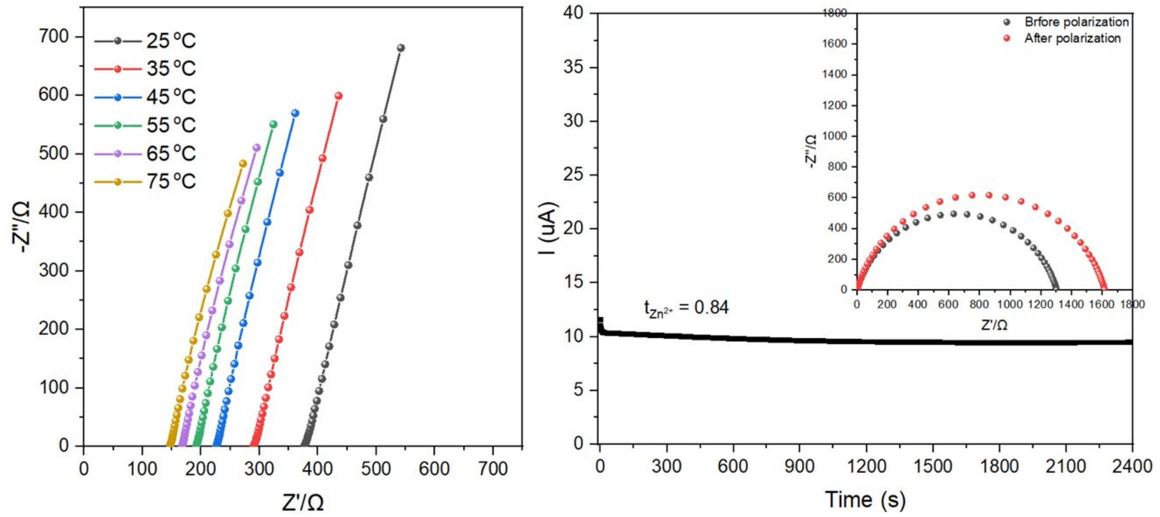

21. Figure S47. The galvanostatically cycle of MgI_2@TB-MOF

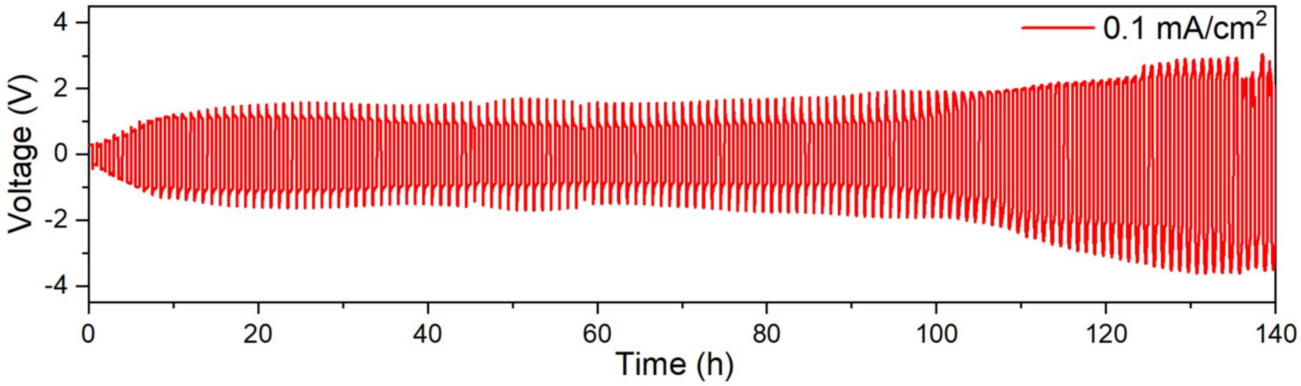

22. Figure S48. The micrographs of TB-MOF, and its scale-up synthesis.

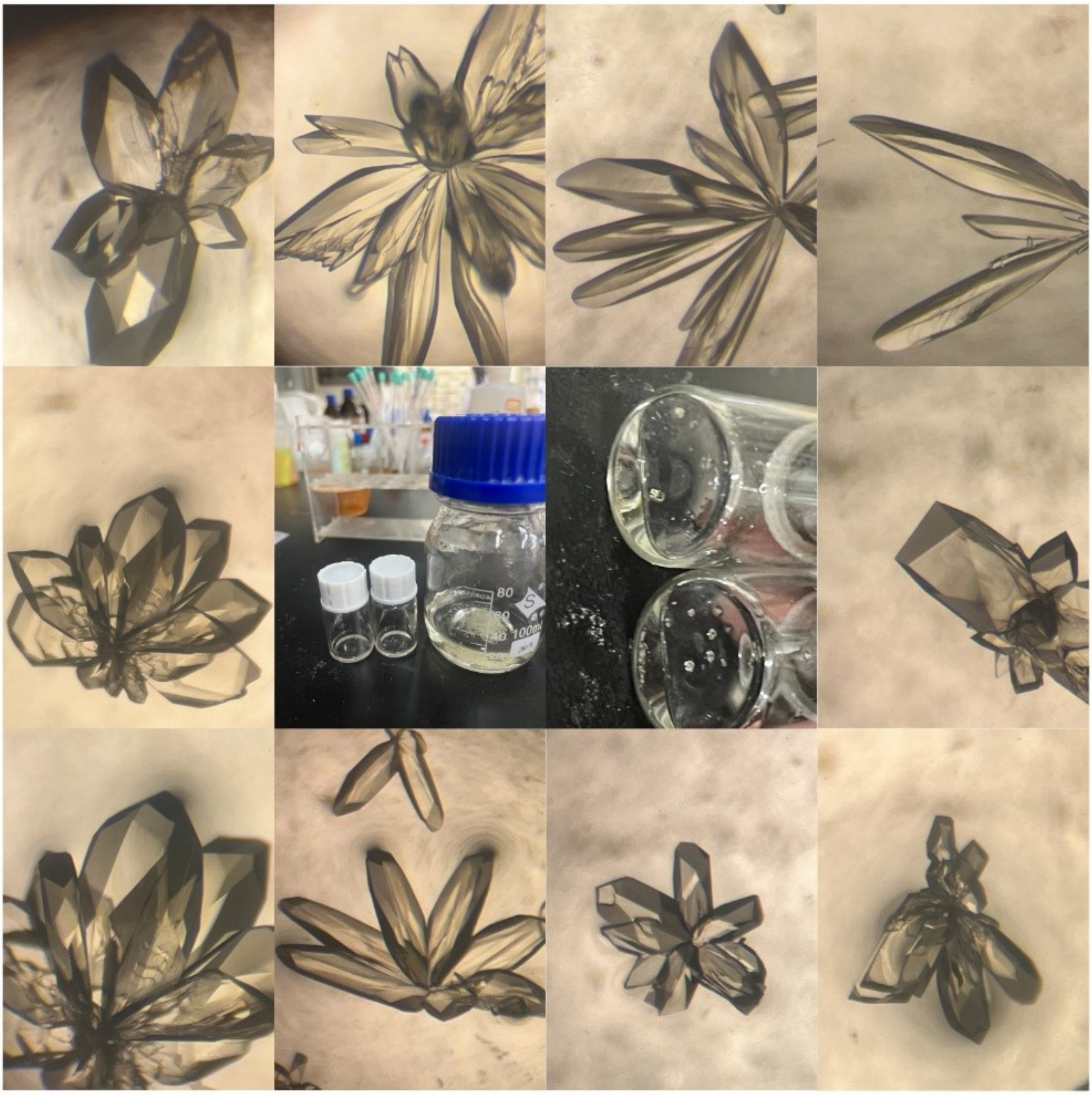

23. Figure S49-S57. Additional SEM and SEM-EDS mapping

23.1 Figure S49. SEM and SEM-EDS mapping of LiBr@TB-MOF

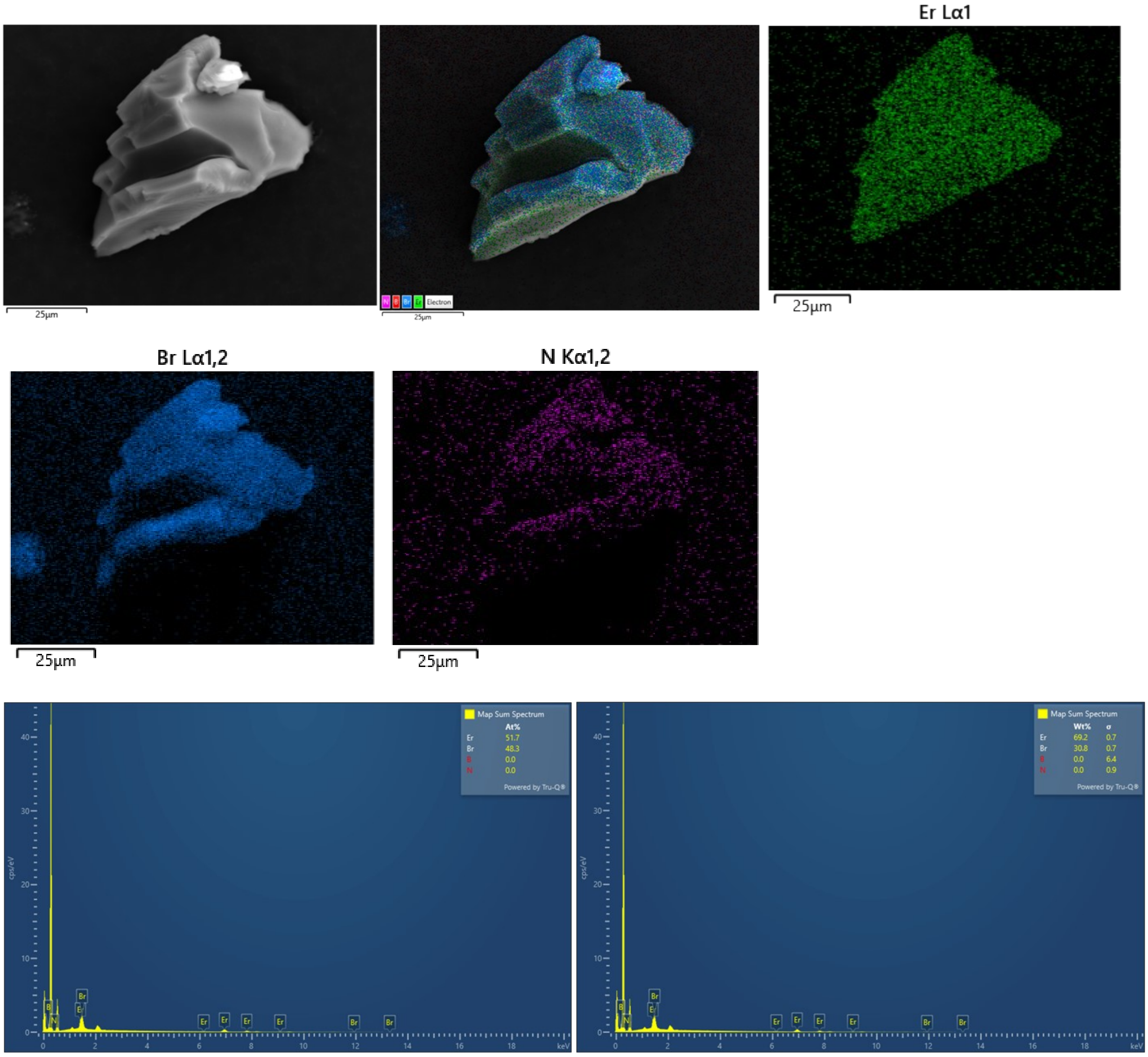

23.2 Figure S50. SEM and SEM-EDS mapping of LiCl@TB-MOF

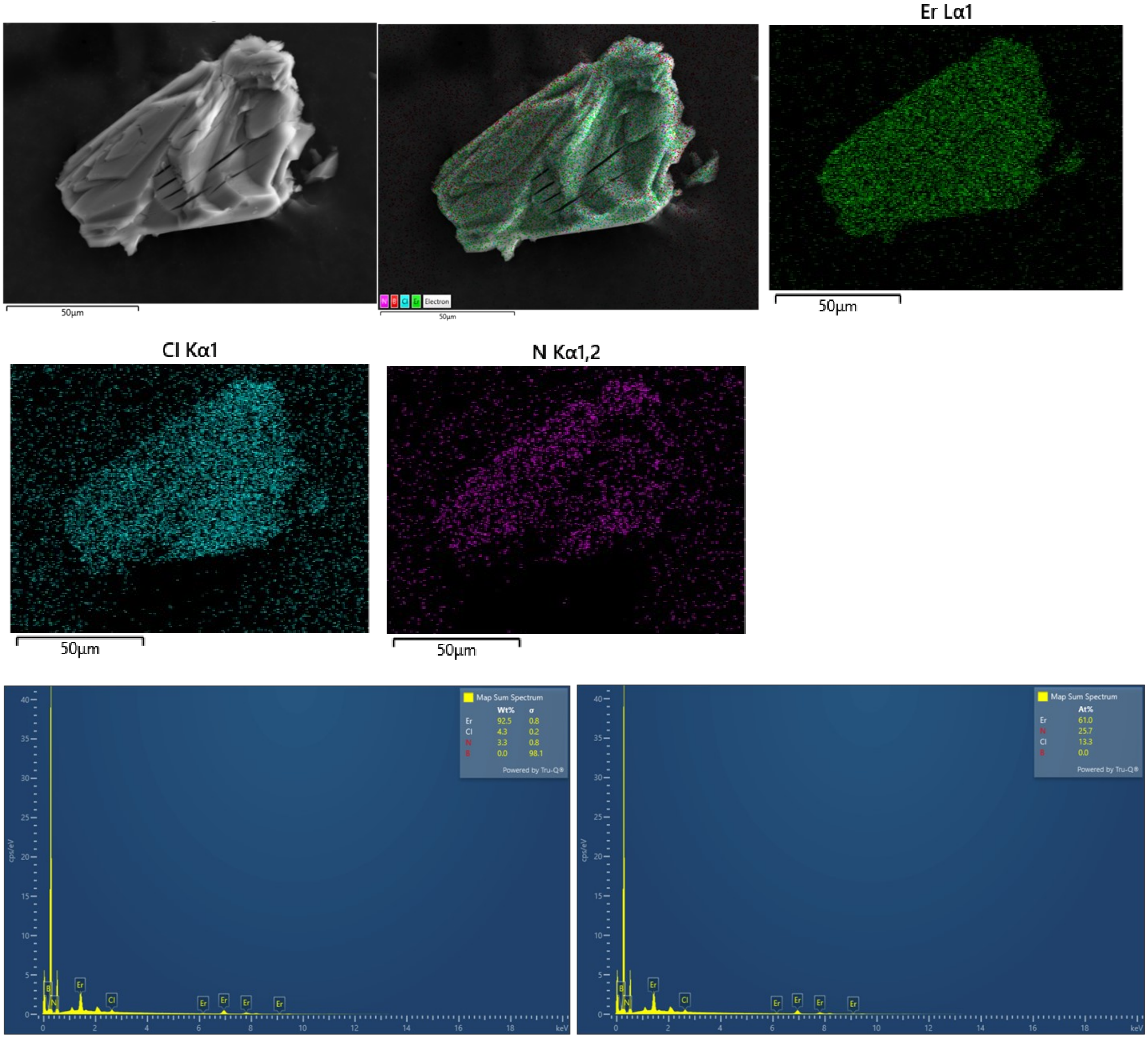

23.3 Figure S51. SEM and SEM-EDS mapping of $LiClO_4$@TB-MOF

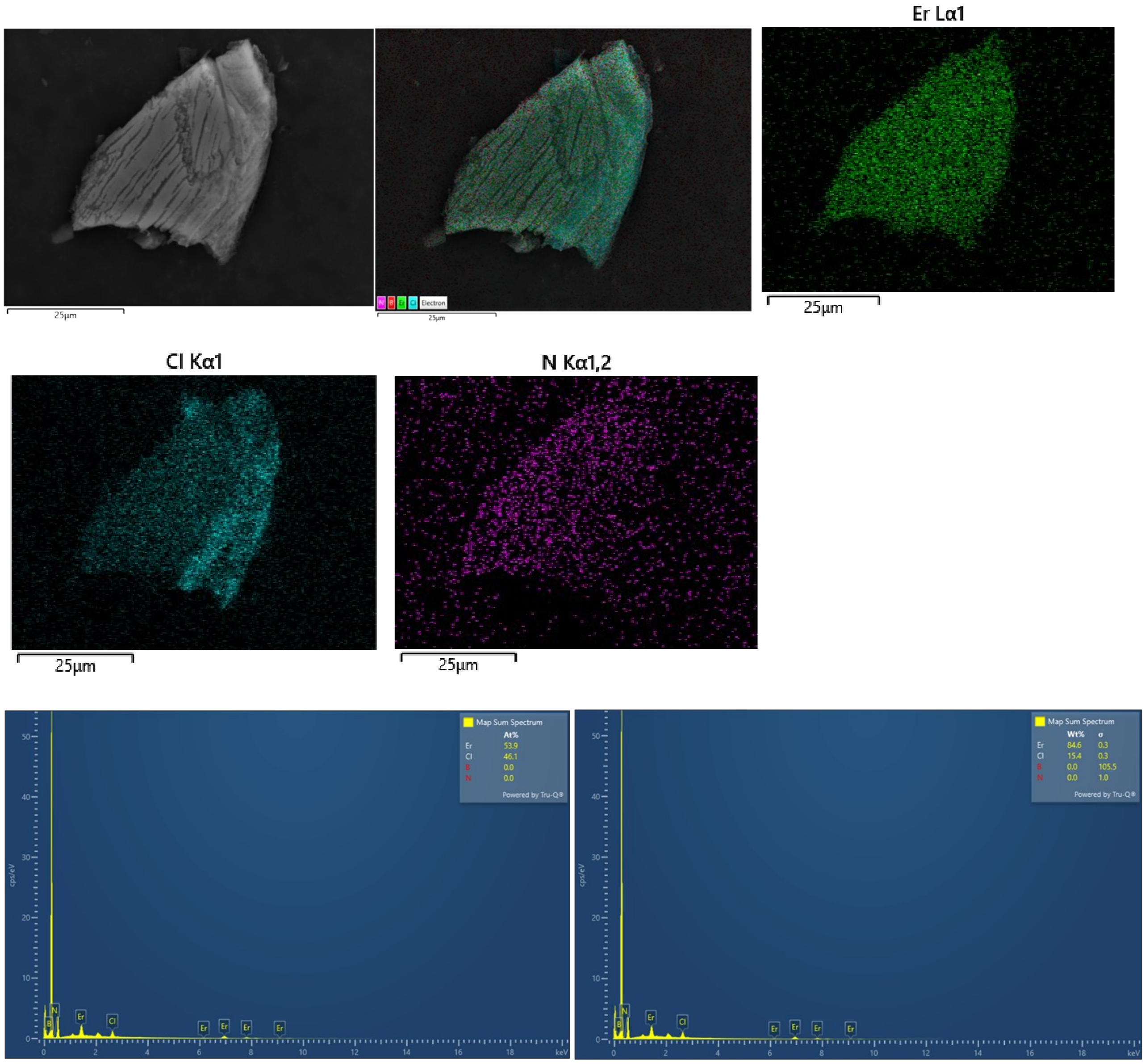

23.4 Figure S52. SEM and SEM-EDS mapping of LiOTf@TB-MOF

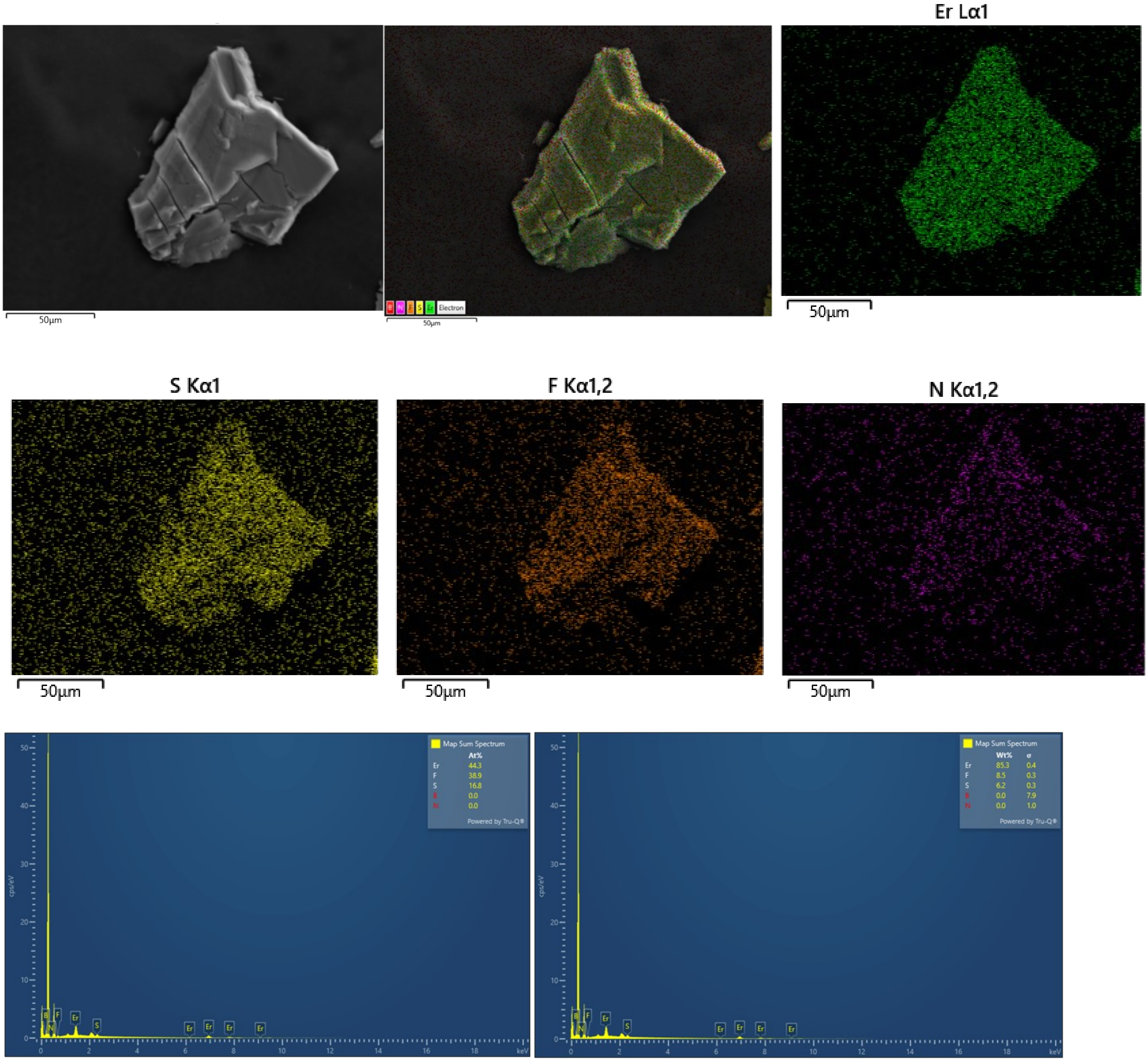

23.5 Figure S53. SEM and SEM-EDS mapping of NaI@TB-MOF

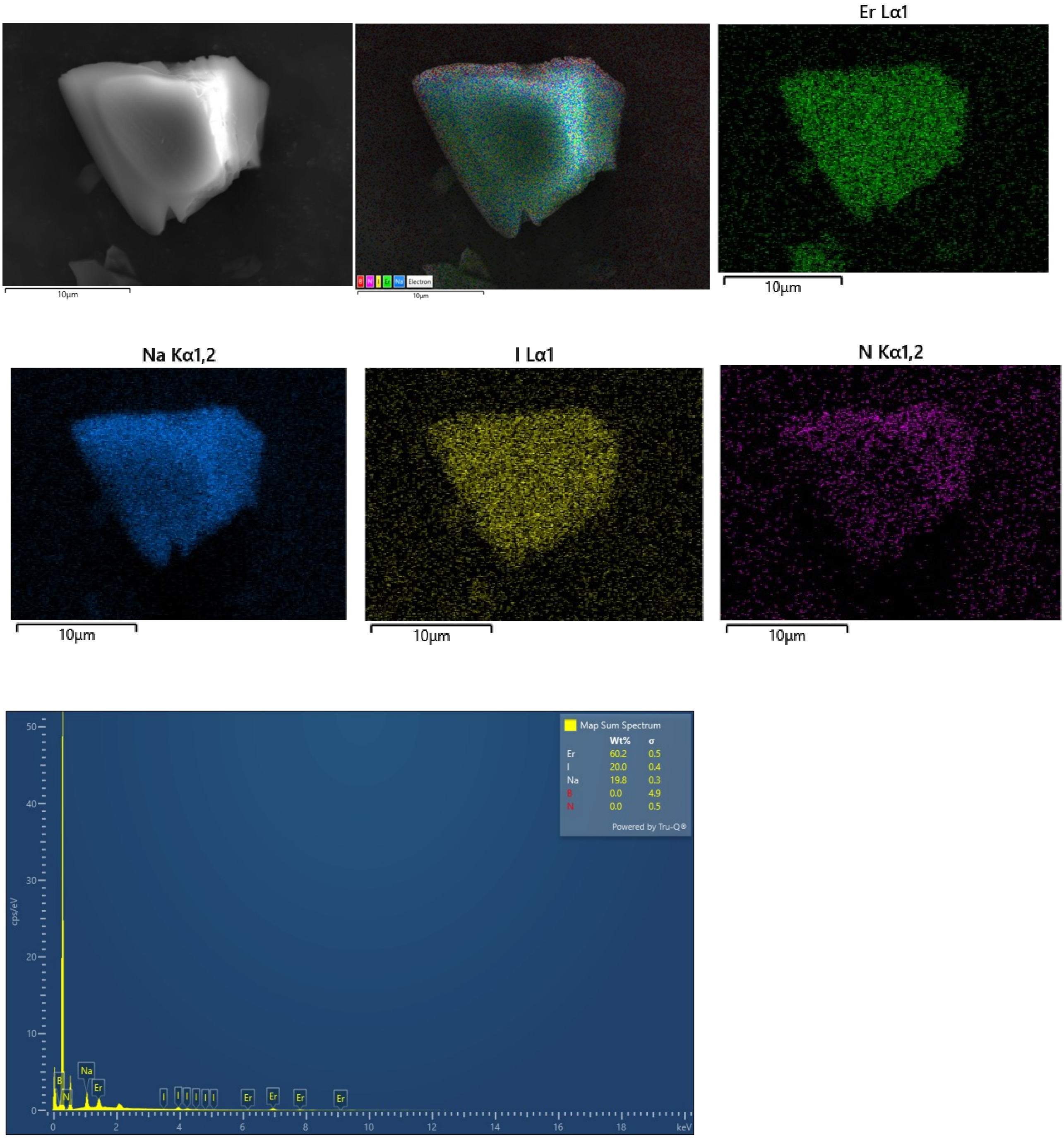

23.6 Figure S54. SEM and SEM-EDS mapping of KI@TB-MOF

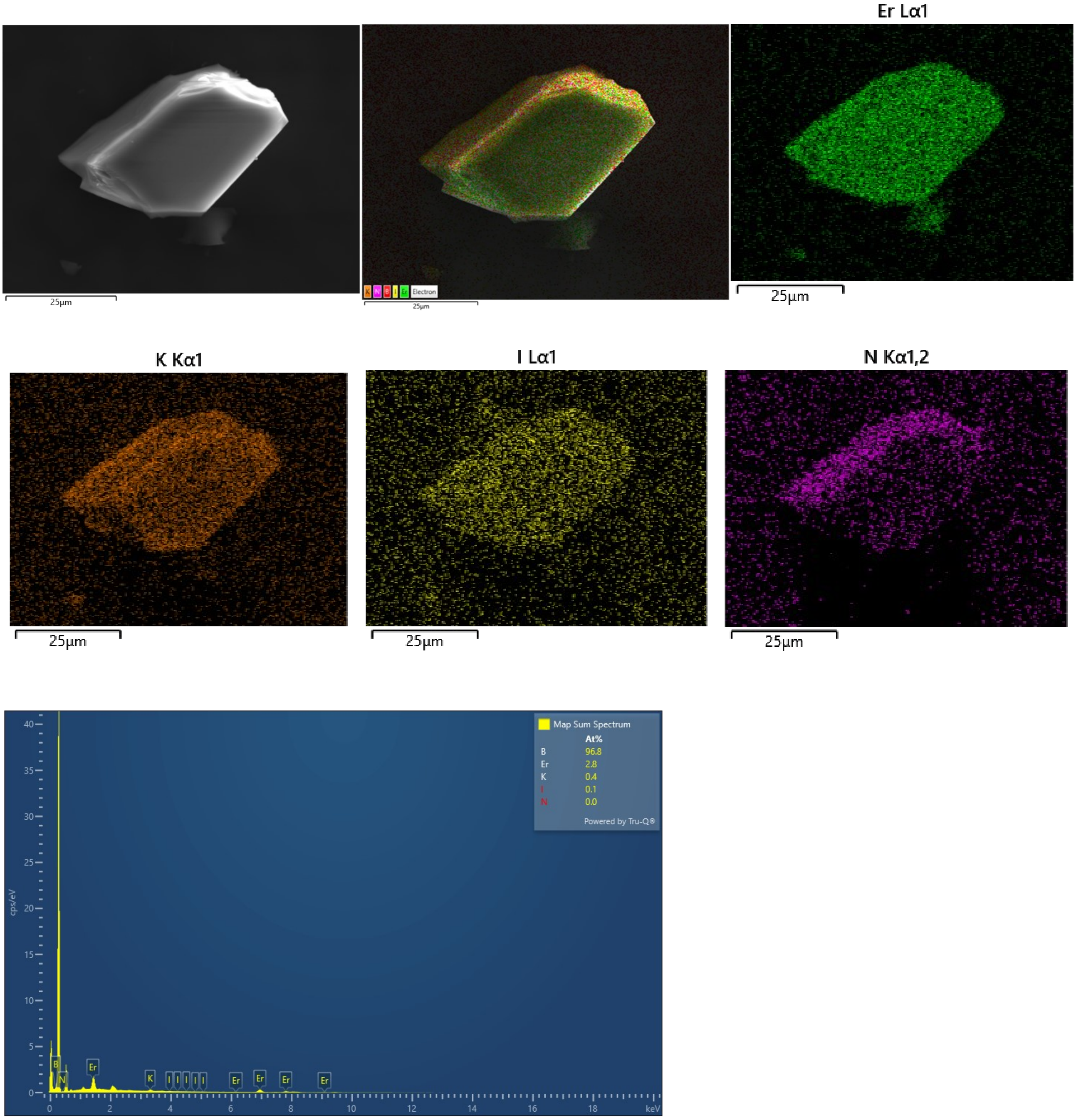

23.7 Figure S55. SEM and SEM-EDS mapping of CaI_2@TB-MOF

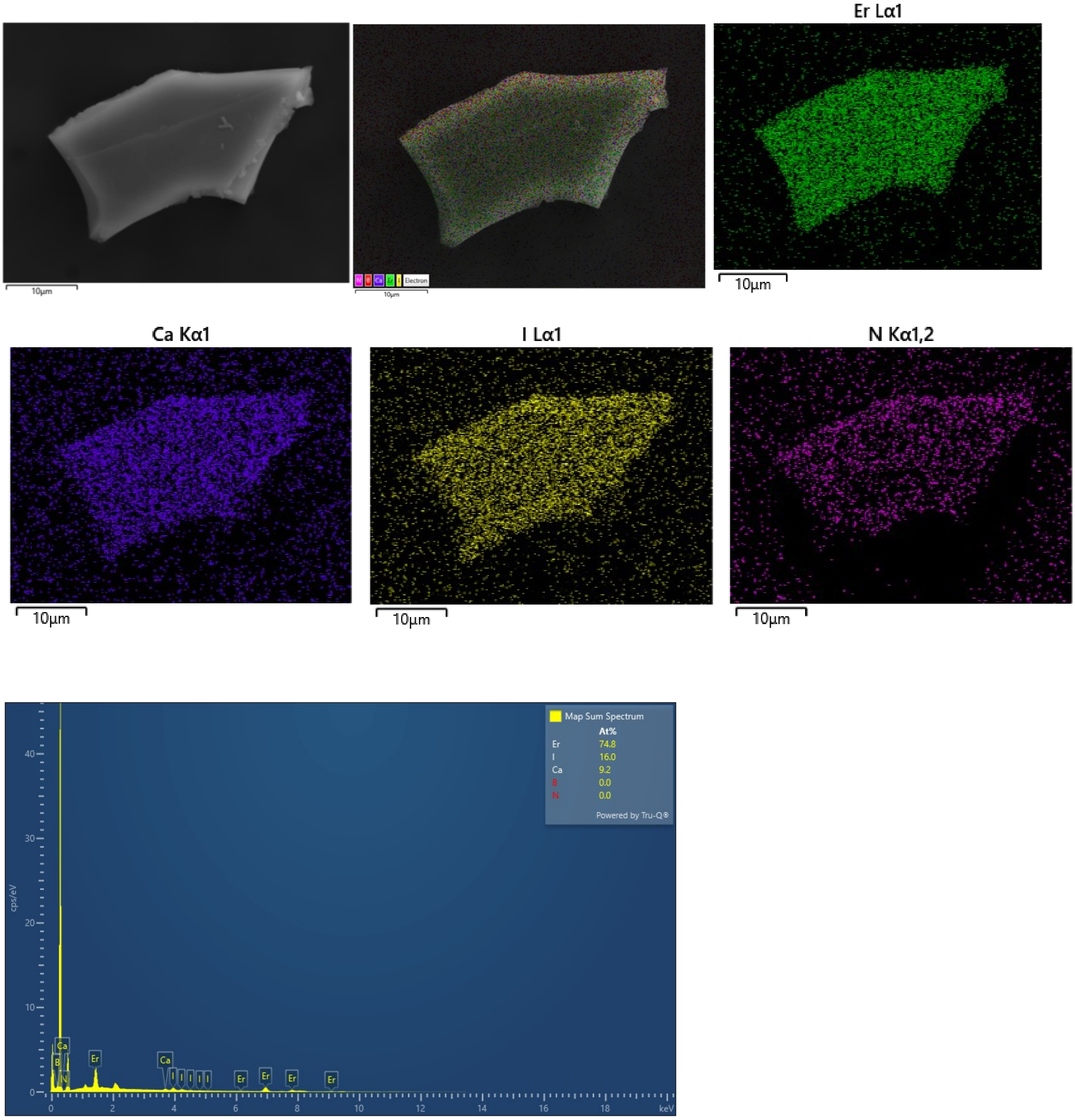

23.8 Figure S56. SEM and SEM-EDS mapping of MgI_2@TB-MOF

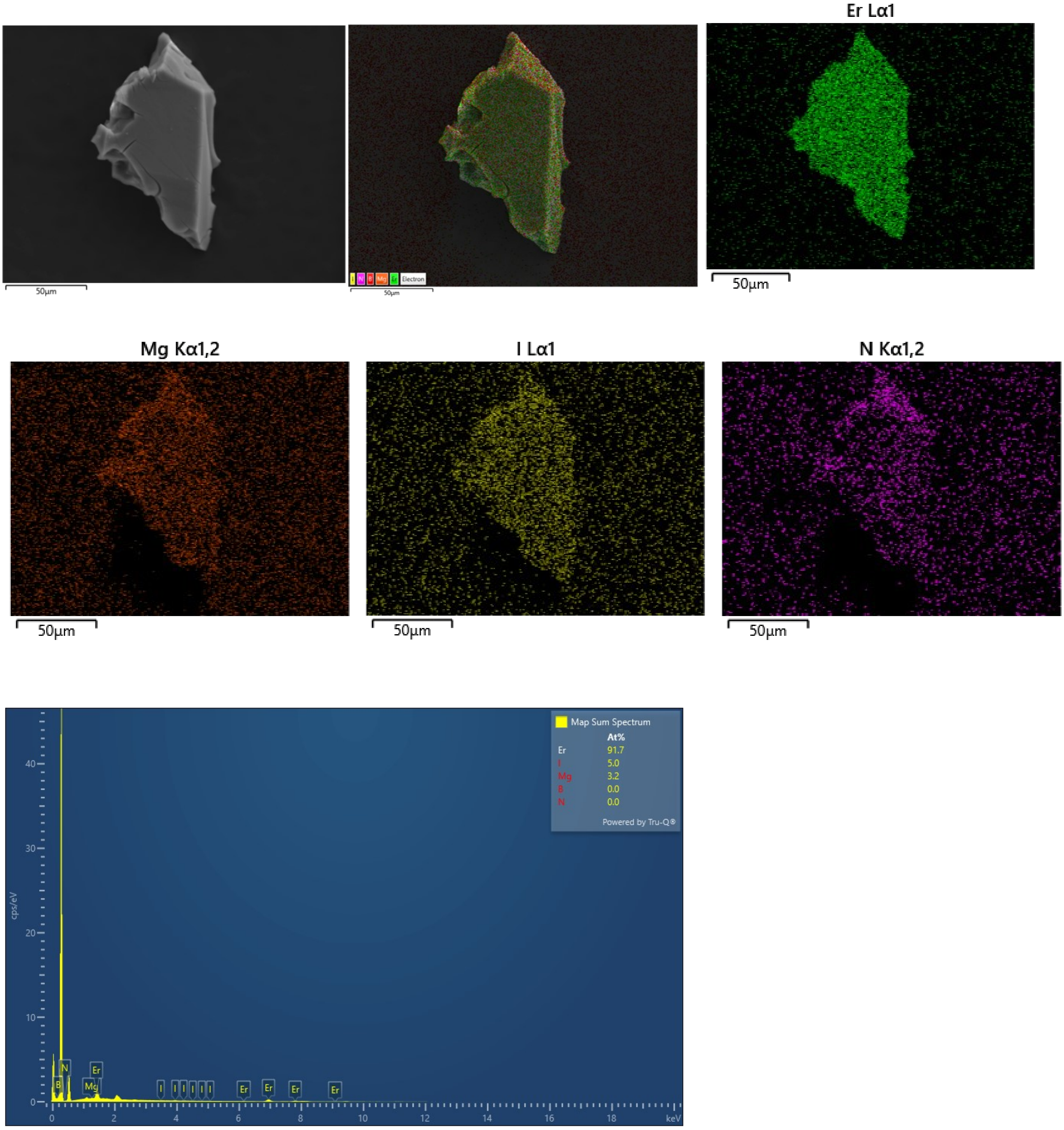

23.9 Figure S57. SEM and SEM-EDS mapping of ZnI_2@TB-MOF

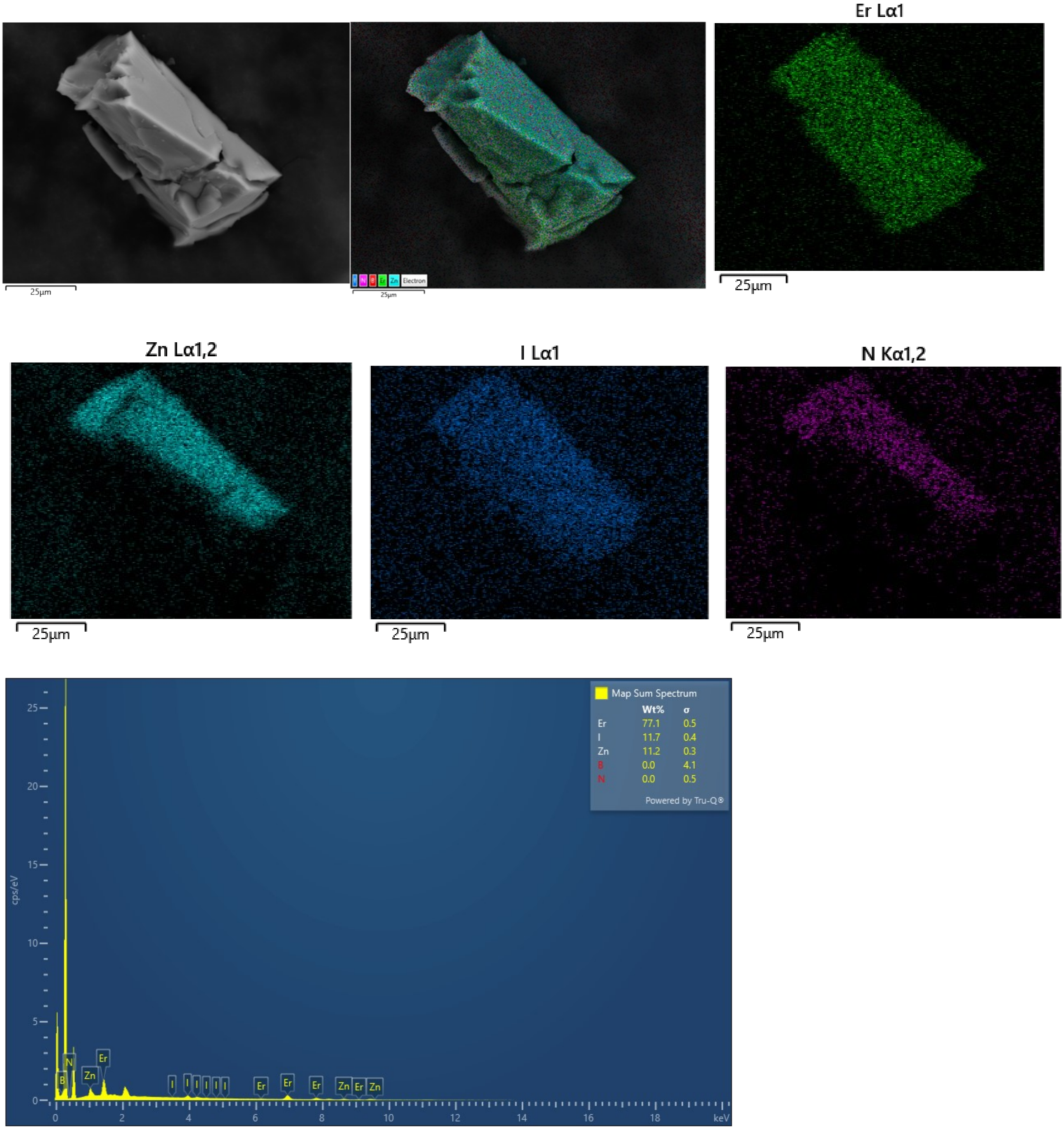

24 Figure S58 and S59. IR spectrum

24.1 Figure S58. IR spectrum of LiX@TB-MOF

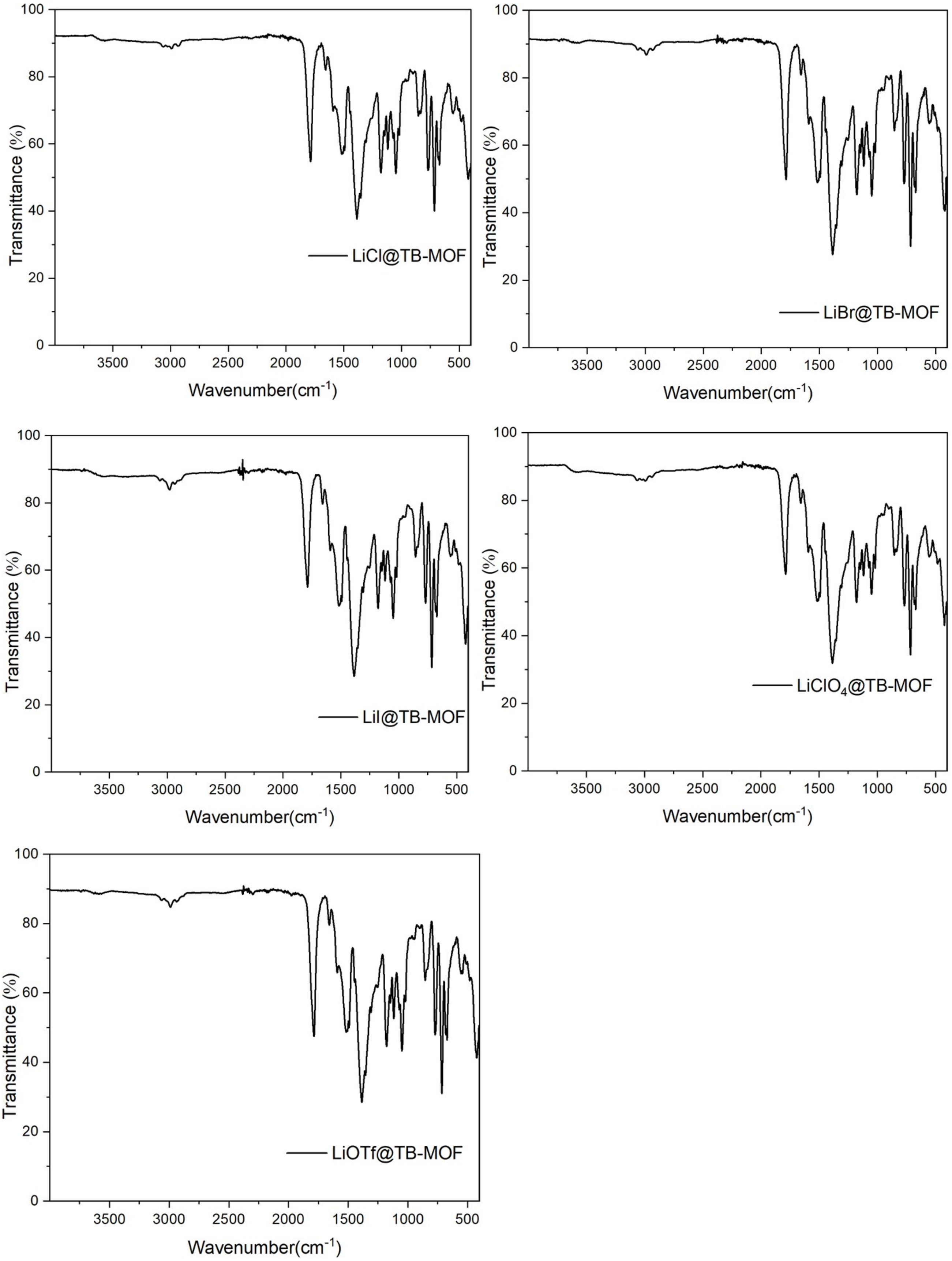

24.2 Figure S59. IR spectrum of MI@TB-MOF

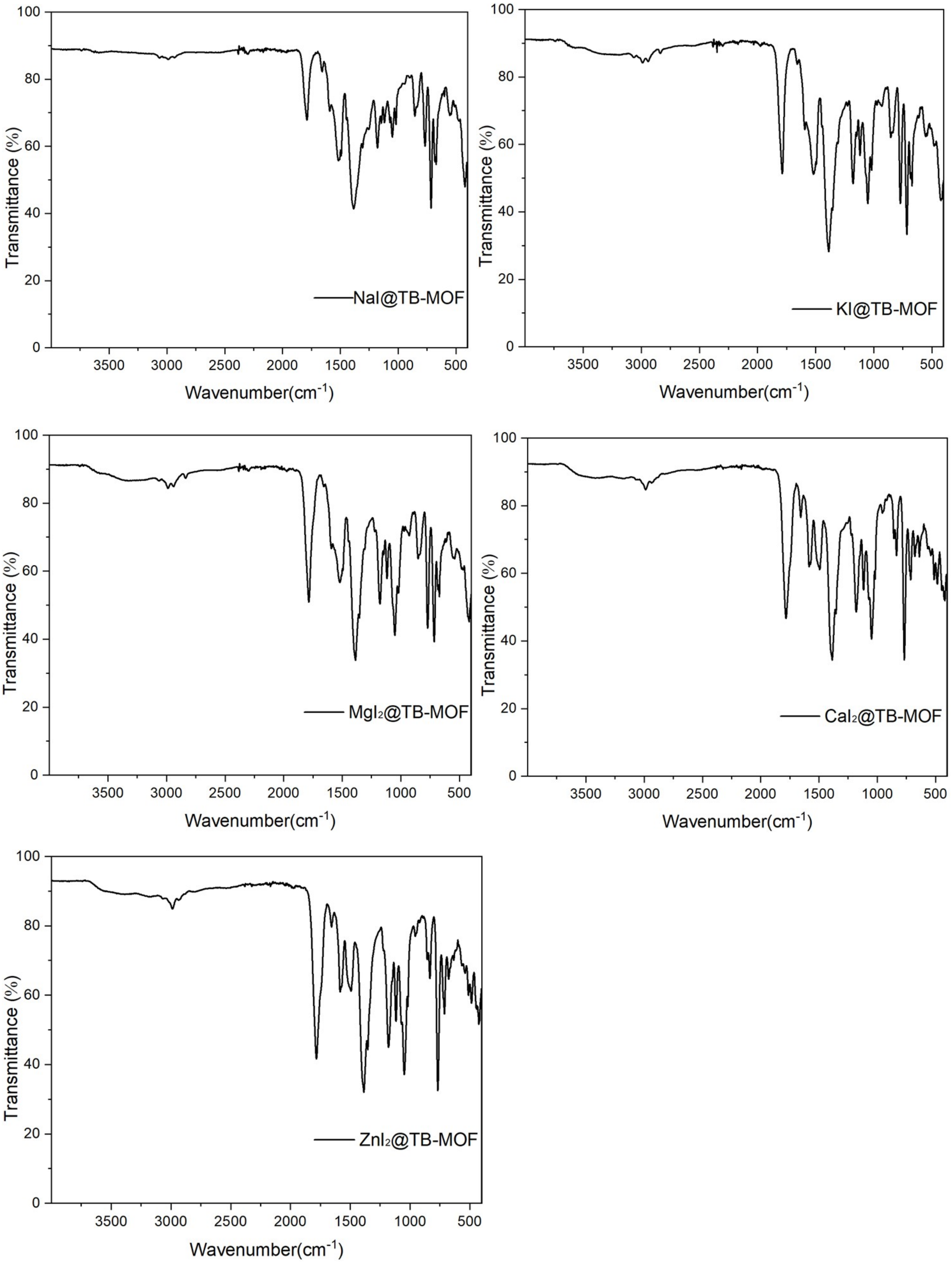

Supplement: SC-015-D4SC02861A-s001 [file SC-015-D4SC02861A-s001.pdf]
